# Optimizing the dynamics of protein expression

**DOI:** 10.1038/s41598-019-43857-5

**Published:** 2019-05-17

**Authors:** Jan-Hendrik Trösemeier, Sophia Rudorf, Holger Loessner, Benjamin Hofner, Andreas Reuter, Thomas Schulenborg, Ina Koch, Isabelle Bekeredjian-Ding, Reinhard Lipowsky, Christel Kamp

**Affiliations:** 1 Division of Microbiology, Paul Ehrlich Institut, Langen, Germany; 2 Max Planck Institute of Colloids and Interfaces, Potsdam-Golm Science Park, Potsdam, Germany; 3 Division of Allergology, Paul Ehrlich Institut, Langen, Germany; 4 Goethe University Frankfurt, Institute of Computer Science, Molecular Bioinformatics, Frankfurt am Main, Germany

**Keywords:** Computational models, Translation, Complexity, Dynamical systems, Computational science

## Abstract

Heterologously expressed genes require adaptation to the host organism to ensure adequate levels of protein synthesis, which is typically approached by replacing codons by the target organism’s preferred codons. In view of frequently encountered suboptimal outcomes we introduce the codon-specific elongation model (COSEM) as an alternative concept. COSEM simulates ribosome dynamics during mRNA translation and informs about protein synthesis rates per mRNA in an organism- and context-dependent way. Protein synthesis rates from COSEM are integrated with further relevant covariates such as translation accuracy into a protein expression score that we use for codon optimization. The scoring algorithm further enables fine-tuning of protein expression including deoptimization and is implemented in the software OCTOPOS. The protein expression score produces competitive predictions on proteomic data from prokaryotic, eukaryotic, and human expression systems. In addition, we optimized and tested heterologous expression of *manA* and *ova* genes in *Salmonella enterica* serovar Typhimurium. Superiority over standard methodology was demonstrated by a threefold increase in protein yield compared to wildtype and commercially optimized sequences.

## Introduction

The genetic code is redundant with up to six synonymous codons encoding the same amino acid. Although codon choice has no impact on the primary structure of proteins (i.e., the amino acid sequence), it affects cellular protein levels and the fitness of organisms as studied in bacteria (e.g. *Escherichia coli* or *Salmonella enterica* serovar Typhimurium), in eukaryotic microorganisms (such as *Saccharomyces cerevisiae*) as well as in human cell lines (e.g. HepG2, HeLa or HEK293)^1–4^. Codon bias – a preference for certain codons – is organism-specific and particularly pronounced in highly expressed genes. Therefore, artificially transferred genes need to be adequately adapted to the target organism. The codon adaptation index^5^ or related indices^2^ – which are based on the assumption that highly expressed genes are under selection pressure and, thus, already “optimal”^6^ – are valuable measures of “codon optimality”. Adaptation of codon bias to that of highly expressed genes often correlates with increased levels of protein expression as well as an overall increase in an organism’s fitness^7^. This finding is key to standard codon optimization procedures and is implemented in a variety of commonly used software tools such as GeneOptimizer^8^, JCat^9^, Optimizer^10^, Synthetic Gene Designer^11^, Codon Optimization OnLine (COOL)^12^, and EuGene^13^.

However, there is a serious drawback of these current state-of-the-art methods: Codon adaptation to biases seen in highly expressed genes is a purely heuristic approach. This approach does not provide a deeper understanding of the underlying processes and does not answer the question of optimality in a context-dependent and mechanistic manner. As a consequence, these heuristic codon optimization methods repeatedly cause unexpected or suboptimal outcomes^14^. This dilemma triggered a search for further heuristic covariates such as length of genes^6,15–17^, GC3 content and more complex mRNA sequence motifs as well as mRNA secondary structure^3,4,18–23^. In contrast, we address the question *how* codon bias affects protein expression through a codon-specific elongation model (COSEM). COSEM makes use of our understanding of protein synthesis and naturally opens a new avenue to overcome limitations of heuristic approaches.

This is done by modelling the process of mRNA translation as a key step in protein synthesis being performed by ribosomes. These molecular machines act as reading heads that move successively along the mRNA and decode its codon sequence into an amino acid chain. The corresponding sub-steps of codon-specific elongation by a single ribosome have been recently elucidated via a detailed Markov process^24,25^, see Supplementary Tables S1–S12. When several ribosomes move along the same mRNA, one has to take the mutual exclusion of the ribosomes into account and is then led to consider Totally Asymmetric Exclusion Processes (TASEPs)^26–38^.

COSEM combines codon-specific elongation (Supplementary Tables S7–S9) and mutual ribosomal exclusion with organism-specific translation-initiation rates and ribosome drop-off rates^39^ (Supplementary Tables S13 and S14) which provide the rates of ribosome attachment to the mRNA and ribosome loss resulting in pre-mature termination of protein synthesis. Consequentially, COSEM allows to study ribosome dynamics in a mechanistic manner and to assess the impact of codon bias on protein yield. Higher order effects such as tRNA recycling or density dependent drop-off rates can further be considered in advanced models. The integration of COSEM with additional sequence features relevant to protein synthesis into a protein expression score enables us to generate tailor-made gene sequences suitable to context-dependent requirements that may be optimized for accuracy and protein output or for alternative target functions. We validate our predictions of protein abundance on large scale data sets for *E. coli*, *S. cerevisiae*, and the human cell line HEK293 and demonstrate the protein expression score’s predictive power in comparison to state-of-the-art techniques. In addition, we choose two genes, *manA* and *ova*, for a more detailed analysis of expression in *S*. Typhimurium. Our approach outperforms presently used methods with respect to protein yield seen in synthetically designed variants of these genes.

## Results

### Codon-specific elongation model (COSEM)

#### Underlying processes and associated transition rates

**Figure 1:**
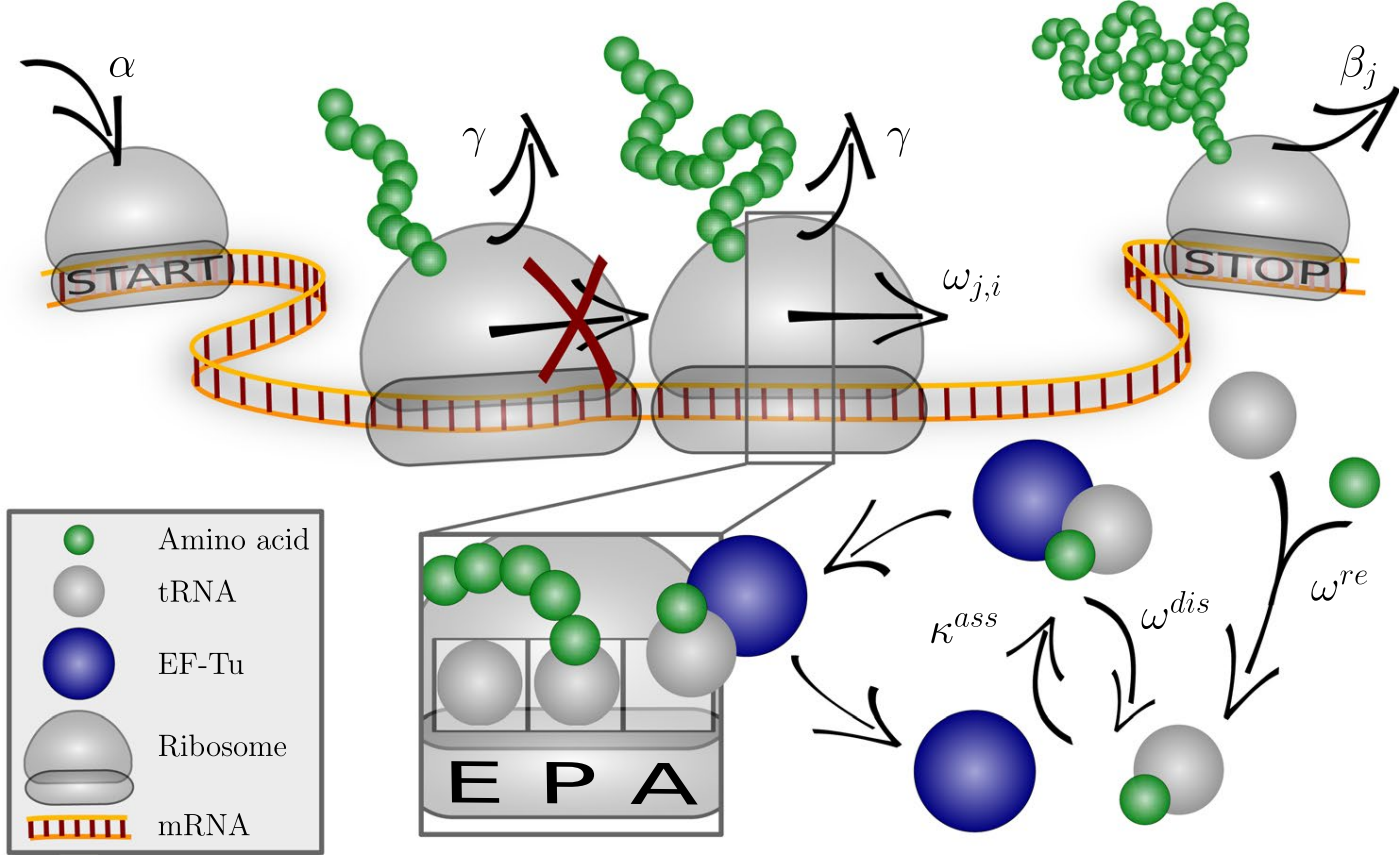
Sketch of the codon-specific elongation model (COSEM). Ribosomes attach to a codon sequence labeled by *j* with initiation rate *α*, which is determined by the ribosome concentration if the initiation site is not occupied, and move to the next position with the elongation rate *ω*_*j*,*i*_ specific to the codon at position *i* in the sequence *j* as well as to the organism under consideration. Codon-specific elongation rates *ω*_*j*,*i*_ are derived from the interaction between aa-tRNA (grey spheres, aminoacylated if associated with a green sphere), elongation factors (blue spheres) and GTP molecules (not shown) taking their organism-specific concentrations into account; for details see refs^24,25^ and Supplementary Information. mRNA translation may be terminated prematurely by ribosome drop-off which can occur at any codon with drop-off rate *γ*. Furthermore, ribosomes cannot overtake one another and can, at most, approach each other within one ribosomal footprint of length *d*. Finally, the ribosome detaches from the last codon of the sequence *j* with the rate *β*_*j*_, thereby completing the translation of this sequence.

The codon-specific elongation model (COSEM) considered here is sketched in Fig. 1. The translation process is initiated by ribosome attachment to the mRNA sequence *j* with the initiation rate *α*. Subsequently, ribosomes translate the mRNA with codon-specific elongation rates *ω*_*j*,*i*_, where *i* labels the codon position on the codon sequence *j*. Finally, ribosomes finish translation with the termination rate *β*_*j*_, corresponding to the elongation rate of the last codon, or leave the mRNA with the drop-off rate *γ* before reaching the last codon. When several ribosomes translate the same mRNA sequence, they cannot overtake each other. Furthermore, COSEM takes into account that each ribosome covers several codons and that each codon can be covered by only one ribosome at a time, where we take the ribosomal footprint to have a size of ten codons.

COSEM’s codon-specific elongation rates *ω*_*j*,*i*_ are calculated from a detailed Markov model reflecting the current biochemical knowledge of translation elongation^24,25^. In particular, the elongation rates depend on the concentrations of cognate, near-cognate, and non-cognate tRNAs and their competitive binding to the ribosomes, see Methods, Supplementary Information, and ref.^24^ for more information.

#### Dynamic regimes of simplified COSEM

**Figure 2:**
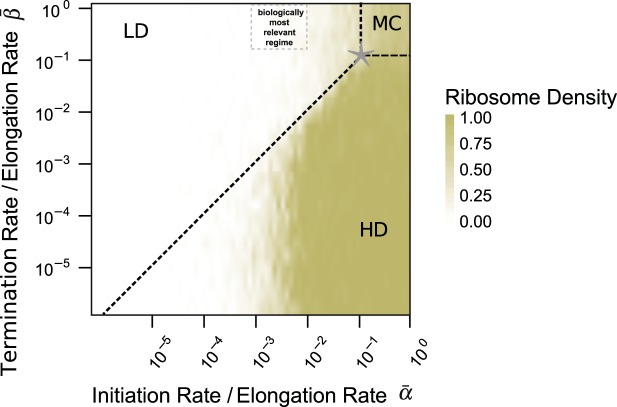
Dynamic regimes of COSEM with uniform elongation rate ***ω***_***j***,***i***_ ≡ ***ω***_***j***_. Average ribosome density as a function of reduced termination rate $\bar{\beta }={\beta }_{j}/{\omega }_{j}$ and reduced initiation rate $\bar{\alpha }=\alpha /{\omega }_{j}$. The phase diagram was computed for a synthetic mRNA with a length of 300 codons and a relative drop-off rate $\bar{\gamma }=\gamma /{\omega }_{j}=1.7\times {10}^{-4}$. With increasing initiation rate and decreasing termination rate the ribosome density shows a transition from a low (LD) to a high (HD) density regime with the latter being characterized by ribosome jamming. The broken diagonal line marks the expected phase boundary between the LD and HD regime for negligible ribosome drop-off ($\bar{\gamma }=0$). Ribosome drop-off reduces the ribosome density and thus the propensity for jamming. The transition to the maximum current (MC) regime occurs along the broken lines in the upper right corner of the phase diagram with the lower left corner of this regime indicated by a star (*). For a ribosomal footprint length of *d* = 10^40,41^ as used here, this corner has the coordinates ${\bar{\alpha }}^{\star }={\bar{\beta }}^{\star }=1/(\sqrt{d}+1)\simeq 0.24$. Because slow codons, i.e., codons *i* with low elongation rates *ω*_*j*,*i*_, are hardly observed at the end of a mRNA, the termination rate *β*_*j*_ should be comparable to the average elongation rate *ω*_*j*_ and the reduced termination rate $\bar{\beta }$ should be of the order of one. In addition, the reduced initiation rates $\bar{\alpha }$ are typically of the order of 10^−3^…10^−2^. Therefore, *in vivo*, ribosomal translation is expected to operate in the low ribosome density (LD) regime as indicated by the grey box.

To estimate the biological relevance of the mutual exclusion between translating ribosomes, we first consider a simplified version of the COSEM with a uniform, codon-independent elongation rate *ω*_*j*_. We can then choose the inverse rate 1/*ω*_*j*_ as the basic time scale for the translation of codon sequence *j* and determine the global phase diagram of this simplified COSEM as a function of the reduced initiation rate $\bar{\alpha }\equiv \alpha /{\omega }_{j}$ and the reduced termination rate $\bar{\beta }\equiv {\beta }_{j}/{\omega }_{j}$ with $0 < \bar{\alpha } < 1$ and $0 < \bar{\beta } < 1$, see Fig. 2. This phase diagram has been computed by stochastic simulations on a two-dimensional grid of $\bar{\alpha }$ and $\bar{\beta }$-values, using the Gillespie algorithm as described in the *Methods* section. For each parameter choice, we determined the steady state of the system and the corresponding ribosome density and ribosome current (or flux) profiles.

As shown in Fig. 2, the simplified COSEM leads, for different values of reduced initiation rate $\bar{\alpha }$ and reduced termination rate $\bar{\beta }$, to three dynamic regimes corresponding to the high density (HD), the low density (LD), and the maximal current (MC) phases. These different regimes can be distinguished by their steady state density profiles from which we compute the spatially averaged densities of the ribosomes as plotted in Fig. 2. In the low ribosome density phase, the reduced initiation rate $\bar{\alpha }$ is smaller than the reduced termination rate $\bar{\beta }$. Low ribosome density goes along with little collective dynamics such as jamming but also with a small current, *p*_*j*_, which is defined by the number of proteins produced per time and per mRNA. The opposite situation arises when the dynamics is limited by low termination rates or by bottlenecks of slow codons close to the terminal codon. The resulting high ribosome density phase is characterized by ribosome jamming and, thus, inefficient use of ribosomes. The dynamics in the maximal COSEM current phase (MC) is characterized by most efficient mRNA translation. The latter phase is reached when both initiation and termination rates are larger than the critical value ${\bar{\alpha }}^{\star }={\bar{\beta }}^{\star }$. Using known TASEP results^40,41^, one can estimate this critical value to be equal to ${\bar{\alpha }}^{\star }={\bar{\beta }}^{\star }=\frac{1}{\sqrt{d}+1}\simeq 0.24$, where the latter value corresponds to the ribosomal footprint *d* = 10.

#### Nonuniform elongation rates and biologically relevant dynamic regime

Biologically relevant mRNA sequences show heterogeneity in elongation rates which we acknowledge by approximating the uniform elongation rate *ω*_*j*_ of the simplified model by the harmonic mean of a sequence’s codon-specific elongation rates *ω*_*j*,*i*_, i.e. ${\langle \omega \rangle }_{j}^{h}=\frac{{n}_{j}}{{\sum }_{i=1}^{{n}_{j}}\,\frac{1}{{\omega }_{j,i}}}$. Furthermore, organism specific, average elongation rates 〈*ω*〉^*h*^ are obtained by averaging the rates *ω*_*j*_ over all sequences *j*. For *E. coli*, *S. cerevisiae*, and HEK293 cells, these doubly averaged elongation rates 〈*ω*〉^*h*^ are about 22 s^−1^, 33 s^−1^, and 6 s^−1^, see Supplementary Tables S1 and S13. While bottlenecks of low elongation rates can lead to shifts in the phase diagram and mixed phases^42,43^ our simulations showed only minor changes in the COSEM dynamics given heterogeneity in elongation rates as observed in biological systems (cf. Supplementary Tables S7–S9). Bottlenecks arising from slow codons are not observed at the end of a typical mRNA. Therefore, the termination rate *β* is expected to be comparable to the elongation rate 〈*ω*〉^*h*^ which implies that the reduced termination rate is $\bar{\beta }=\beta /{\langle \omega \rangle }^{h}\simeq 1$ The initiation rate *α* is estimated to vary in the range from 10^−2^ s^−1^ to 10^−1^ s^−1^ (cf. Materials and Methods). Combining this with the above estimates for 〈*ω*〉^*h*^, we conclude that the values of the reduced initiation rate $\bar{\alpha }=\alpha /{\langle \omega \rangle }^{h}$ vary in the range from 10^−3^ to 10^−2^. Because $\bar{\alpha }$ is much smaller than $\bar{\beta }$, the translation dynamics is limited by the initiation step and proceeds within the low ribosome density phase, cf. grey box in Fig. 2. Although low initiation rates will reduce the risk of ribosome jamming arising further downstream from slow codons, ribosome jamming will still be relevant for certain genes. Considering the coefficients of variation seen among codon-specific elongation rates in the studied gene sets from *E. coli*, *S. cerevisiae*, and HEK293 of 81%, 170%, and 52%, respectively, variability in initiation rates^36^ could also balance the variability seen among codon-specific elongation rates in different organisms and genes. COSEM as introduced in Fig. 1 captures the dynamics in all regimes of the phase diagram and provides an estimate of protein synthesis rates. Thus, in the following, for a given sequence *j*, we will now use the codon-specific elongation rates *ω*_*j*,*i*_ rather than their average values in order to compute the COSEM current *p*_*j*_, which describes the amount of protein synthesized per time and per mRNA labeled by *j*.

### Predicting protein expression

COSEM current *p*_*j*_ for a mRNA sequence *j* based on codon-specific elongation rates is a predictor for protein translation per time and can be expected to be the most relevant predictor for protein expression typically measured in terms of protein abundance^30,44^. To test this hypothesis and to improve the predictive power of the model, we integrate the COSEM current within a protein expression score (cf. Eqs (3–7) in the Materials and Methods section) that assesses the relative influence of features that are known or expected to impact on protein expression. Some features directly relate to the elongation process, such as the average elongation rate in the first 30 to 50 codons (acknowledging the ramp hypothesis of^45^), the occurrence and strength of bottlenecks (assessed as the slowest elongation rate within a 10 codon sliding window)^46^, and the accuracy of translation. Here, we define accuracy as the codon-specific probability for a ribosome to incorporate a tRNA that is cognate to the translated codon. To compute these codon-specific accuracies, we use a detailed Markov model for translation elongation, which takes into account the concentrations of cognate, near-cognate, and non-cognate tRNAs and from which we also obtained the codon-specific elongation rates, see Methods, Supplementary Information, and ref.^24^ for more information.

Further features are incorporated in the protein expression score to capture their influence on the structure and stability of the mRNA transcript. These include the mRNA folding energy in the first 30 codons of the 5′-end^47^, the overall GC content measured as the fraction of guanine and cytosine in the third nucleotide positions of all codons (GC3 content), and the number of hairpins within the first 30 codons of the 5′-end^47^. Finally, the mRNA transcript abundance as a prerequisite for protein expression is taken into account as well which together result in the protein expression score as summarized in Eq. (5).

**Figure 3:**
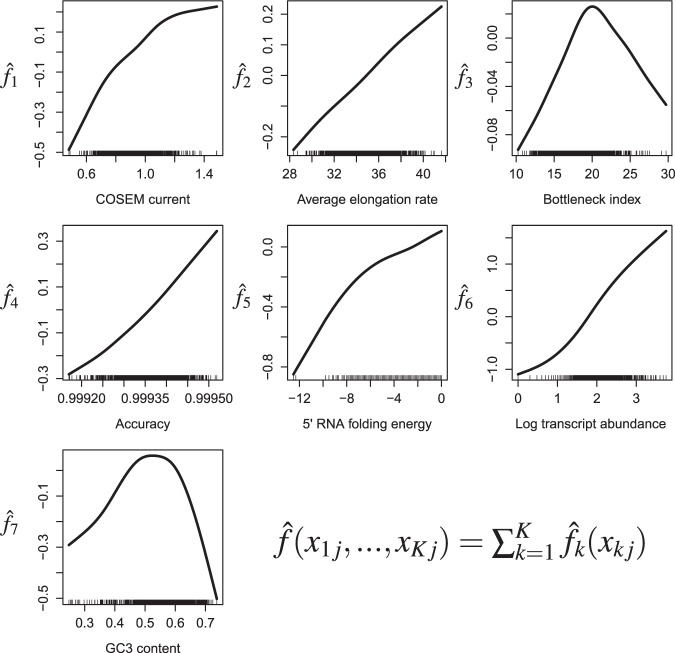
Determination of the protein expression score $\hat{{\boldsymbol{f}}}$ for *E. coli*. To estimate protein abundance, a generalized additive model as defined by Eqs (5–7) was fitted to protein abundance data of *E. coli* using model-based boosting methods. The fitted model is referred to as the protein expression score $\hat{f}$ and is the sum of seven partial functions ${\hat{f}}_{k}({x}_{k})$ (*k* = 1, 2, …, 7) corresponding to the solid lines in the seven panels. The tics along each *x*_*k*_-axis represent the specific values *x*_*kj*_ of feature *k* for the different sequences labeled by *j*. Monotonicity constraints were applied in the fitting procedure of the partial functions ${\hat{f}}_{k}$ of COSEM current, average elongation rate, and transcript abundance. Shown are only those seven features that were selected by the boosting algorithm to improve the correlation between the protein expression score $\hat{f}$ and the measured protein abundances. Generally, the fitted partial functions estimates $\hat{f}({x}_{k})$ follow intuition: An increase in COSEM current (protein per time), average elongation rate, accuracy, transcript abundance and folding energy (weaker folding) enhance the protein expression, whereas a balanced GC3 content appears to be favourable.

We derived all potential covariates as listed above for *E. coli*, *S. cerevisiae*, and HEK293 cells according to procedures described in the Materials and Methods section and Supplementary Information. To assess the relative importance of these diverse features, we fitted our model to protein abundance data using model based boosting methods^48,49^ (for details see Supplementary Figs S9–S11 and Materials and Methods). As shown on Fig. 3, the protein expression score is defined as the resulting function estimate $\hat{f}$, which is a superposition of partial functions ${\hat{f}}_{k}$ representing the additive contributions of the respective sequence features *k* to the estimate of protein abundance.

**Figure 4:**
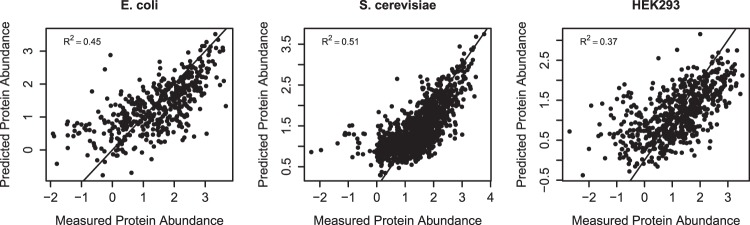
Comparing measured protein abundance with predicted protein expression for all *E. coli*, *S. cerevisiae*, and HEK293 genes where proteome data are available^50^ (cf. Supplementary Table S17). The coefficients of determination *R*^2^ for *E. coli*, *S. cerevisiae*, and HEK293 are 0.45 (95% CI 0.39–0.52), 0.51 (95% CI 0.47–0.54) and 0.37 (95% CI 0.32–0.43), and the number of coding sequences are 1563; 4479; 2136, respectively. Measured protein abundances are log-transformed values from PaxDb database’s common abundance metric in parts per million (ppm)^50^, for *E. coli* and *S. cerevisiae* there is a noticeable cutoff at 0 caused by a lower resolution limit of the measurement methods used. Predicted protein abundance is given in terms of the protein expression score as defined by Eqs (5–7).

Figure 4 shows protein abundances predicted by this protein expression score in comparison with measured protein abundances in *E. coli*, *S. cerevisiae* and HEK293 cells using protein and transcript abundance data from public databases (cf.^50^ and Supplementary Table S17). The coefficient of determination *R*^2^ is evaluated to assess the proportion of variance in protein abundances that can be explained by the protein expression score. As demonstrated in Fig. 4, 45%, 51%, and 37% of variation in protein expression in *E. coli*, *S. cerevisiae* and HEK293, respectively, can be explained by our protein expression score. Given least square regression of a simple linear model, we can assume the respective correlation coefficients *r* to be the root of the coefficient of determination *R*^2^, i.e. $\sqrt{{R}^{2}}=r=0.67,0.71,\text{and}\,0.61$, which compare well with correlation coefficients 0.29, 0.66–0.71, and 0.67 obtained in earlier studies^21,22,51^ on similar data sets, with improvements particularly for *E. coli*.

If only the COSEM current, i.e., the translation rate per mRNA transcript, and the transcript abundance are taken into account, the predictive power of the protein expression score is still high with correlation coefficients of 0.65, 0.67, and 0.59. This confirms the relevance of COSEM current in combination with mRNA levels for understanding total protein expression (cf. Supplementary Figs S15–S17, also noting the improvement over predictions based on mRNA levels alone as shown in Supplementary Fig. S18)^52^.

### Optimizing protein expression

**Figure 5:**
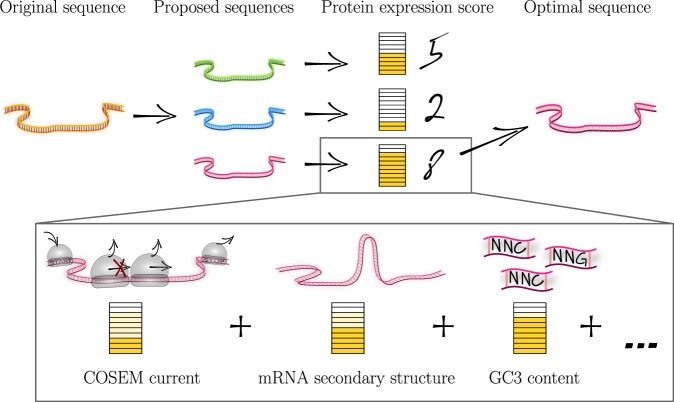
Optimizing protein expression on the basis of the protein expression score. The diagram sketches the flow of the codon optimization algorithm based on the predictive power of the protein expression score which is implemented in the software OCTOPOS: Sequences with alternative, synonymous codons are proposed from the original sequence and selected to maximize the protein expression score. Because of its modular structure in terms of feature specific partial functions, the protein expression score can be tuned as context-dependent target function allowing for flexible optimization schemes.

The predictive power of the protein expression score in Eq. (5) (as demonstrated in Fig. 4) allows us to address the inverse problem, i.e., to suggest mRNA sequences with codons that increase the protein expression score as compared to a reference or wild type sequence and are therefore likely to increase protein yield. This corresponds to an optimization of coding sequences with respect to protein yield. Figure 5 sketches the flow of our optimization algorithm, which selects sequences that maximize the protein expression score as a target function. The contributions of different sequence features *k* to the protein expression score can be adjusted through weighting of their partial functions ${\hat{f}}_{k}$ to define alternative target functions (cf. Eqs (3–8) and Fig. 3). In this way a sequence can, for example, be optimized for translation accuracy or deoptimized by minimizing the protein expression score.

We demonstrate this through an in-depth analysis of selected genes. Our first model gene encodes ovalbumin (*ova*), the main constituent of egg white and an important food and model allergen. Sufficient expression of *ova* after artificial transfer of the gene into host organisms such as *E. coli* or the closely related *S*. Typhimurium^53^ is relevant in biotechnological and medical applications. However, variants in which codon usage was adapted with standard procedures, i.e., GeneOptimizer (Geneart)^8^ using standard parameters, did not lead to increased protein expression compared to the wildtype variant in our experiments.

The second model gene *manA* encodes for phosphomannose isomerase, an essential enzyme for the mannose metabolism in *S*. Typhimurium^54^. Furthermore, a Δ*manA* mutant lacking *manA* shows a significant reduction in infectivity^54^. In spite of its key function for the *S*. Typhimurium metabolism and high expression levels we found that *manA* shows a comparably low codon adaptation index of 0.58.

For both genes, we created variants that are optimized for COSEM current and accuracy, a variant that was deoptimized on the basis of our model with respect to protein expression, as well as a variant with expected intermediate protein expression. For comparison we generated sequence variants optimized by GeneOptimizer (Geneart) with standard parameters. For *manA*, we also created variants with the original ramp of slow codons in the first 50 codons as this turned out to be one of the major determinants of expression strength for *manA* (cf. Supplementary Information, Figs S20 and S21). Additionally, we synthesized a variant with slow codons between *manA* secondary structure domains (cf. Supplementary Information, Table S19).

**Figure 6:**
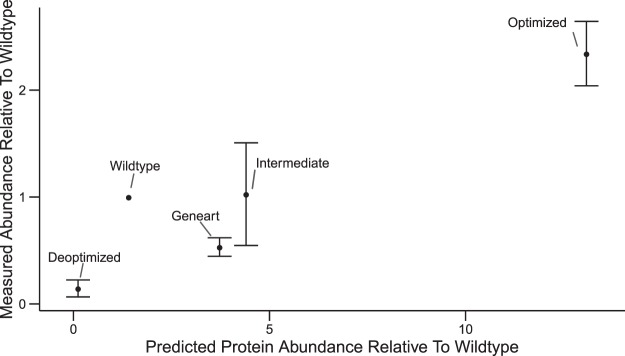
Protein expression of synthetic *ova* in *S*. Typhimurium. Measured protein abundance relative to wildtype compared to protein expression score relative to wildtype for *ova* variants. Ova protein levels were measured by Western blots (mean and standard deviation from Western blots of four biological replicates). The protein expression score is based on the function estimates shown in Fig. 3. setting the weight *v*_6_ for transcript levels equal to zero and the weights for all other features equal to one, because transcripts levels are undetermined at the time of sequence proposal.

**Figure 7:**
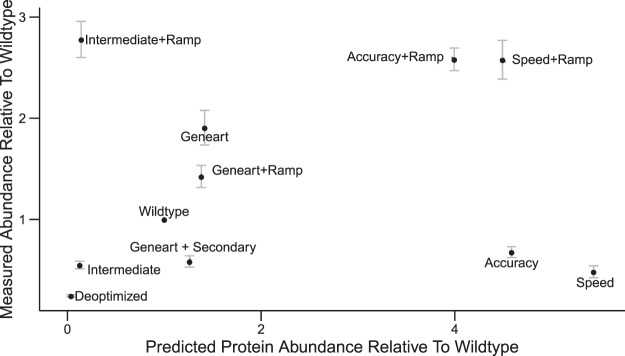
Comparison of measured and predicted protein abundances relative to wildtype for *manA* variants in *S*. Typhimurium. Measured ManA levels are weighted averages of mass spectrometry measurements (3 biological replicates, 3 digestion replicates, and 3 technical replicates each) and Western blots (3 to 5 replicates each), which correlate well (cf. Supplementary Fig. S24). Predicted protein levels are given in terms of the protein expression score relative to wildtype. The protein expression score is based on the function estimates shown in Fig. 3 setting the weight *v*_6_ for transcript levels equal to zero and the weights for all other features equal to one, because transcripts levels are undetermined at the time of sequence proposal.

We studied the protein expression in *S*. Typhimurium of the synthetic *ova* and *manA* sequences relative to the wildtype sequences in comparison to respective relative protein expression scores, see Figs 6 and 7. For *ova*, the deoptimized variant comes with the expected large decrease in expression, the optimized variant shows a three- to fourfold increase in expression compared to the wildtype. The synthetic *ova* version designed with the help of GeneOptimizer (Geneart) shows a slightly lower level of protein expression than the wildtype, whereas an additional variant with intermediate protein expression score shows the same expression as the wildtype. Deviations from the diagonal in Fig. 6 can arise from the non-negligible influence of the (undetermined) transcript levels on protein expression.

As shown in Fig. 7, the relative protein expression score for *manA* variants coincides well with measured protein levels for the de-optimized, wildtype and intermediate variants as well as for variants optimized by GeneOptimizer (Geneart), including those with an additional ramp of slow codons and slow codons between protein secondary structures. Choosing fast and accurate codons throughout the whole sequence does not increase protein expression (synthetic sequences *Accuracy* and *Speed* in Fig. 7). However, a marked increase in protein expression can be achieved by applying our optimization scheme while preserving the ramp of slow codons within the first 50 codons in the *manA* sequence. This highlights the relevance of a ramp of slow codons that is seen in the beginning of certain genes and the need to preserve this feature in these genes. Note that mRNA levels of the different *manA* variants were found not to differ significantly by quantitative real-time PCR (cf. Supplementary Information Fig. S25). Remarkably, measured growth rates of *S*. Typhimurium in minimal mannose medium correlate well with *manA* optimality and expression (cf. Supplementary Information Fig. S26).

Overall, the data imply that our approach can excel current state-of-the-art techniques for codon optimization. As a benefit over earlier approaches, our optimization scheme does not only propose optimal sequences but is also informative about protein expression levels through the protein expression score as shown in Figs 4, 6 and 7.

## Discussion

The success of synthetic gene expression depends on the adequate adaptation of the gene’s codon usage to the target organism. Current sequence optimization methods mainly focus on the introduction of codons that are preferred in the target organism’s highly expressed genes, combined with additional criteria largely based on mRNA motifs and structure. These heuristic methods do not allow for an explanation or for alternative solutions in cases of failure.

This issue is addressed by the codon-specific elongation model (COSEM) introduced here as a model of protein expression at the level of mRNA translation. The integration of COSEM with further covariates into a protein expression score leads to state-of-the-art predictions of protein expression as exemplified for *E. coli*, *S. cerevisiae*, and human HEK293 cells. This paves the way for a new strategy of codon optimization for which we show superiority in two exemplary cases, ManA and Ova expressed in *S*. Typhimurium.

In contrast to heuristic approaches, our optimization scheme is based on current knowledge about protein expression mechanisms. Thus, it allows for an optimization of specific features such as translation accuracy or protein expression not addressed by the algorithms currently in use. As our approach is not only informative about optimal codons but also about codon-specific protein synthesis rates it provides a tool to modulate protein levels within cells. This can change cellular function which may lead to manifold biotechnological applications. One application may be the deoptimizaton for specific target functions such as expression or accuracy of specific proteins through a minimization of the (adequately weighted) protein expression score. This can be a valuable feature for engineering of attenuated pathogens in vaccine design^55–57^. In other situations of synthetic biology, e.g. the design of synthetic metabolic pathways or artificial regulatory circuits, fine-tuning of protein expression levels as facilitated by our method is often essential^58–60^.

The design of genetic sequences based on a model of mRNA translation is a conceptually new approach. Therefore, it does not only bring gradual improvements but introduces qualitatively new aspects to the field of codon-optimization. The parameterization of the protein expression score facilitates a direct adaptation to other target systems including different cell types and even cells in specific environments or conditions. The modularity of the protein expression score allows to consider additional features that might be relevant in these conditions^21,22^. The method itself is open to further improvements, in particular by taking additional aspects of protein expression into account for the derivation of the protein expression score. The COSEM module can gain in biological realism by considering gene-specific initiation rates^36^. As codon choice can impact on the timing for protein folding^61–63^, protein secondary (and potentially tertiary) structure may also have to be considered and may be reflected by codon subsequences rather than codon frequencies^64^. The interplay between translation and mRNA degradation^65^ might introduce non-trivial feedbacks to protein expression levels as may resource limitations^66^. Also, the protein expression score can be adapted to take specific features – such as a ramp of slow codons – stronger into account for particular groups of genes, as exemplified here for the *manA* gene. Our approach can also be combined with other algorithms that address other important aspects of sequence optimization such as the influence of the ribosomal binding site^23^, mRNA secondary structure^67^ or protein folding kinetics^68^, tailored to the respective genomic background. Eventually, the integration of such interconnected aspects into a combined workflow for codon optimization will allow the design of optimal coding sequences matching the exact requirements for protein expression.

In summary, we have demonstrated the predictive power of the protein expression score as well as the benefits and potentials of a codon optimization scheme based on a model of protein expression. We expect that the understanding of protein expression in codon optimization schemes will substantially improve the current state of the art in the field. The presented tools have in particular the potential to advance the design of precisely tailored genes for a wide range of applications in synthetic biology.

## Methods

### Codon-specific elongation model (COSEM)

#### Stochastic simulation of COSEM

The codon-specific elongation model (COSEM) for protein translation is sketched in Fig. 1. The ribosome attaches to the mRNA *j* with an initiation rate *α*. It covers *d* codons corresponding to the ribosomal footprint length and advances with a position-dependent elongation rate *ω*_*j*,*i*_ depending on the *i*-th translated codon in sequence *j*. If a ribosome is selected for movement but is blocked by a preceding ribosome the blocked ribosome moves immediately as soon as preceding ribosome advances^36^. Thus, in the latter case, two adjacent ribosomes move forward simultaneously. Protein elongation is in competition with ribosome drop-off that occurs with rate *γ*.

To simulate the translation of proteins and to determine the COSEM current *p*_*j*_ of a sequence *j*, we use a Gillespie-type scheme^69–72^, apart from the additional rule for simultaneous forward movement of two adjacent ribosomes. Thus, consider *N*_1_ ribosomes bound to the codon sequence *j* which form a certain ribosome configuration *C*_1_. This configurations is defined by the positions of the ribosomal A-sites at the codons *i*_*n*_ with *n* = 1, …, *N*_1_. Starting from the configuration *C*_1_, we can reach a variety of new configurations *C*_2_ by elementary transitions corresponding to the forward steps of single ribosome, drop-off of single ribosomes, release of a ribosome from the terminal codon, and addition of a new ribosome to the first codon. Let *M* be the number of possible new configurations *C*_2_(*m*) with *m* = 1, …, *M* and *q*_*m*_ the corresponding transition rates from *C*_1_ to *C*_2_(*m*). All of these rates can be expressed in terms of the codon-specific elongation rates ${\omega }_{j,{i}_{n}}$, the drop-off rate *γ*, the termination rate *β*_*j*_, and the initiation rate *α*. The probability to undergo the transition from *C*_1_ to *C*_2_(*m*) is then given by

1 $${\bar{q}}_{m}={q}_{m}/{Q}_{1}\,{\rm{with}}\,{Q}_{1}\equiv \mathop{\sum }\limits_{m{\prime} =1}^{M}\,{q}_{m{\prime} }.$$

The new configuration *C*_2_(*m*) is now chosen randomly with probability ${\bar{q}}_{m}$. The chosen transition is executed and the simulation time is advanced by 1/*Q*_1_ times the logarithm of the inverse of a uniform random number. If initiation or elongation is restrained by a preceding ribosome the event is executed as soon as this moves forward. For extended error checking, simulations were repeated using an alternative algorithm, in which time is advanced with a small increment at every iteration step. Here, the probability for each event to occur within the time interval is approximated by the product of the corresponding rate and the time interval (for sufficiently small time intervals). Source code is available upon request.

#### Relation between COSEM current and codon-specific elongation rates

In general, maximizing the codon-specific elongation rates *ω*_*j*,*i*_ in a sequence *j* maximizes the amount of protein produced per mRNA and time, i.e., the COSEM current *p*_*j*_. This becomes evident from studying the average time *t*_*j*_ for synthesizing a protein by translating the codon sequence *j*. If we ignore the mutual exclusion of the ribosomes and their drop-off, the average synthesis time *t*_*j*_ is given by

2 $${t}_{j}\simeq {t}_{{\rm{in}}}+\mathop{\sum }\limits_{i=1}^{{n}_{j}}\,\frac{1}{{\omega }_{j,i}}+{t}_{{\rm{te}}}$$

where *t*_in_ is the average initiation time and *t*_te_ the average termination time. Figure S2 shows that this simple relation holds only in the initiation limited LD regime whereas collective ribosome dynamics increase the synthesis time beyond this lower estimate. To our knowledge, there is no simple relation between the set of elongation rates {*ω*_*j*,*i*_} and the COSEM current *p*_*j*_, particularly if the dynamic is not limited by low initiation rates.

Especially in the presence of bottlenecks, i.e., regions of “slow” codons, an increase in the average elongation rate ${\langle \omega \rangle }_{j}=\mathop{\sum }\limits_{i}^{{n}_{j}}\,\frac{{\omega }_{j,i}}{{n}_{j}}$ for a sequence *j* of *n*_*j*_ codons might not directly relate to an increase in protein production. Thus, optimizing the COSEM current *p*_*j*_ instead of 〈*ω*〉_*j*_ can be particularly relevant when considering the trade-off between fast and accurate codons (cf. Supplementary Figs S3–S5). Note that average elongation rates could similarly be determined in terms of the harmonic instead of the arithmetic mean. While both means strongly correlate, the arithmetic mean tends to give better predictive power in the protein expression score as it correlates less with the COSEM current, i.e. may contribute more additional information (cf. Supplementary Figs S12–S14, S19).

#### Model parameters

COSEM dynamics and the protein expression score depend on a variety of organism-specific parameters for which estimates and derivations are summarized below. We calculated codon-specific elongation rates and accuracies for translation in *E. coli* by minimizing the kinetic distance between a set of measured *in-vitro* rates and predicted rates compatible with translation *in-vivo* as described in^24,25^. To obtain codon-specific elongation rates and accuracies for HEK293 and *S. cerevisiae* cells, we applied the same method using parameters listed in Supplementary Tables S1–S6. Briefly, translation of a codon is described by a Markov process. Experimentally determined *in-vitro* values of transition rates are used to predict a set of *in-vivo* transition rates compatible with the organism- and growth rate-dependent overall rate of protein synthesis. Furthermore, we assume that the codon-specific elongation rates and accuracies depend on the concentrations of free ternary complexes via competition of cognate, near-cognate, and non-cognate ternary complexes at the ribosomes’ binding sites. From codon usages and measured or estimated tRNA abundances the concentrations of the corresponding ternary complexes are calculated by taking into account the recharging of tRNAs by aminoacyl tRNA synthetases. Current calculations are based on averaged concentrations and might be further improved by considering spatial effects^73^.

Accuracies are determined as the probabilities to incorporate cognate and not near-cognate tRNAs, regardless whether the near-cognate tRNAs carry the same amino acids as the cognate tRNAs or not. A detailed listing of parameters and all codon-specific elongation rates and accuracies can be found in Supplementary Tables S1–S12. Also a list of cognate, near-cognate (possibly missense), or non-cognate codons is given in Supplementary Tables S5 and S6.

We assume a ribosome drop-off probability of 3 × 10^−4^ per codon. Considering the average elongation rates for *E. coli*, *S. cerevisiae*, and HEK293 cells of 22 s^−1^, 33 s^−1^, and 6 s^−1^ per codon, respectively, allows to derive drop-off rates *γ* in the range of 0.001 s^−1^ to 0.01 s^−1^ (cf. Supplementary Table S13 and references therein).

Translation initiation rates are hard to determine experimentally. For *E. coli* exists a vague estimate of *γ* = 5 min^−1^ ≈ 0.083 s^−1^^ 74,75^. This goes in line with model-inferred estimates for *E. coli*, *S. cerevisiae*, and human HeLa cell lines of the order of 0.01 s^−1^ to 0.1 s^−1^ ^36,44,51,76^. As an alternative, we inferred self-consistent parameter ranges for initiation rates by maximizing the correlation between COSEM current and observed protein levels (cf. Supplementary Figs S6–S8, Table S14). Initiation rates were estimated by this method to be larger than 0.01 s^−1^ and ranged up to 100 s^−1^, driving COSEM into the elongation-limited regime. While different initiation rates may be suitable in different contexts or genes, we focus on the latter estimate (cf. Supplementary Table 14) for optimization purposes to achieve the highest predictive power.

### Protein expression score

#### Statistical modelling of protein abundance

In general, statistical modelling addresses the relation between a certain outcome variable *y* and a set of predictor variables or features, **x** ≡ (*x*_1_, *x*_2_, …, *x*_*K*_). In the case of protein expression as considered here, the outcome variable is the logarithm of the protein abundance derived from different mRNA sequences labeled by *j*. Thus, the outcome variable *y* has the value *y*_*j*_ for mRNA sequence *j*. We have introduced the COSEM current *p*_*j*_ of a sequence *j* as a predictor for protein expression and abundance which we will complement with additional predictor variables (sequence features) as listed below.

For each feature *x*_*k*_, we introduce a partial predictor function *f*_*k*_(*x*_*k*_) that describes the effect of the feature *x*_*k*_ on the logarithmic protein abundance. The functions *f*_*k*_(*x*_*k*_) are modelled by base-learners which determine their functional form^48,49^ as described in the *Technical Details* below. Each set of partial predictor functions *f*_*k*_(*x*_*k*_) with *k* = 1, 2, …, *K* defines a prediction model of the general form

3 $$f({\bf{x}})=\mathop{\sum }\limits_{k=1}^{K}\,{f}_{k}({x}_{k})\,({\rm{additive}}\,{\rm{statistical}}\,{\rm{model}}).$$

which represents our prediction model for the logarithm of protein abundance.

Because the values *f*(***x***) depend on the functional forms of the partial predictor functions *f*_*k*_(*x*_*k*_), we aim to optimize these functional forms in order to obtain the best prediction model (regression function) $\hat{f}({\bf{x}})$ which represents our protein expression score. Starting with plausible assumptions about the functional forms of the partial predictor functions *f*_*k*_(*x*_*k*_), these functional forms are varied in order to minimize the squared error loss defined by

4 $$\Lambda \equiv \frac{1}{J}\mathop{\sum }\limits_{j=1}^{J}\,{[{y}_{j}-f({{\bf{x}}}_{j})]}^{2}$$

where the sum includes a test sample of observed values *y*_*j*_ of the outcome variable of mRNA sequences labeled by *j* = 1, …, *J*, i.e. the observed logarithmic protein abundances. In practise, the functional variation of the partial predictor functions is performed in an iterative manner, using boosting methods as described below, until the squared error loss saturates. As a result of this minimization procedure, we obtain partial predictor function estimates${\hat{f}}_{k}({x}_{k})$. In line with common statistics notation, we distinguish the function estimates that minimize Eq. (4) by the hat symbol which defines our protein expression score

5 $$\hat{f}({\bf{x}})=\mathop{\sum }\limits_{k=1}^{K}\,{\hat{f}}_{k}({x}_{k}).$$

When applied to a certain sequence *j* with specific features *x*_*j*_, we obtain the value

6 $$\hat{f}({{\bf{x}}}_{j})=\mathop{\sum }\limits_{k=1}^{K}\,{\hat{f}}_{k}({x}_{kj})$$

of the protein expression score for sequence *j*. In Fig. 3, we display both the fitted predictor function estimates ${\hat{f}}_{k}({x}_{k})$ as solid lines and the discrete set of specific features *x*_*j*_ in our data set as tics along the different *x*_*k*_-axes.

#### Technical details

All partial predictor functions *f*_*k*_(*x*_*k*_) were modeled using component-wise P-spline base-learners^77^ in order to achieve smooth, non-linear effects which can be parameterized as a weighted sum of basis functions

7 $${f}_{k}(x)=\sum _{b}\,{\beta }_{kb}\cdot {B}_{kb}(x),$$

with cubic B-spline basis functions *B*_*kb*_(*x*)^78^ and with additional penalties on the regression coefficients *β*_*kb*_ for smoothness^79^ and, where required, for monotonicity^80^. Note that the number of hairpins was modeled as a simple linear effect. COSEM current, average elongation rate, and logarithm of transcript abundance were modeled via smooth, monotonically increasing base-learners^80^.

The functional forms of the estimated effects given by Eq. (7) are varied through the regression coefficients *β*_*kb*_ in order to reduce the squared error loss in an iterative manner. More specifically, the model is fitted using model-based boosting methods with intrinsic variable selection^49,81^. In each iteration, the best-fitting base-learner ${\hat{f}}_{k}$ is selected (i.e., the partial function or estimated effect that explains most of the outcome), and the corresponding regression parameters ${\hat{\beta }}_{kb}$ are updated (see Eq. 7). In the next iteration, the remaining information (=residuals) is computed and used as the outcome to be predicted. Again, the best-fitting base-learner (of all base-learners) is determined and updated. This is repeated until the optimal model is reached. The optimal model, i.e., the optimal number of boosting iterations, was selected via 25-fold bootstrapping. Note that each base-learner can be updated multiple times to achieve the optimal fit. On the other hand, if a base-learner is not selected at all, the variable is considered to have no effect on the outcome (in addition to the variables in the model).

#### Predictor variables for protein abundance

It can be expected that for any mRNA sequence *j*, the abundance of the corresponding protein must increase with (i) the abundance of the mRNA sequence *j* and (ii) the corresponding synthesis rate per mRNA as given by the calculated COSEM current *p*_*j*_. Thus, the set of features ***x***_*j*_ to be considered in the predictive model of the protein expression score includes

- the COSEM current *p*_*j*_ [protein/(*s* × mRNA)] (*x*_1*j*_ in Fig. 3); and
- the logarithm of transcript abundance [log_10_ (mRNA)], where mRNA abundance might be substituted by FPKM, i.e., the number of fragments per kilobase of transcript per million mapped reads in an RNA-Seq experiment (*x*_6*j*_ in Fig. 3);
  In addition, we also considered the following features:
- the average elongation rate *ω*_*j*_ [codon/*s*] (*x*_2*j*_ in Fig. 3);
- the bottleneck index [codon/*s*], i.e., the minimum of the average elongation rates in a sliding window of 10 codons^46^ (*x*_3*j*_ in Fig. 3);
- the accuracy ${a}_{j}=\mathop{\prod }\limits_{i}^{{n}_{j}}\,{a}_{ij}$ for a sequence *j* of *n*_*j*_ codons with codon-specific accuracies *a*_*ij*_ (*x*_4*j*_ in Fig. 3);
- the 5′ mRNA folding energy [kcal/mol]^47,82^ (*x*_5*j*_ in Fig. 3);
- the GC3 content, i.e., the overall GC content measured as the fraction of guanine and cytosine in the third nucleotide positions of all codons (*x*_7*j*_ in Fig. 3);
- the ramp index [codon/*s*], i.e., the average elongation rate in the first 30 codons^45^ (*x*_8*j*_ considered but not selected);
- the number of hairpins in the mRNA structure^47^ (*x*_9*j*_ considered but not selected);
- and mRNA sequence length^15–17^ (*x*_10*j*_ considered but not selected).

For those features that are not dimensionless the units of the quantities in the training data set have been provided in brackets which have to be considered for prediction. Note that all listed sequence features have been considered alike as predictor variables in the full model’s fitting procedure as described above. However, not all sequence features were selected by the boosting algorithm to improve the correlation between protein expression score $\hat{f}$ and measured protein abundances as shown in Fig. 3 and Figs S9–S11. This indicates that the features ramp index, number of hairpins and mRNA sequence length did not improve the prediction of the model in addition to the predictor variables selected in the model for the considered organisms (i.e. COSEM current, (logarithmic) transcript abundance, average elongation rate, bottleneck index, accuracy, 5′ mRNA folding energy and GC3 content). A reduced model that considers per se only the predictor variables COSEM current and (logarithmic) abundance is shown in Figs S15–S17, and a further reduced model only considering (logarithmic) transcript levels is shown in Fig. S18.

#### Implementation and validation of the statistical model

The outlined model-based boosting methods are implemented in the R package mboost^48,83,84^ which we have used to fit our model. For details on the underlying algorithm and boosted prediction models we refer to^84,85^.

Correlations seen between sequence features (cf. Supplementary Figs S12–S14) can result in ambiguities in the selection process. While these ambiguities do not affect the prediction accuracy of the method per se, a choice of features with smaller pairwise correlations may be favorable due to less redundant input. While there are some expected correlations as seen between COSEM current and average elongation rate, there are also correlations between mRNA levels and sequence related features reflecting the observation that an mRNA sequence may contain information about its abundance^22^.

To finally validate our model and make the prediction accuracy comparable, we computed the explained variance *R*^2^ on a separate test data set (30% of the full data set). For our model organisms, transcript and protein abundance data for *E. coli*, *S. cerevisiae*, and HEK293 cells were retrieved from public databases (cf.^50^ and detailed listing in Supplementary Table S17). All in all, we assembled 1563 coding sequences with non-zero transcript and protein abundance for *E. coli*, 4479 for *S. cerevisiae*, and 2136 for HEK293 cells (as of July 21st, 2016). Supplementary Figs S9–S11 show all selected features with their respective contributions to the protein expression score for *E. coli*, *S. cerevisiae*, and HEK293 cells. Despite the flexibility of the base-learners, it turned out that for these organisms most function estimates ${\hat{f}}_{k}$ can be approximated by linear functions within relevant ranges of the sequence feature values.

### Optimizing mRNA sequences

Equation (5) assigns a protein expression score to each mRNA based on its sequence features. Therefore, we can use Eq. (5) to select from a set of *synonymous* mRNAs that one with features that maximize the protein expression score. By making this selection, we derive a sequence that is optimized for maximal expression of the encoded protein.

However, maximal protein output is not always the main target of mRNA optimization. In addition, some features (like transcript abundance) are usually not determined *a priori* for synthetic sequences and, consequently, must be ignored by the optimization algorithm. Therefore, we need to define a generalized, more flexible target function ${\hat{f}}_{v}({{\bf{x}}}_{j})$. This flexible target function allows to weight, i.e., emphasize or ignore, specific features (for example transcript abundance) in the sequence optimization procedure in a user-defined way. This generalization is achieved by introducing weights *v*_*k*_ for the individual function estimates, such that the weighted scoring function becomes

8 $${\hat{f}}_{v}({{\bf{x}}}_{j})=\mathop{\sum }\limits_{k=1}^{K}\,{v}_{k}{\hat{f}}_{k}({x}_{kj}).$$

Note that the weights *v*_*k*_ are no regression coefficients but introduce a user-defined weighting of function estimates ${\hat{f}}_{k}$ which corresponds to a rescaling of the regression coefficients ${\hat{\beta }}_{kb}$ between function estimates ${\hat{f}}_{k}$. In particular, to optimize the expression of the genes *ova* and *manA*, we set the weights of all features *v*_*k*_ = 1 for *k* ≠ 6 and the weight of transcript abundance *v*_6_ = 0.

#### Sequence proposal

At each codon position *i* in the sequence *j*, synonymous codons *l* are proposed with elongation rates *ω*_*ijl*_ and accuracies *a*_*ijl*_. The first test sequence *j* is generated by choosing at each position *i* in the sequence the codon with maximal elongation rate and accuracy assuming that this locally optimal sequence is close to the globally optimal sequence (defined as maximizing the protein expression score in Eq. (8)). In further sequence proposals, a codon *l* is selected among *m*_*ij*_ possible synonymous codons at position *i* in sequence *j* with the proposal probability *π*_*ijl*_

9 $${\pi }_{ijl}\equiv \frac{1}{{W}_{ij}}({s}_{1}\frac{{\omega }_{ijl}-{\omega }_{ij{\min }}}{{\omega }_{ij{\max }}-{\omega }_{ij{\min }}}+{s}_{2}\frac{{a}_{ijl}-{a}_{ij{\min }}}{{a}_{ij{\max }}-{a}_{ij{\min }}}+\varepsilon ),$$

where

$${W}_{ij}\equiv \mathop{\sum }\limits_{l=1}^{{m}_{ij}}\,{s}_{1}\frac{{\omega }_{ijl}-{\omega }_{ijmin}}{{\omega }_{ijmax}-{\omega }_{ijmin}}+{s}_{2}\frac{{a}_{ijl}-{a}_{ijmin}}{{a}_{ijmax}-{a}_{ijmin}}+\varepsilon .$$

The factors *s*_1_ and *s*_2_ can be any non-negative numbers and allow for weighting of codon elongation rates versus accuracies in the codon proposals (default values are *s*_1_ = *s*_2_ = 1 to give equal weight to elongation rates and accuracies in codon proposals), and *ε* = 0.05 is a regularization term which represents the proposal probability of a codon with minimal accuracy and elongation rate.

#### Sequence selection

For each proposed sequence *j*, the sequence features as contained in the (weighted) protein expression score defined through Eq. (8) are evaluated and the (weighted) protein expression score is determined. Both the sequence and its score are kept for further reference if the score exceeds earlier achieved scores. The optimization terminates as soon as the coefficient of variation among the last *m* highest (weighted) protein expression scores falls below 5%. We have chosen *m* = 100 as our simulations showed that this allows for a robust estimate of the coefficient of variation.

### Bacterial strains, plasmids and oligonucleotides

*Salmonella enterica* serovar Typhimurium strain SL7207 (Δ*hisG*, ΔaroA) is an attenuated derivative of the wildtype isolate SL1344 with an auxotrophy for aromatic amino acids^86^. Originally, this strain was generously provided by Bruce Stocker. Strain SL7207 Δ*araBAD* was derived from the original strain^87^. Strain SL7207 Δ*araBAD* Δ*manA* (SL-361) was constructed in this work by *λ*-Red recombinase-mediated deletion of *manA*. *E. coli* strain NEB5 *α* (New England Biolabs) was used for general cloning purposes.

Oligonucleotides used in this work are listed in Supplementary Table S18. Plasmid pKD4 was used as DNA template for amplification of the linear DNA fragment for depletion of *manA* from strain SL7207 and pKD46 was used for the temporal expression of *λ*-Red recombinase^88^.

Codon-adapted *manA* and *ova* variants were synthesized, sequenced, and subcloned by Geneart/Life Technologies (cf. Supplementary Table S19). Wildtype (wt) *manA* was amplified from genomic DNA of strain SL7207 using oligos oJT7 and oJT8, subcloned and subsequently sequenced. *manA* -expression plasmids pJT6-pJT9, pJT27–29, and pJT36–39 contain variants of *manA* (wt *manA*, *manA* 1–10) under control of its own promoter (69 bp upstream of the start ATG) in the background of plasmid pETcoco1 (Novagen).

These plasmids were generated by insertion of *manA*/promoter fragments into plasmid pETcoco-1 via Hpal and Swal restriction sites. pETcoco Δ is a relegation product of the empty vector fragment lacking *lacl* of the original plasmid. Ova-expression plasmids pJT20–23 contain variants of the hen egg ovalbumin encoding ova under control of the constitutive *E. coli β*-lactamase promoter in a low copy plasmid background maintained at approximately 15 copies per cell.

Wildtype *ova* was originally amplified with primers from plasmid pOV230^89^ then sequenced and subcloned into plasmid pHL49^90^, yielding plasmid pLK2. From this plasmid wt- *ova* was replaced by codon-adapted variant genes (*ova opt*, *ova1-3*, Supplementary Table S19) via flanking NdeI and HindIII restriction sites. pETcoco-1 and pHL49 derived plasmids harbor *cam*, which encodes the chloramphenicol resistance gene.

### Bacterial growth

*E. coli* and *S. Typhimurium* were routinely grown in liquid LB medium or on LB agar plates. Derivatives of strain SL72077 Δ*ara*Δ*manA* were also grown in M9 minimal medium (MM) supplemented with aro-supplements (40 μg ml^−1^ mannose 40 μg ml^−1^ phenylalanine, 40 μg ml^−1^ tryptophane, 40 μg ml^−1^ tyrosine, 10 μg ml^−1^ 4-aminobenzoic acid, 10 μg ml^−1^ 2,3-dihydroxy-benzoate), 200 μg ml^−1^ mannose and/or 200 mg ml^−1^ glucose. Other supplements were added to media when appropriate, such as 100 μg ml^−1^ ampicilin, 30 μg ml^−1^ streptomycin, 20 μg ml^−1^ chloramphenicol, or 2 mg ml^−1^ L-arabinose. LB medium base and supplements were purchased from Carl Roth, MM base from Sigma-Aldrich. Bacterial growth was monitored in 200 μl cultures at 37 °C and agitation at 700 rpm in a Thermostar microplate incubator (BMG LabTech). 25 ml flask cultures were grown at 37 °C and agitation at 200 rpm Innova 42R incubator (New Brunswick). Optical density was measured at 600 nm (*OD*_600nm_) and the number of colony forming units (cfu) was determined by plating serial dilutions of bacterial cultures on LB-agar plates.

### *λ*-Red recombinase-mediated gene deletion

*λ*-Red recombinase-mediated depletion of *manA* from strain SL7207 Δ*araBAD* was carried out as previously described^87,88^. Briefly, a PCR product harbouring ≈40 bp end sequences homologous to *manA* and a kanamycin resistance marker was amplified with pKD4 as template and primers oJT1 and oJT2. This product was transformed into strain SL7207 Δ*araBAD* harbouring the *λ*-Red recombinase expression plasmid pKD46, and subsequently clones were selected on media plates containing kanamycin and streptomycin. A clone lacking *manA* (SL7207 Δ*ara*Δ*mana*) was identified by colony PCR with primers oJT4 and oHL20.

### Soluble protein extracts

Bacteria were cultured up to an *OD*_600_ ≈ 1 in supplemented MM. 4 × 10^9^ bacteria were harvested at 5 × 10^3^ × g for 5 min. Pellets were washed once, centrifuged again, and then resuspended in 460 ml ice-cold water. The suspension was transferred into glass bead containing tubes (VK01, Precellys) and those were then placed into a Precellys 24 homogenizer for bacterial lysis at 6500 rpm for 20 s with three repetitions. Lysates were centrifuged at 12000 × g for 5 min in a cooled centrifuge and supernatants were stored at −70 °C until further analysis.

### Quantification of ManA expression in *S*. Typhimurium by Immunoblot and multiple reaction monitoring (MRM)

Bacterial lysates were separated with NuPAGE 4% to 12% Bis-Tris gels in an XCell SureLock electrophoresis chamber according to manufacturer’s instructions (ThermoFisher). Samples were prepared using 4 × NuPAGE LDS sample buffer and 10 × NUPAGE reducing agent (ThermoFisher). Page Ruler Plus Marker (ThermoFisher) was used for molecular weight determination of proteins and Roti-Blue reagent (Carl Roth) for unspecific staining of protein bands in gels. Proteins were immobilized on a nitrocellulose membrane (Protan BA79, VWR) using a Semi-Dry-Blotter device (Preqlab). Specific bands were revealed with polyclonal rabbit sera raised against Ova (Acris, R1101) or ManA (MyBioSource, MBS1491170) and subsequent binding of an horseradish peroxidase conjugated antibody (GE, NA934). Roti-Lumin plus spray (Carl Roth) was applied to the membrane and chemoluminescent signals were detected with the Microchemi imager (Biostep) (cf. Supplementary Figs S22 and S23).

Further analysis was performed with ImageJ. Generally, gel images offering the highest contrast below saturation were chosen from images with different exposure times. We used two methods giving identical results, first using a rectangular region of interest (ROI) and measuring median grey intensity for every band, then subtracting the background median grey intensity of every gel; and second with the method outlined in^91^.

ManA levels were also determined by multiple reaction monitoring (MRM). A ManA specific peptide (YDIPELVANVK) was selected using UniProt P25081 as a template and ordered as stable isotope labelled calibration peptide (SpikeTide TQL, JPT, Berlin, Germany). A triple quadrupole mass spectrometer (Xevo TQ-S, Waters) was operated using MRM in positive ionization mode and scanning for 4 specific transitions of the doubly charged natural peptide YIDIPELVANVK (MH^2+^ m/z = 687.82) and the isotopically labelled standard YIDIPELVANVK* (MH^2+^ m/z = 691.39). The quantification was done using the peak area of the transition 687.82 → 869.50 and 691.39 → 877.52, respectively. For further details cf. the Supplementary Information.

As a control, *manA* transcript levels were quantified by qPCR (cf. Supplementary Information).

### Software implementation - OCTOPOS

Two versions of the simulation software were implemented: The Java GUI application OCTOPOS (Optimized Codon Translation fOr PrOtein Synthesis) facilitates easy optimization of sequences using a simpler variant of the scoring function, where function estimates for all features are constrained to linear effects except for the feature GC3 content for which a quadratic approximation was used. The feature weights *v*_*k*_ and proposal weights *s*_1_, *s*_2_ can be adjusted in the software, for details see the software documentation. Secondly, a supplementary C application was developed for fast generation of phase diagrams.

The source code for these programs is available upon request.

## Supplementary information

Supplementary Information
