# Publisher Correction: Optimizing the dynamics of protein expression

**DOI:** 10.1038/s41598-020-62686-5

**Published:** 2020-04-03

**Authors:** Jan-Hendrik Trösemeier, Sophia Rudorf, Holger Loessner, Benjamin Hofner, Andreas Reuter, Thomas Schulenborg, Ina Koch, Isabelle Bekeredjian-Ding, Reinhard Lipowsky, Christel Kamp

**Affiliations:** 1 Division of Microbiology, Paul Ehrlich Institut, Langen, Germany; 2 Max Planck Institute of Colloids and Interfaces, Potsdam-Golm Science Park, Potsdam, Germany; 3 Division of Allergology, Paul Ehrlich Institut, Langen, Germany; 4 Goethe University Frankfurt, Institute of Computer Science, Molecular Bioinformatics, Frankfurt am Main, Germany

Correction to: *Scientific Reports* 10.1038/s41598-019-43857-5, published online 17 May 2019

In the original version of this Article, Jan-Hendrik Trösemeier was incorrectly affiliated with ‘Division of Allergology, Paul Ehrlich Institut, Langen, Germany’. The correct affiliations are listed below.

Division of Microbiology, Paul Ehrlich Institut, Langen, Germany

Max Planck Institute of Colloids and Interfaces, Potsdam-Golm Science Park, Potsdam, Germany

Goethe University Frankfurt, Institute of Computer Science, Molecular Bioinformatics, Frankfurt am Main, Germany

This error has now been corrected in the HTML and PDF versions of this Article.

