# Supplementary material for: Optimizing the dynamics of protein expression
Source: Sci Rep. 2019 May 17;9:7511. doi: 10.1038/s41598-019-43857-5 (PMC6525252; doi:10.1038/s41598-019-43857-5)
Supplement: Supplementary file 1 — Supplementary Information [file 41598_2019_43857_MOESM1_ESM.pdf]

Supplementary Information for "Optimizing the dynamics of protein expression"

Jan-Hendrik Trösemeier[1,2,3,*], Sophia Rudorf[2,*], Holger Loessner[1], Benjamin Hofner[1], Andreas Reuter[1], Thomas Schulenborg[1], Ina Koch[3], Isabelle Bekeredjian-Ding[1], Reinhard Lipowsky[2] Christel Kamp[1,**]
[1]Paul-Ehrlich-Insitut, Langen, Germany
[2] Max Planck Institute of Colloids and Interfaces, Golm, Germany
[3] Goethe University Frankfurt, Institute of Computer Science, Germany
[*] joint first authors
[**] E-mail: christel.kamp@pei.de

Contents

1 Model configuration **6**

2 Model parameters **8**
2.1 Codon-specific elongation rates and accuracies 8
2.2 Drop-off and initiation rates 23
2.3 Sequence features and function estimates in the protein expression score 27

3 Computational and experimental studies with *ova* and *manA* variants **35**

4 Experimental protocols **42**
4.1 Quantification of ManA expression in *S.* Typhimurium lysates by multiple reaction monitoring (MRM) 42
4.2 qPCR for quantification of *manA* transcripts 43

5 Data retrieval and studied gene sequences **44**

6 Optimized Codon Translation fOr PrOtein Synthesis - OCTOPOS **61**

List of Figures

S1 **(Related to Figure 2) Dynamic regimes of mRNA translation for variable drop-off rates.** Ribosomal density of a gene with 300 codons and an elongation rate of $60.32\,\mathrm{s}^{-1}$ as a function of relative initiation rate $\bar{\alpha} = \frac{\alpha}{\omega}$ and relative elongation rate of the last codon $\bar{\beta} = \frac{\beta}{\omega}$ (exit rate) for different drop-off rates per second γ shown at the top of each panel. Generally, if the initiation rate increases or the rate of the last codon (or a bottleneck codon) decreases, the ribosome density will go up. There are various phase boundaries dividing phases of low ribosomal density (LD, upper left, $\bar{\beta}/\bar{\alpha} > 1$), high density (HD, lower right, $\bar{\beta}/\bar{\alpha} < 1$). Ribosome density is reduced in the high density phase by a non-zero drop-off rate. For sufficiently large $\bar{\alpha}$ and $\bar{\beta}$ the system reaches the maximum current phase (MC). The transition point can be approximated by $\bar{\alpha} = \bar{\beta} = \frac{1}{\sqrt{d}+1} \approx 0.24$ for a ribosomal footprint $d = 10$ and $\gamma = 0$ (1). This transition point is shown as vertical black line. 6

S2 **Ratio of simulated translation time over estimated translation time for the wildtype *manA* sequence depending on initiation rates.** The estimated translation time is the sum of codon-dependent translation times, or the harmonic sum of the codon-dependent rates of a coding sequence. The simulated translation time, i.e. the time spent by ribosomes translating in the simulations, is generally larger than the estimated time since ribosome jamming is taken into account. Mean translation times from 1000 simulations with an *E. coli* drop-off rate of $6.6 \times 10^{-3}\,\mathrm{s}^{-1}$ depending on initiation rates per second are shown by triangles. 7

S3 **Elongation rate and error frequencies for *E. coli* at a specific growth rate of $2.5\,\mathrm{h}^{-1}$.** In most cases an increase in elongation rate correlates with a decrease in error frequency. 20

S4 **Elongation rate and error frequencies for *S. cerevisiae*.** In most cases an increase in elongation rate correlates with a decrease in error frequency. 21

S5 **Elongation rate and error frequencies for HEK293.** In most cases an increase in elongation rate correlates with a decrease in error frequency. 22

S6 **Correlation of COSEM current with log-protein abundance in *E. coli* genes for different initiation rates.** Since there are little reliable estimates for initiation rates, an alternative is to choose the initiation rate that maximizes the correlation of COSEM current with log-protein abundance to enhance predictability. The simulations were run with the estimated drop-off rate $6.6 \times 10^{-3}\,s^{-1}$ and hence, if the initiation rate is lower than the drop-off rate little correlation is expected. For *E. coli* a high initiation rate gives the highest correlation. 24
S7 **Correlation of COSEM current with log-protein abundance in *S. cerevisiae* genes for different initiation rates.** Since there are little reliable estimates for initiation rates, an alternative is to choose the initiation rate that maximizes the correlation of COSEM current with log-protein abundance to enhance predictability. The simulations were run with the estimated drop-off rate $9.9 \times 10^{-3}\,s^{-1}$ and hence, if the initiation rate is lower than the drop-off rate little correlation is expected. 25
S8 **Correlation of COSEM current with log-protein abundance in HEK293 genes for different initiation rates.** Since there are little reliable estimates for initiation rates, an alternative is to choose the initiation rate that maximizes the correlation of COSEM current with log-protein abundance to enhance predictability. The simulations were run with the estimated drop-off rate $1.8 \times 10^{-3}\,s^{-1}$ and hence, if the initiation rate is lower then the drop-off rate little correlation is expected. 26
S9 **Function estimates representing features contributing to the protein expression score of *E. coli*.** To estimate protein abundance a generalized additive model with model-based boosting was trained on 70 % of the data set. Monotonicity constraints were used for the COSEM current, average elongation rate and log-transcript abundance. Shown are only sequence features that were selected by the boosting algorithm to improve the prediction of the protein expression score. Generally, the fitted function estimates follow intuition: An increase in protein per time or COSEM current, average elongation rate, bottleneck index (less bottlenecks), accuracy, transcript abundance and folding energy (weaker folding) all contribute to higher expression whereas a balanced GC3 content appears favourable. . . . 27
S10 **Function estimates representing features contributing to the protein expression score of *S. cerevisiae*** as discussed in S9 28
S11 **Function estimates representing features contributing to the protein expression score of HEK293** as discussed in S9 29
S12 **Correlations in sequence features considered in the protein expression score of *E. coli*.** Measured logarithmic protein abundance and sequence features of *E. coli* genes were plotted pairwise with their Pearson correlation coefficients shown in the upper part of the plot matrix. Note that several features have not been selected by the boosting algorithm to contribute to the protein expression score (ramp index, sequence length, number of hairpins). 30
S13 **Correlations in sequence features considered in the protein expression score of *S. cerevisiae*.** Measured logarithmic protein abundance and sequence features of *S. cerevisiae* genes were plotted pairwise with their Pearson correlation coefficients shown in the upper part of the plot matrix. Note that several features have not been selected by the boosting algorithm to contribute to the protein expression score (ramp index, sequence length, number of hairpins). 31

S14 **Correlations in sequence features considered in the protein expression score of human HEK29393 cell lines.** Measured logarithmic protein abundance and sequence features of *S. cerevisiae* genes were plotted pairwise with their Pearson correlation coefficients shown in the upper part of the plot matrix. Note that several features have not been selected by the boosting algorithm to contribute to the protein expression score (ramp index, sequence length, number of hairpins). 32

S15 **(Related to Figure 4) Function estimates and protein expression score against measured protein abundance for a reduced model of *E. coli.*** Different from the full protein expression score, here only COSEM current and log-protein abundance were used for prediction resulting in a slightly lower but comparable coefficient of determination showing that these two features already allow for good predictions of protein abundance. . 33

S16 **(Related to Figure 4) Function estimates and protein expression score against measured protein abundance for a reduced model of *S. cerevisiae*** following the lines of S15 33

S17 **(Related to Figure 4) Function estimates and protein expression score against measured protein abundance for a reduced model of HEK293** following the lines of S15 33

S18 **(Related to Figure 4) Protein expression score against measured protein abundance for reduced models of *E. coli*, *S. cerevisiae* and HEK293 taking only transcript levels into account.** Different from the full protein expression score and the reduced model presented in Figures S15 to S17, here only transcript levels were used for prediction, again resulting in a lower coefficient of determination. 34

S19 **Harmonic and arithmetic mean of the elongation rates of all *E. coli*, *S. cerevisiae* and HEK293 sequences.** Given a sequence of codons with average individual elongation times t_i the overall elongation time in the absence of ribosome queueing is, on average, $\sum_i t_i$. In terms of rates ω_i, the average overall elongation time is $\sum_i 1/\omega_i$, i.e. proportional to the inverse of the harmonic mean of elongation rates $1/\left(\sum_i 1/\omega_i\right)$. However, both, arithmetic and harmonic mean are highly correlated and can be used as predictors for protein abundance. 34

S20 **Rolling mean of codon adaptation index for all *manA* variants of width 10.** Geometric mean of the first 50 codons' CAI is superimposed as solid line and the geometric mean of the rest of the sequences' CAI as dashed line, the sequences mean CAI is given in each panels title. The CAI is calculated with the codon usage of *Salmonella typhimurium LT2* (2). The wildtype shows an existing ramp with a ratio of 0.83 of average CAI in the first 50 codons compared to the remaining codons. The Accuracy, Intermediate and Speed variants do not distinguish between ramp and non-ramp codons. Thus the ratio is close to 1 $(0.91, 1.04, 0.98)$ in contrast to the variants with ramp and ratios of $0.77, 1.33, 0.63$ in which the Intermediate variant has an average CAI that is lower than that of the wildtype and thus lower than the wildtype-ramp leading to a ratio larger than 1. Analysis of the *manA* mRNA sequence profile with respect to CAI and average elongation rate (cf. Figure S21) led us to the conservative approach of keeping a ramp of 50 codons in *manA* variants instead of 30 codons considered otherwise. 35

S21 **Rolling mean of average elongation rate for all *manA* variants of width 10.** Mean elongation rate of the first 50 codons is superimposed as solid line, mean elongation rate in the rest of the sequence as dashed line, the sequences' mean elongation rate is given in each panels title (in codons per second). The figure is similar to S20 with one notable difference: Whereas the ratio of CAI of the first 50 codons to the remainder for the Geneart variant is 0.98 and hence showing no relevant ramp-effect, there is a more pronounced difference of 0.86 for the average elongation rate. Analysis of the *manA* mRNA sequence profile with respect to CAI (cf. Figure S20) and average elongation rate led us to the conservative approach of keeping a ramp of 50 codons in *manA* variants instead of 30 codons considered otherwise. 36

S22 **Ova specific Western blot.** This blot corresponds to data shown in Figures 5, S24-S26. We prepared extracts of *S.* Typhimurium strains expressing variants of *ova* genes (cf. Figure 5, Table S19), i.e. wildtype (1), optimized with Geneart (2), deoptimized (3), optimized (4), intermediate (5), empty vector (6). We subjected 8 µll of each extract to analysis as described in the methods section. The figure shows uncropped data derived from one gel, the asterisks indicate unspecific bands. 37

S23 **ManA specific Western blot.** This blot corresponds to data shown in Figures 6, S24-S26. We prepared extracts of *S.* Typhimurium strains expressing *manA* genes (cf. Figure 6, Table S19), i.e. empty vector control (1), wildtype (2), optimized with Geneart with slow codons between secondary structures (3), optimized with Geneart (4), optimized with Geneart conserving ramp (5), deoptimized (6), optimized for accuracy (7), optimized for speed (8), intermediate (9), optimized for accuracy conserving ramp (10), optimized for speed with ramp (11), intermediate conserving ramp (12). We subjected 8 µl of each extract to analysis as described in the methods section. The figure shows uncropped data derived from one gel, the asterisks indicates unspecific bands. 38

S24 **Comparison of mass spectrometry and Western blot protein abundance measurements.** The figure shows measured protein abundance relative to wildtype with standard deviations for both methods. The linear fit takes into account the respective uncertainties according to Orear's effective variance method. The two measurement methods show very similar results, i.e. slope and asymptote of the linear fit are 0.93 ± 0.7 and 0.1 ± 0.1 and the Pearson's correlation coefficient is 0.97 with a lower and upper confidence interval of 0.8 to 0.99. 39

S25 **(Related to Figure 7) Measured and predicted protein abundance taking transcript levels into account.** Top Panel: mRNA transcript levels relative to wild type were determined for *manA* variants by quantitative real-time PCR (3 biological replicates and 3 technical replicates each) and were not significantly different from each other within the errors seen in the qPCR measurements. Bottom Panel: Measured protein abundance relative to wildtype compared to protein expression score relative to wild type for *manA* variants. Protein levels are weighted averages of mass spectrometry measurements (3 biological replicates and 3 digestion replicates each) and Western blots (3–5 replicates each) which correlate well (cf. Supplementary Figure S18). Different from Fig. 6 of the main manuscript mRNA levels were considered in the protein expression score. As transcript levels are not significantly different from each other within the large errors seen in the qPCR measurements their consideration in the protein expression score results in shifts and large error bars in the predicted protein levels without adding information. 40

S26 **Protein expression levels relative to wildtype compared to doubling times of *S.* Typhimurium population on minimal mannose medium.** An increase of ManA expression reduces the doubling time and hence increases the growth rate. 41

S27 **OCTOPOS software** 61

List of Tables

S1 **Parameters used to calculate codon-specific elongation rates and error frequencies for *S. cerevisiae* and *HEK293* cells.** . . . 9
S2 ***In-vivo* rates of ribosomal transitions for *S. cerevisiae* and *HEK293* cells.** The *in-vivo* rates of ribosomal transitions (with relative standard deviations RSD) were obtained under the assumption of a 2-1-2 pathway of tRNA release from the ribosomal E site by minimizing the kinetic distance to rates measured *in vitro* for *E. coli*, see (3; 4) for a detailed description and the corresponding parameters in *E. coli*. . . . 9
S3 **Concentrations of tRNAs in *S. cerevisiae*.** . . . 10
S4 **Concentrations of tRNAs in *HEK293* cells.** . . . 11
S5 **Anti-codons of cognate tRNAs and codon usages (CU) for all codons in *S. cerevisiae*.** . . . 12
S6 **Anti-codons of cognate tRNAs and codon usages (CU) for all codons in *HEK293* cells.** . . . 13
S7 **Codon-specific elongation rates for *E. coli* at a specific growth rate of $2.5\,\mathrm{h}^{-1}$ as previously published in (4).** See supplementary data in (4) for codon-specific elongation rates at other specific growth rates. . . . 14
S8 **Codon-specific elongation rates for *S. cerevisiae*.** . . . 15
S9 **Codon-specific elongation rates for HEK293.** . . . 16
S10 **Codon-specific error frequencies for *E. coli* at a specific growth rate of $2.5\,\mathrm{h}^{-1}$.** 17
S11 **Codon-specific error frequencies for *S. cerevisiae*.** . . . 18
S12 **Codon-specific error frequencies for *HEK293* cells.** . . . 19
S13 **Organism specific drop-off rates.** Drop-off rates were evaluated assuming drop-off probabilities per codon of 3×10^{-4}. . . . 23
S14 **Initiation rates maximizing the correlation between COSEM current and protein abundance.** Plausible ranges are suggested as there are not always clear optima as can be seen in supplementary Figs. S6 to S8 . . . 23
S15 **Final concentrations of the calibration peptide.** . . . 42
S16 **MRM Transitions and collision energy.** . . . 43
S17 **Data sources for protein expression and mRNA transcript levels**. Data sets for protein abundance from PaxDb (5) are provided in the database's common abundance metric in parts per million (ppm) for the human HEK293 cell line, *E. coli* and *S. cerevisiae*. Transcript abundance was estimated from RNA-seq experiments in which FPKM (number of Fragments Per Kilobase of transcript per Million mapped reads in an RNA-Seq experiment) was taken as a proxy for transcript abundance. For model based boosting log-transformed data were used. Latest date of retrieval was 2016/07/21. . . . 44
S18 Ologinucleotides used. . . . 44
S19 **List of all sequences used.** The table lists internal ids for documentation purposes (plasmid, name, gid, DNA, GK), alternate long names and coding sequences. . . . 45

1 Model configuration

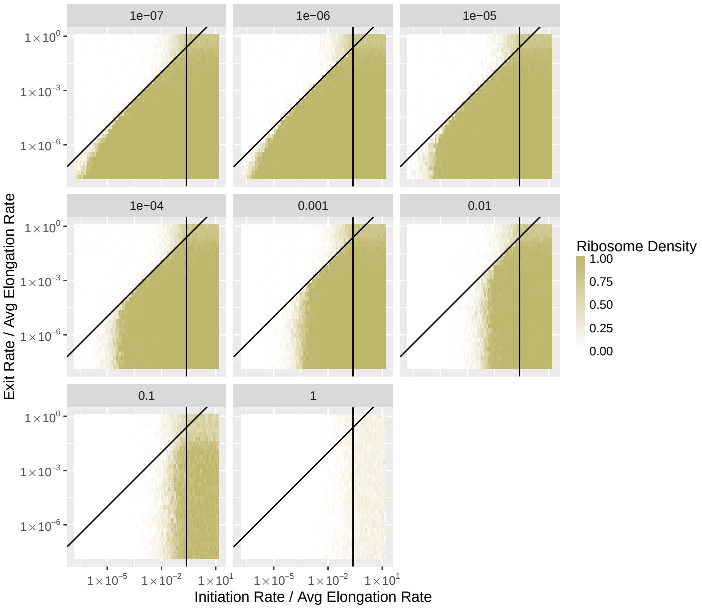

Figure S1. (Related to Figure 2) Dynamic regimes of mRNA translation for variable drop-off rates. Ribosomal density of a gene with 300 codons and an elongation rate of $60.32\,\mathrm{s}^{-1}$ as a function of relative initiation rate $\bar{\alpha} = \frac{\alpha}{\omega}$ and relative elongation rate of the last codon $\bar{\beta} = \frac{\beta}{\omega}$ (exit rate) for different drop-off rates per second γ shown at the top of each panel. Generally, if the initiation rate increases or the rate of the last codon (or a bottleneck codon) decreases, the ribosome density will go up. There are various phase boundaries dividing phases of low ribosomal density (LD, upper left, $\bar{\beta}/\bar{\alpha} > 1$), high density (HD, lower right, $\bar{\beta}/\bar{\alpha} < 1$). Ribosome density is reduced in the high density phase by a non-zero drop-off rate. For sufficiently large $\bar{\alpha}$ and $\bar{\beta}$ the system reaches the maximum current phase (MC). The transition point can be approximated by $\bar{\alpha} = \bar{\beta} = \frac{1}{\sqrt{d}+1} \approx 0.24$ for a ribosomal footprint $d = 10$ and $\gamma = 0$ (1). This transition point is shown as vertical black line.

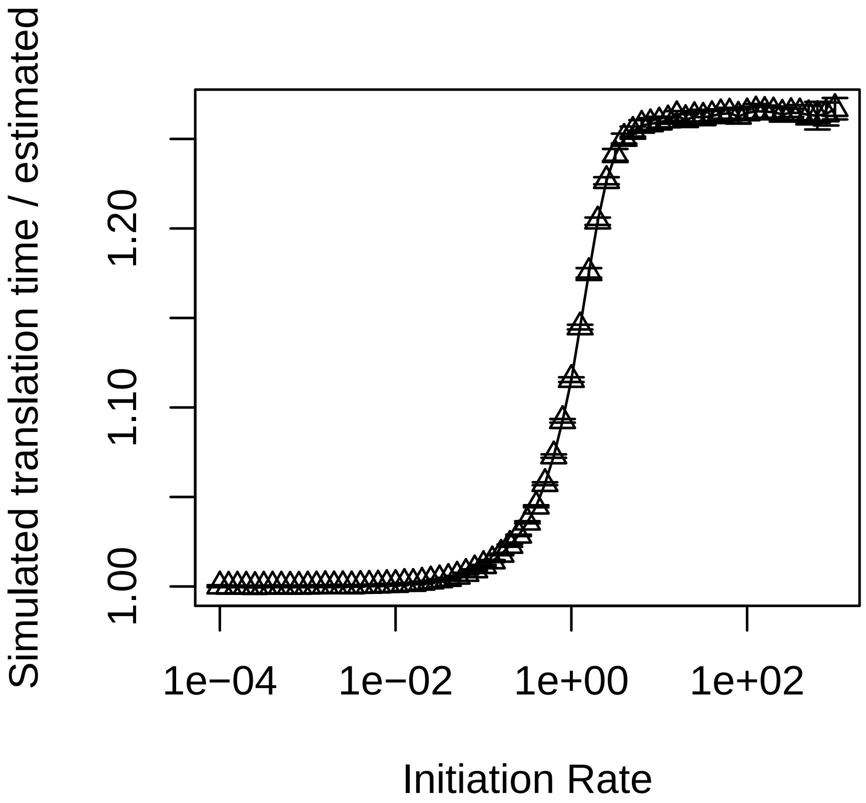

Figure S2. Ratio of simulated translation time over estimated translation time for the wildtype *manA* sequence depending on initiation rates. The estimated translation time is the sum of codon-dependent translation times, or the harmonic sum of the codon-dependent rates of a coding sequence. The simulated translation time, i.e. the time spent by ribosomes translating in the simulations, is generally larger than the estimated time since ribosome jamming is taken into account. Mean translation times from 1000 simulations with an *E. coli* drop-off rate of $6.6 \times 10^{-3}\,s^{-1}$ depending on initiation rates per second are shown by triangles.

2 Model parameters

2.1 Codon-specific elongation rates and accuracies

Definitions The **codon-specific elongation rate** $\omega_{j,i}$ for codon i in sequence j is defined as the inverse of the time that it takes a ribosome in average to translate that codon.
The **codon-specific accuracy** $a_{j,i}$ for codon i in sequence j is defined as the probability for a ribosome to incorporate a tRNA that is cognate (i.e., not near-cognate nor non-cognate) to codon i when translating codon i.

Derivation Codon-specific elongation rates $\omega_{j,i}$ and accuracies $a_{j,i}$ have been derived for *E. coli*, *S. cerevisiae* and human *HEK293* cells as was previously explained in detail (4). Briefly, we describe translation elongation as a Markov process with 12 states per codon, which represent the following steps:
Initial binding of a non-cognate, near-cognate, or cognate ternary complex (EF-Tu·GTP·aa-tRNA) to the ribosome is followed by either dissociation or further movement into the A site of the ribosome (recognition). The recognition of a cognate ternary complex is followed by GTPase activation and GTP hydrolysis with subsequent phosphate release and conformational rearrangements of EF-Tu. A cognate ternary complex is then usually fully accommodated in the ribosomal A site, or with small probability, is released from the A site. Near-cognate ternary complexes are usually rejected by the ribosome. With low probability, recognition of a near-cognate ternary complex leads to GTPase activation and GTP hydrolysis, after which the near-cognate ternary complex is most likely released or – again with low probability – gets fully accommodated in the ribosomal A site. Finally, after complete accommodation of a cognate or near-cognate tRNA in the A site, a new peptide bond is formed and the ribosome/tRNA complex undergoes translocation to the next codon on the mRNA.
Transitions between the different states of the Markov model occur with specific transition rates, for which values were obtained as introduced in (4). We assume that all transition rates are codon-independent, except for the binding rates of cognate, near-cognate, and non-cognate ternary complexes. These rates are taken to be proportional to the concentrations of free cognate, near-cognate, and non-cognate ternary complexes, respectively.

Codon-specific elongation rates $\omega_{j,i}$ and accuracies $a_{j,i}$ are calculated from the Markov model's structure and transition rates by standard methods for Markov processes. In particular, elongation rates and accuracies explicitly depend on the rates of cognate, near-cognate, and non-cognate ternary complexes binding to the ribosome and, thus, on the concentrations of free cognate, near-cognate, and non-cognate ternary complexes, respectively. Exact expressions for the codon-specific elongation rates and accuracies are given in (4). All parameters needed to compute the codon-specific elongation rates and accuracies are listed in Tables S1 to S6 or assume values as given in (4). The results are listed in Tables S7 to S12. See also supplementary data in (4) for codon-specific elongation rates in *E. coli* at different specific growth rates.

Table S1. Parameters used to calculate codon-specific elongation rates and error frequencies for *S. cerevisiae* and *HEK293* cells.

Parameter	Organism	Value	Reference
Ribosomal transition rates	both	Table S2	this document
Definition of “cognate”	*S. cerevisiae*	Table S3	(6) (suppl. data)
	HEK293	Table S4	(7) (Fig. 34)
Definition of “near-cognate”	both		according to (8; 3)
Codon usages	*S. cerevisiae*	Table S5	from mRNA abundances (9) (suppl. data)
	HEK293	Table S6	from mRNA abundances (10) (suppl. data)
tRNAs per cell	*S. cerevisiae*	Table S3	(6)
	HEK293	Table S4	(11)
Ribosomes per cell	*S. cerevisiae*	2×10^5	(9)
	HEK293	1×10^7	(12)
eEF1a1 per cell	*S. cerevisiae*	2×10^6	(13)
	HEK293	1×10^8	(12)
Cell volume	*S. cerevisiae*	$37\,\mu m^3$	(14)
	HEK293	$1150\,\mu m^3$	(15)
Average elongation rate	*S. cerevisiae*	$33\,s^{-1}$	(9)
	HEK293	$6\,s^{-1}$	(16)

Table S2. *In-vivo* rates of ribosomal transitions for *S. cerevisiae* and *HEK293* cells. The *in-vivo* rates of ribosomal transitions (with relative standard deviations RSD) were obtained under the assumption of a 2-1-2 pathway of tRNA release from the ribosomal E site by minimizing the kinetic distance to rates measured *in vitro* for *E. coli*, see (3; 4) for a detailed description and the corresponding parameters in *E. coli*.

Rate	*S. cerevisiae*	*HEK293*	RSD	Unit
$\kappa_{on}^{\star}$	94		0.1	$\mu M^{-1}\,s^{-1}$
$\omega_{off}^{\star}$	6400	1800	0.4	s^{-1}
$\omega_{rec}^{\star}$	14900	2900	0.3	s^{-1}
$\omega_{21}^{\star}$	2	2	0.3	s^{-1}
$\omega_{23}^{\star}$	2300	1500	0.3	s^{-1}
$\omega_{con}^{\star}$	820	470		s^{-1}
$\omega_{45}^{\star}$	750	230	0.2	s^{-1}
$\omega_{40}^{\star}$	1	1		s^{-1}
$\omega_{76}^{\star}$	16700	3200	0.3	s^{-1}
$\omega_{78}^{\star}$	3	5	0.3	s^{-1}
$\omega_{910}^{\star}$	0.28	0.29	0.2	s^{-1}
$\omega_{90}^{\star}$	8	6	0.2	s^{-1}
$\omega_{pro}^{\star}$	450	170	0.5	s^{-1}

Table S3. Concentrations of tRNAs in *S. cerevisiae*.

Anti-Codon	Amino acid	Molecules/cell
UGC	Ala	51437
IGC	Ala	113163
UCU	Arg	113163
CCU	Arg	10289
CCG	Arg	10289
ICG	Arg	61726
GUU	Asn	102875
GUC	Asp	154315
GCA	Cys	41152
UUG	Gln	92589
CUG	Gln	10289
UUC	Glu	144026
CUC	Glu	20574
UCC	Gly	30863
CCC	Gly	20574
GCC	Gly	164601
GUG	His	72012
UAU	Ile	20574
IAU	Ile	133738
UAG	Leu	30863
GAG	Leu	10289
UAA	Leu	72012
CAA	Leu	102875
UUU	Lys	72012
CUU	Lys	144026
CAU	Met	51437
GAA	Phe	102875
UGG	Pro	102875
IGG	Pro	20574
GCU	Ser	41152
UGA	Ser	30863
CGA	Ser	10289
IGA	Ser	113163
UGU	Thr	41152
CGU	Thr	10289
IGU	Thr	113163
CCA	Trp	61726
GUA	Tyr	82300
UAC	Val	20574
CAC	Val	20574
IAC	Val	144026

Table S4. Concentrations of tRNAs in *HEK293* cells.

Anti-Codon	Amino acid	Molecules/cell
UGC	Ala	916573
AGC	Ala	3384116
CGC	Ala	850415
UCG	Arg	2250540
CCG	Arg	1446617
ACG	Arg	1179105
UCU	Arg	329157
CCU	Arg	1053973
AUU	Asn	0
GUU	Asn	2452966
GUC	Asp	4288905
GCA	Cys	1292794
UUG	Gln	1782775
CUG	Gln	4822637
UUC	Glu	1552467
CUC	Glu	1328953
UCC	Gly	1195112
CCC	Gly	1813654
GCC	Gly	4646638
GUG	His	3156779
UAU	Ile	381136
AAU	Ile	2266860
GAU	Ile	3321
UAG	Leu	470419
UAA	Leu	2049035
CAA	Leu	726362
CAG	Leu	3139571
AAG	Leu	492534
UUU	Lys	3297624
CUU	Lys	2095918
CAU	Met	7409911
GAA	Phe	2426202
UGG	Pro	1273045
AGG	Pro	601330
CGG	Pro	778307
UGA	Ser	2949312
CGA	Ser	436044
AGA	Ser	1339964
GCU	Ser	2392655
UGU	Thr	1461888
CGU	Thr	961206
AGU	Thr	2399438
CCA	Trp	1695475
GUA	Tyr	984731
AUA	Tyr	261
UAC	Val	1960321
CAC	Val	10343136
AAC	Val	5919816

Table S5. Anti-codons of cognate tRNAs and codon usages (CU) for all codons in *S. cerevisiae*.

Codon	Anti-codon	CU [%]	Codon	Anti-codon	CU [%]
AAA	UUU	2.91	GAA	UUC	5.2
AAC	GUU	3.01	GAC	GUC	2.35
AAG	CUU	5.76	GAG	CUC	1.13
AAU	GUU	1.77	GAU	GUC	2.73
ACA	UGU	0.9	GCA	UGC	0.95
ACC	IGU	1.99	GCC	IGC	2.07
ACG	CGU	0.39	GCG	UGC	0.33
ACU	IGU	2.5	GCU	IGC	4.65
AGA	UCU	3.79	GGA	UCC	0.58
AGC	GCU	0.59	GGC	GCC	0.76
AGG	CCU	0.46	GGG	CCC	0.37
AGU	GCU	0.73	GGU	GCC	4.81
AUA	UAU	0.75	GUA	UAC	0.61
AUC	IAU	2.41	GUC	IAC	2.36
AUG	CAU	1.47	GUG	CAC	0.68
AUU	IAU	2.7	GUU	IAC	3.43
CAA	UUG	3.2	UAA	*stop*	
CAC	GUG	1.13	UAC	GUA	2.07
CAG	CUG	0.64	UAG	*stop*	
CAU	GUG	0.86	UAU	GUA	1.07
CCA	UGG	2.64	UCA	UGA	0.94
CCC	IGG	0.35	UCC	IGA	1.91
CCG	UGG	0.23	UCG	CGA	0.4
CCU	IGG	0.87	UCU	IGA	2.87
CGA	ICG	0.1	UGA	*stop*	
CGC	ICG	0.13	UGC	GCA	0.39
CGG	CCG	0.08	UGG	CCA	0.96
CGU	ICG	0.93	UGU	GCA	0.87
CUA	UAG	0.91	UUA	UAA	1.87
CUC	GAG	0.27	UUC	GAA	2.34
CUG	UAG	0.54	UUG	CAA	4.08
CUU	GAG	0.62	UUU	GAA	1.57

Table S6. Anti-codons of cognate tRNAs and codon usages (CU) for all codons in *HEK293* cells.

Codon	Anti-codon	CU [%]	Codon	Anti-codon	CU [%]
AAA	UUU	2.73	GAA	UUC	3.19
AAC	GUU	1.94	GAC	GUC	2.69
AAG	CUU	4.11	GAG	CUC	4.35
AAU	GUU	1.66	GAU	GUC	2.59
ACA	UGU	1.41	GCA	UGC	1.68
ACC	AGU	1.76	GCC	AGC	2.91
ACG	CGU	0.56	GCG	CGC	0.75
ACU	AGU	1.31	GCU	AGC	2.14
AGA	UCU	1.14	GGA	UCC	1.67
AGC	GCU	1.64	GGC	GCC	2.38
AGG	CCU	1.02	GGG	CCC	1.51
AGU	GCU	1.12	GGU	GCC	1.33
AUA	UAU	0.62	GUA	UAC	0.77
AUC	AAU,GAU	2.29	GUC	AAC	1.41
AUG	CAU	2.35	GUG	CAC	2.94
AUU	AAU	1.79	GUU	AAC	1.2
CAA	UUG	1.04	UAA	*stop*	
CAC	GUG	1.32	UAC	GUA	1.49
CAG	CUG	3.45	UAG	*stop*	
CAU	GUG	0.97	UAU	GUA,AUA	1.23
CCA	UGG	1.58	UCA	UGA	1.04
CCC	AGG	1.8	UCC	AGA	1.58
CCG	CGG	0.64	UCG	CGA	0.41
CCU	AGG	1.71	UCU	AGA	1.45
CGA	UCG	0.75	UGA	*stop*	
CGC	ACG	1.17	UGC	GCA	0.9
CGG	CCG	1.28	UGG	CCA	1.02
CGU	ACG	0.62	UGU	GCA	0.78
CUA	UAG	0.65	UUA	UAA	0.67
CUC	AAG	1.7	UUC	GAA	1.86
CUG	CAG	3.68	UUG	CAA	1.26
CUU	AAG	1.29	UUU	GAA	1.68

Table S7. Codon-specific elongation rates for *E. coli* at a specific growth rate of $2.5\,h^{-1}$ as previously published in (4). See supplementary data in (4) for codon-specific elongation rates at other specific growth rates.

Codon	Codon-specific elongation rate [s^{-1}]	Codon	Codon-specific elongation rate [s^{-1}]
AAA	12.28	GAA	63.67
AAC	16.65	GAC	38.98
AAG	12.17	GAG	63.24
AAU	16.43	GAU	37.06
ACA	22.66	GCA	43.12
ACC	16.23	GCC	10.43
ACG	32.91	GCG	42.27
ACU	32.21	GCU	41.95
AGA	15.95	GGA	23.06
AGC	16.75	GGC	55.93
AGG	10.64	GGG	36.68
AGU	16.55	GGU	53.69
AUA	5.53	GUA	48.35
AUC	59.37	GUC	23.09
AUG	10.87	GUG	45.43
AUU	57.40	GUU	56.47
CAA	15.99	UAA	*stop*
CAC	11.85	UAC	33.90
CAG	16.30	UAG	*stop*
CAU	11.83	UAU	33.44
CCA	3.92	UCA	26.18
CCC	14.55	UCC	12.27
CCG	9.68	UCG	30.02
CCU	16.79	UCU	32.65
CGA	62.78	UGA	*stop*
CGC	60.37	UGC	26.44
CGG	9.26	UGG	21.68
CGU	60.30	UGU	26.09
CUA	11.98	UUA	15.52
CUC	18.82	UUC	8.05
CUG	60.32	UUG	44.25
CUU	18.12	UUU	7.60

Table S8. Codon-specific elongation rates for *S. cerevisiae*.

Codon	Codon-specific elongation rate [s^{-1}]	Codon	Codon-specific elongation rate [s^{-1}]
AAA	39.61	GAA	70.86
AAC	46.99	GAC	72.97
AAG	69.91	GAG	10.42
AAU	46.99	GAU	72.97
ACA	31.84	GCA	37.24
ACC	57.40	GCC	41.15
ACG	7.23	GCG	38.63
ACU	57.40	GCU	41.15
AGA	63.49	GGA	25.46
AGC	27.25	GGC	77.24
AGG	6.64	GGG	18.60
AGU	27.25	GGU	77.24
AUA	14.29	GUA	15.74
AUC	64.73	GUC	65.35
AUG	36.11	GUG	15.34
AUU	64.73	GUU	65.35
CAA	52.70	UAA	*stop*
CAC	46.49	UAC	45.87
CAG	4.54	UAG	*stop*
CAU	46.49	UAU	45.87
CCA	62.11	UCA	23.05
CCC	9.34	UCC	56.02
CCG	64.19	UCG	7.23
CCU	9.34	UCU	56.02
CGA	45.98	UGA	*stop*
CGC	46.28	UGC	28.23
CGG	11.05	UGG	49.66
CGU	46.28	UGU	28.23
CUA	17.82	UUA	49.64
CUC	1.79	UUC	52.98
CUG	18.18	UUG	54.89
CUU	1.79	UUU	52.98

Table S9. Codon-specific elongation rates for HEK293.

Codon	Codon-specific elongation rate [s^{-1}]	Codon	Codon-specific elongation rate [s^{-1}]
AAA	21.21984	GAA	9.11984
AAC	14.12447	GAC	21.96470
AAG	11.23207	GAG	5.48026
AAU	14.12483	GAU	21.96470
ACA	10.48064	GCA	5.89915
ACC	14.78216	GCC	17.75464
ACG	7.33020	GCG	6.04496
ACU	14.78260	GCU	17.75464
AGA	1.59880	GGA	8.12605
AGC	15.08409	GGC	24.84194
AGG	7.37762	GGG	11.61666
AGU	15.08453	GGU	24.84194
AUA	2.51558	GUA	13.09163
AUC	11.82971	GUC	28.00956
AUG	34.86228	GUG	41.11098
AUU	11.81255	GUU	28.01011
CAA	12.59770	UAA	171.67736
CAC	18.93839	UAC	5.27462
CAG	27.28044	UAG	171.67736
CAU	18.93835	UAU	5.27632
CCA	8.84760	UCA	21.12999
CCC	1.00958	UCC	7.59376
CCG	5.92273	UCG	3.59940
CCU	1.00958	UCU	7.59376
CGA	17.13684	UGA	171.67736
CGC	7.35781	UGC	8.83135
CGG	10.10466	UGG	13.32441
CGU	7.35781	UGU	8.83135
CUA	3.35796	UUA	15.73559
CUC	0.71702	UUC	14.33256
CUG	16.39336	UUG	4.20173
CUU	0.71704	UUU	14.33295

Table S10. Codon-specific error frequencies for *E. coli* at a specific growth rate of $2.5\,\mathrm{h}^{-1}$.

Codon	Error frequency $[10^{-4}]$	Codon	Error frequency $[10^{-4}]$
AAA	8.96	GAA	1.08
AAC	7.65	GAC	3.18
AAG	9.66	GAG	1.18
AAU	8.37	GAU	4.40
ACA	3.54	GCA	2.49
ACC	6.74	GCC	14.36
ACG	2.17	GCG	2.92
ACU	3.55	GCU	3.08
AGA	6.80	GGA	8.91
AGC	10.90	GGC	1.64
AGG	9.61	GGG	4.06
AGU	11.55	GGU	2.33
AUA	26.51	GUA	1.97
AUC	0.69	GUC	6.99
AUG	18.24	GUG	3.19
AUU	1.22	GUU	1.78
CAA	8.94	UAA	*stop*
CAC	12.61	UAC	1.82
CAG	8.80	UAG	*stop*
CAU	12.77	UAU	2.19
CCA	38.82	UCA	2.37
CCC	6.98	UCC	7.13
CCG	15.52	UCG	3.15
CCU	8.49	UCU	2.79
CGA	0.44	UGA	*stop*
CGC	1.03	UGC	5.12
CGG	18.21	UGG	4.64
CGU	1.05	UGU	5.59
CUA	14.13	UUA	6.11
CUC	9.11	UUC	19.47
CUG	1.04	UUG	2.16
CUU	11.02	UUU	26.22

Table S11. Codon-specific error frequencies for *S. cerevisiae*.

Codon	Error frequency [10^{-4}]	Codon	Error frequency [10^{-4}]
AAA	2.08	GAA	0.69
AAC	2.00	GAC	1.04
AAG	0.40	GAG	8.18
AAU	2.00	GAU	1.04
ACA	2.28	GCA	1.72
ACC	1.02	GCC	2.24
ACG	11.78	GCG	0.97
ACU	1.02	GCU	2.24
AGA	0.56	GGA	3.39
AGC	3.66	GGC	0.76
AGG	11.67	GGG	3.35
AGU	3.66	GGU	0.76
AUA	5.82	GUA	5.04
AUC	0.93	GUC	1.10
AUG	1.68	GUG	4.37
AUU	0.93	GUU	1.10
CAA	1.17	UAA	*stop*
CAC	1.40	UAC	1.55
CAG	17.04	UAG	*stop*
CAU	1.40	UAU	1.55
CCA	0.66	UCA	2.69
CCC	9.20	UCC	0.97
CCG	0.26	UCG	10.94
CCU	9.20	UCU	0.97
CGA	1.14	UGA	*stop*
CGC	1.03	UGC	3.15
CGG	4.95	UGG	0.53
CGU	1.03	UGU	3.15
CUA	3.55	UUA	0.77
CUC	49.82	UUC	1.47
CUG	2.69	UUG	0.84
CUU	49.82	UUU	1.47

Table S12. Codon-specific error frequencies for *HEK293* cells.

Codon	Error frequency [10^{-4}]	Codon	Error frequency [10^{-4}]
AAA	4.88	GAA	19.98
AAC	15.46	GAC	9.73
AAG	22.17	GAG	56.89
AAU	15.46	GAU	9.73
ACA	15.16	GCA	32.46
ACC	10.59	GCC	11.97
ACG	29.21	GCG	41.88
ACU	10.59	GCU	11.97
AGA	109.98	GGA	21.81
AGC	11.34	GGC	7.80
AGG	31.56	GGG	22.57
AGU	11.34	GGU	7.80
AUA	104.34	GUA	19.95
AUC	22.37	GUC	8.50
AUG	4.62	GUG	3.69
AUU	22.41	GUU	8.49
CAA	15.84	UAA	*stop*
CAC	8.77	UAC	35.56
CAG	4.56	UAG	*stop*
CAU	8.77	UAU	35.54
CCA	16.90	UCA	3.94
CCC	179.23	UCC	22.90
CCG	29.98	UCG	38.16
CCU	179.23	UCU	22.90
CGA	5.28	UGA	*stop*
CGC	27.38	UGC	20.19
CGG	20.58	UGG	6.62
CGU	27.38	UGU	20.19
CUA	55.73	UUA	7.06
CUC	343.60	UUC	11.71
CUG	17.06	UUG	81.58
CUU	343.54	UUU	11.71

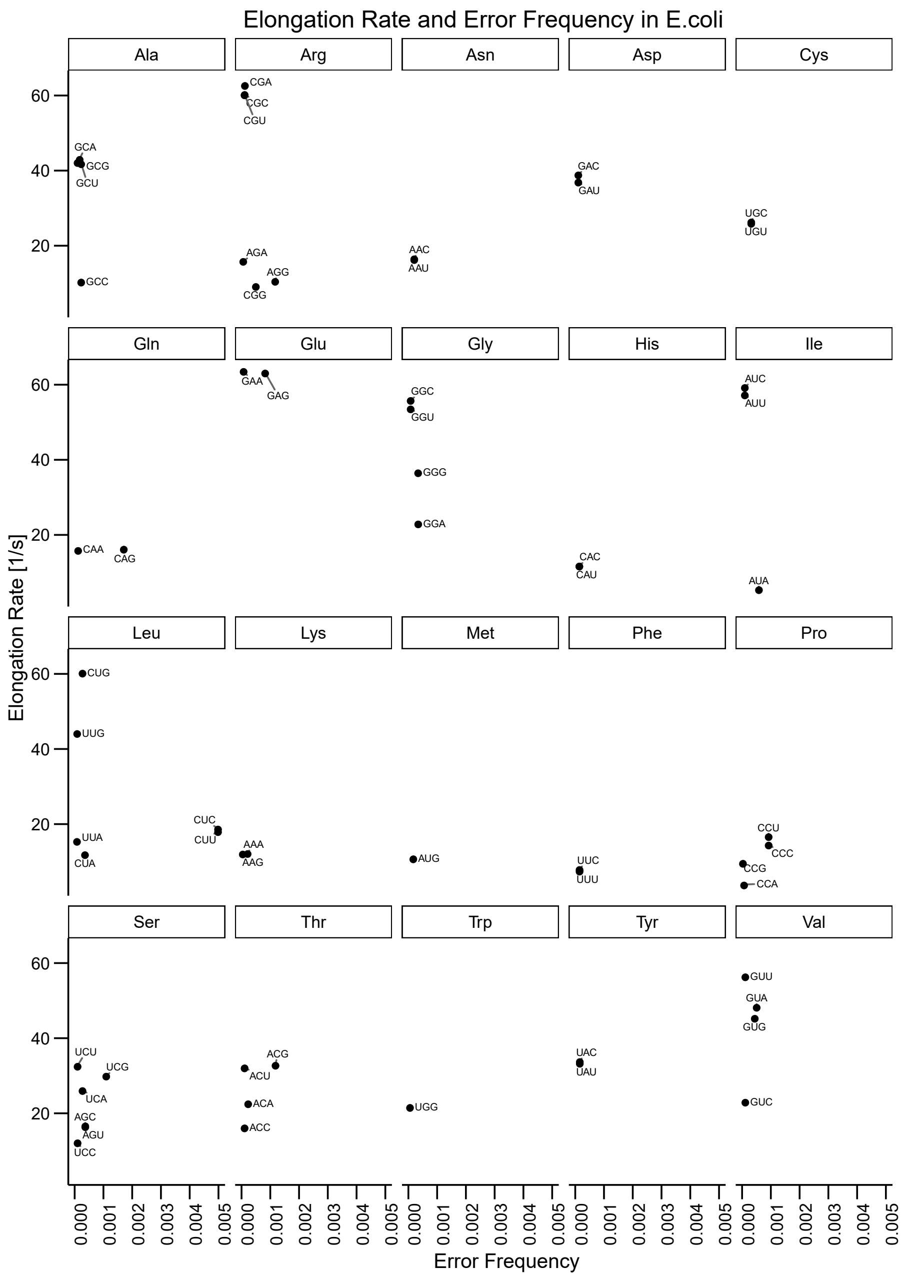

Figure S3. Elongation rate and error frequencies for *E. coli* at a specific growth rate of $2.5\,\mathrm{h}^{-1}$**.** In most cases an increase in elongation rate correlates with a decrease in error frequency.

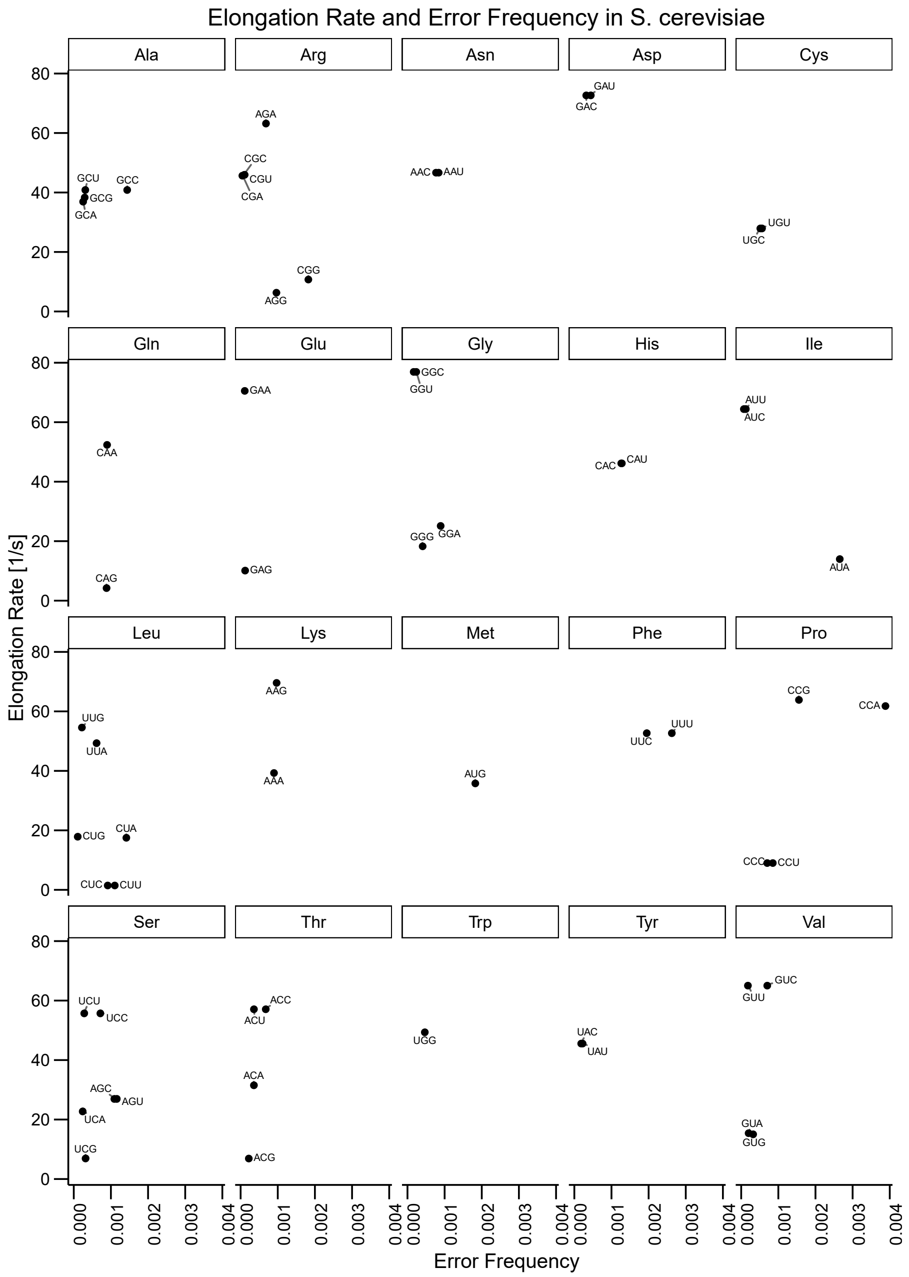

Figure S4. Elongation rate and error frequencies for *S. cerevisiae*. In most cases an increase in elongation rate correlates with a decrease in error frequency.

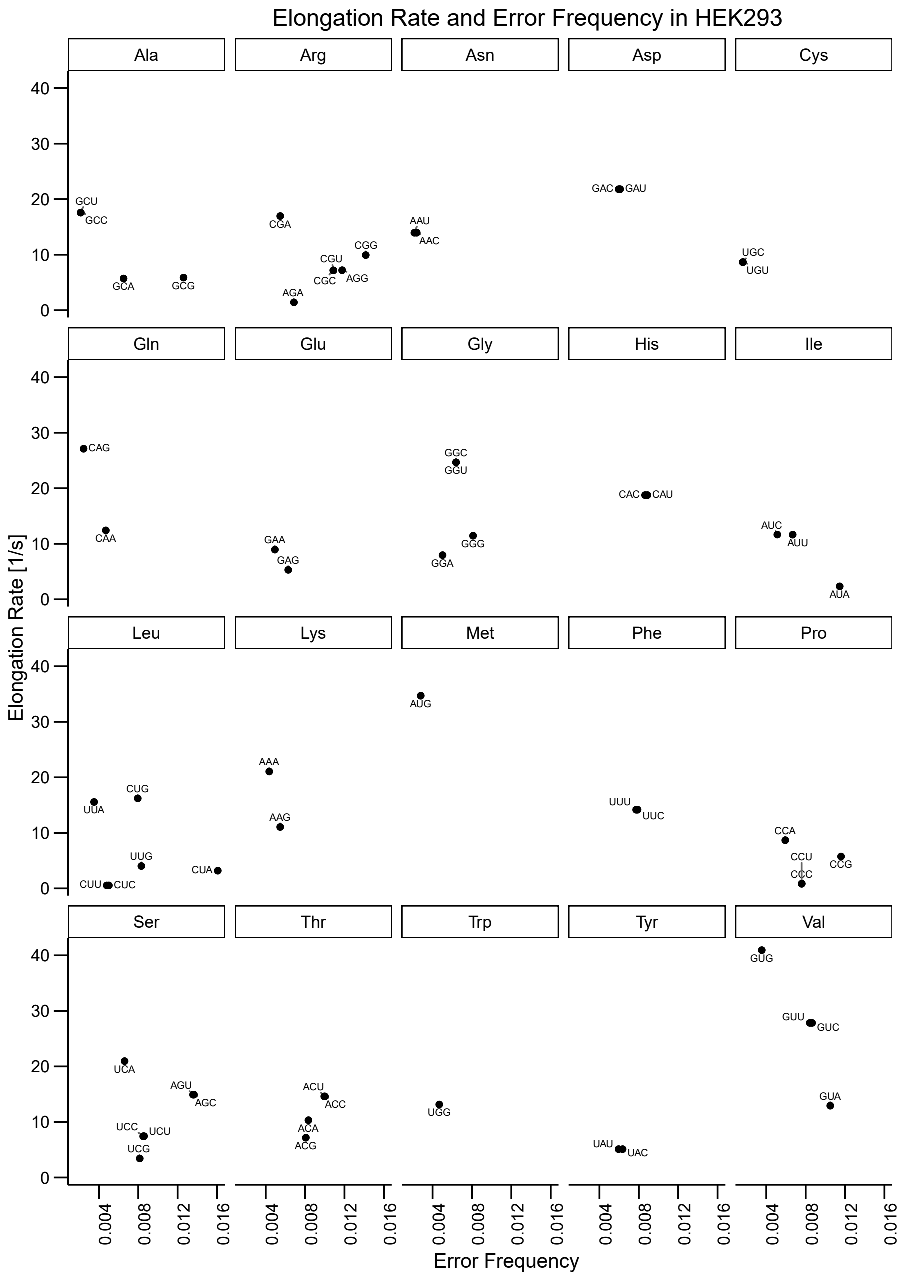

Figure S5. Elongation rate and error frequencies for HEK293. In most cases an increase in elongation rate correlates with a decrease in error frequency.

2.2 Drop-off and initiation rates

Table S13. Organism specific drop-off rates. Drop-off rates were evaluated assuming drop-off probabilities per codon of 3×10^{-4}.

Organism	Drop-off prob. per codon	Reference	Avg. elongation rate	Drop-off rate
E. coli	2.7×10^{-4}	(17)	$22\,s^{-1}$	$6.6 \times 10^{-3}\,s^{-1}$
E. coli	1.4×10^{-4} to 5.6×10^{-4}	(18)		
S. cerevisiae	0×10^{-3} to 2×10^{-3}	(19)	$33\,s^{-1}$	$9.9 \times 10^{-3}\,s^{-1}$
HEK293			$6\,s^{-1}$	$1.8 \times 10^{-3}\,s^{-1}$

Table S14. Initiation rates maximizing the correlation between COSEM current and protein abundance. Plausible ranges are suggested as there are not always clear optima as can be seen in supplementary Figs. S6 to S8

Organism	Optimal [s^{-1}]	Plausible Range [s^{-1}]
E. coli	$100\,s^{-1}$	$> 10\,s^{-1}$
S. cerevisiae	$2.5\,s^{-1}$	$1\,s^{-1}$ to $5\,s^{-1}$
HEK293	$0.06\,s^{-1}$	$> 0.01\,s^{-1}$

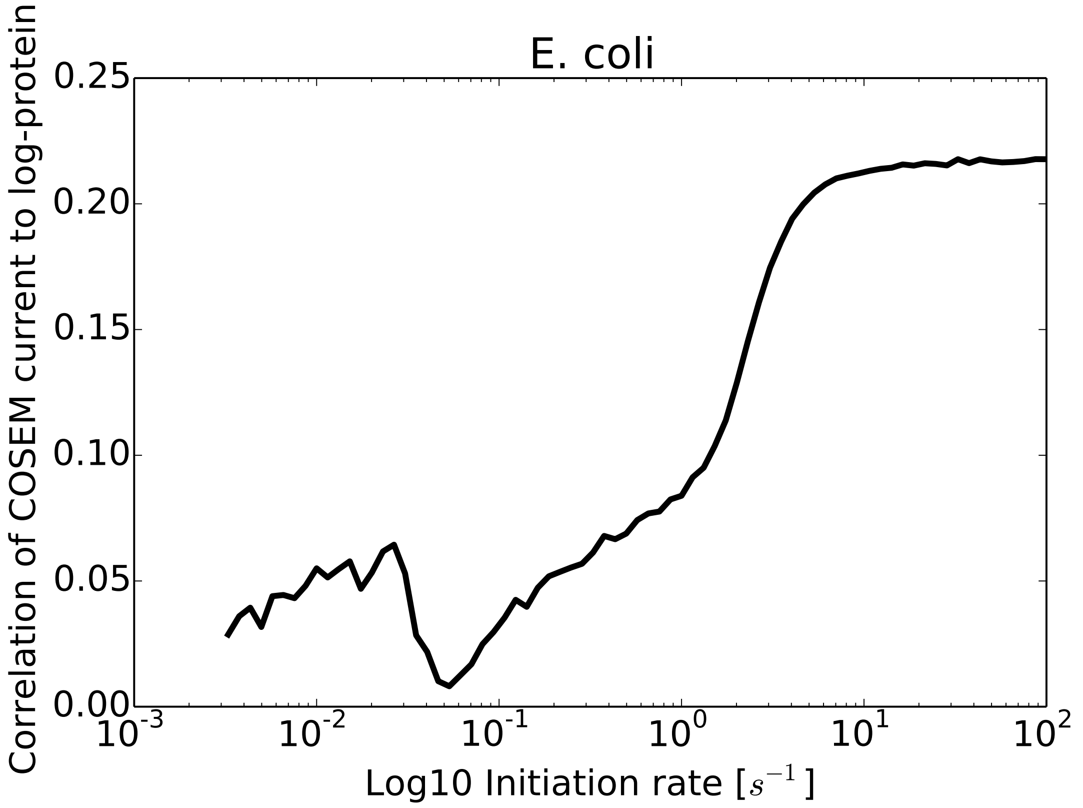

Figure S6. Correlation of COSEM current with log-protein abundance in *E. coli* genes for different initiation rates. Since there are little reliable estimates for initiation rates, an alternative is to choose the initiation rate that maximizes the correlation of COSEM current with log-protein abundance to enhance predictability. The simulations were run with the estimated drop-off rate $6.6 \times 10^{-3}\,\mathrm{s}^{-1}$ and hence, if the initiation rate is lower than the drop-off rate little correlation is expected. For *E. coli* a high initiation rate gives the highest correlation.

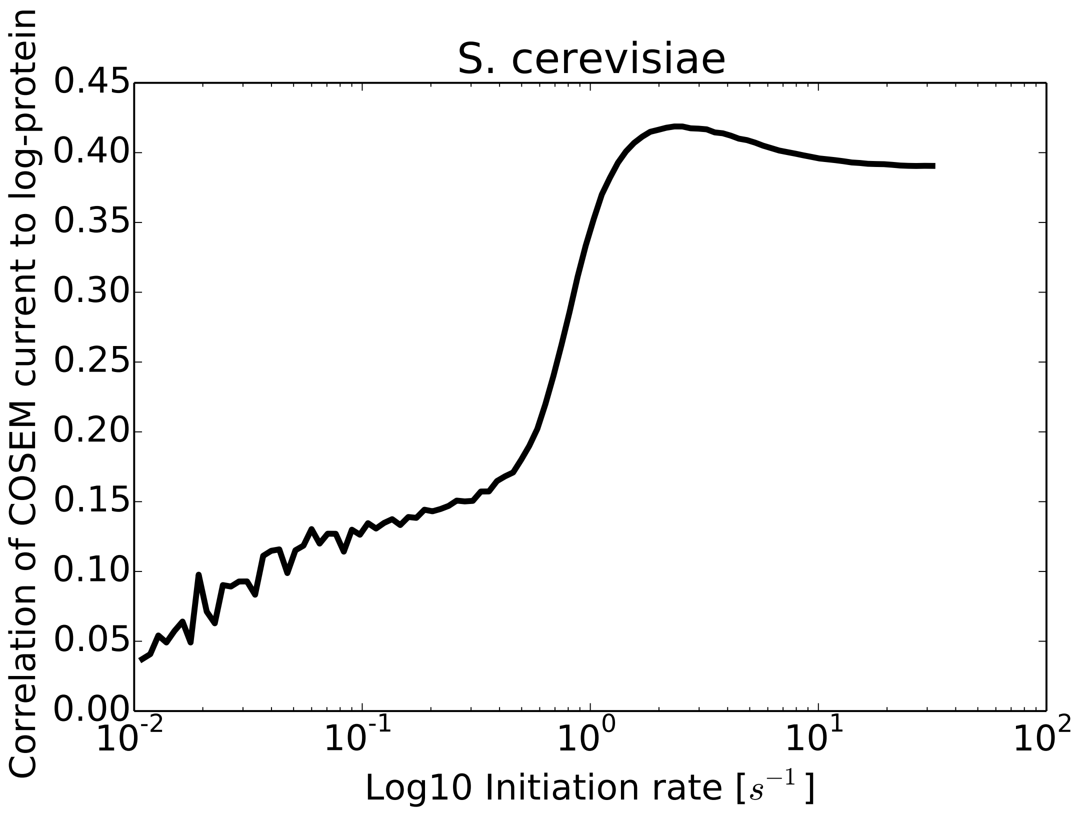

Figure S7. Correlation of COSEM current with log-protein abundance in *S. cerevisiae* genes for different initiation rates. Since there are little reliable estimates for initiation rates, an alternative is to choose the initiation rate that maximizes the correlation of COSEM current with log-protein abundance to enhance predictability. The simulations were run with the estimated drop-off rate $9.9 \times 10^{-3}\,\mathrm{s}^{-1}$ and hence, if the initiation rate is lower than the drop-off rate little correlation is expected.

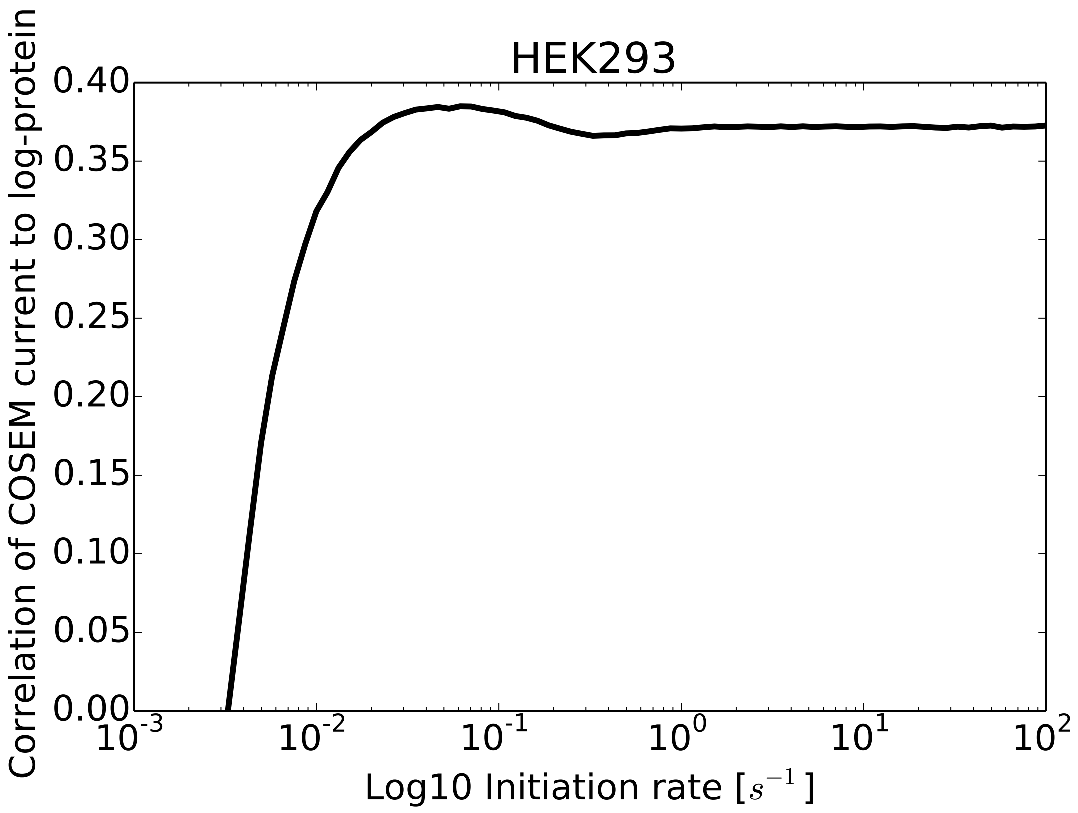

Figure S8. Correlation of COSEM current with log-protein abundance in HEK293 genes for different initiation rates. Since there are little reliable estimates for initiation rates, an alternative is to choose the initiation rate that maximizes the correlation of COSEM current with log-protein abundance to enhance predictability. The simulations were run with the estimated drop-off rate $1.8 \times 10^{-3}\,\mathrm{s}^{-1}$ and hence, if the initiation rate is lower then the drop-off rate little correlation is expected.

2.3 Sequence features and function estimates in the protein expression score

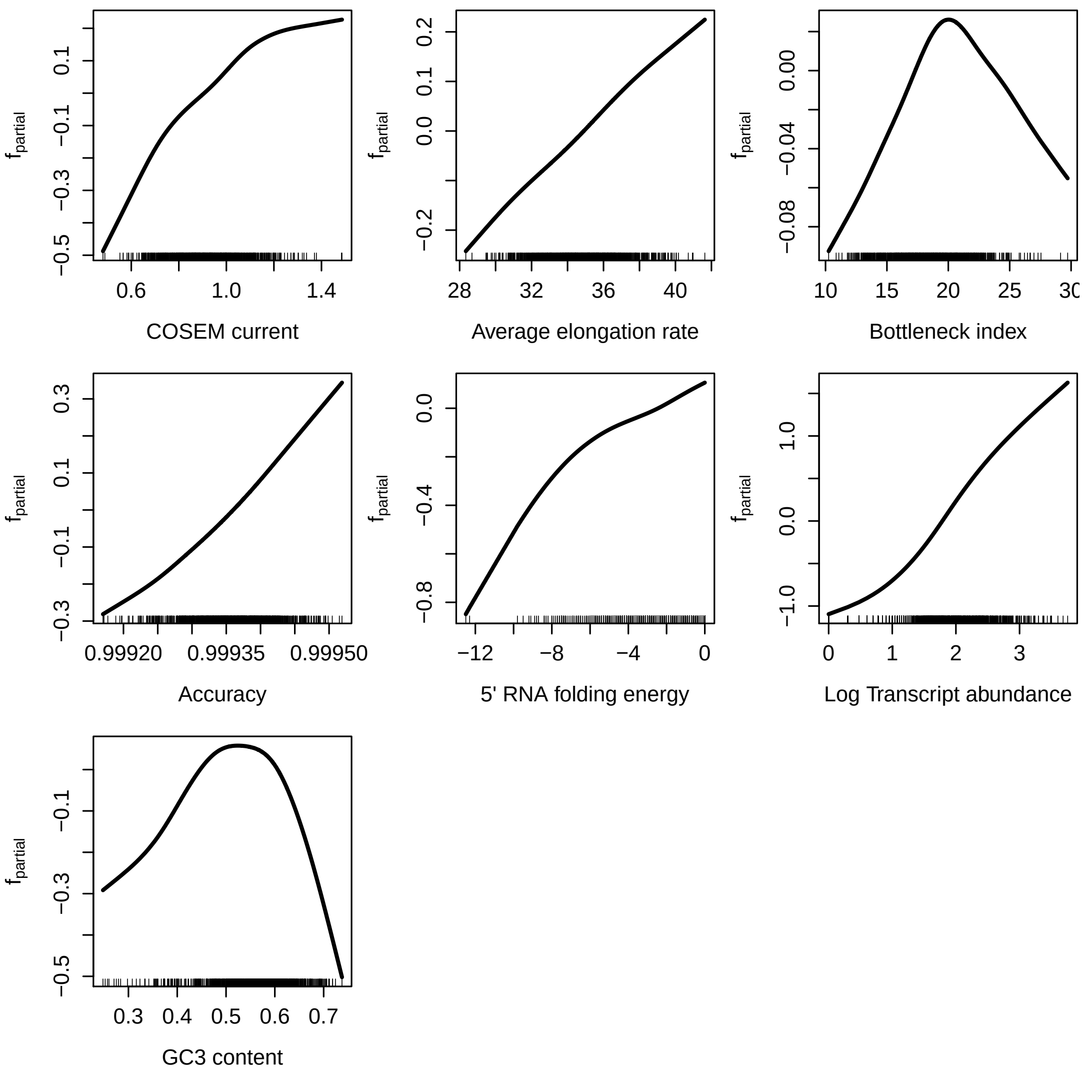

Figure S9. Function estimates representing features contributing to the protein expression score of *E. coli.* To estimate protein abundance a generalized additive model with model-based boosting was trained on 70 % of the data set. Monotonicity constraints were used for the COSEM current, average elongation rate and log-transcript abundance. Shown are only sequence features that were selected by the boosting algorithm to improve the prediction of the protein expression score. Generally, the fitted function estimates follow intuition: An increase in protein per time or COSEM current, average elongation rate, bottleneck index (less bottlenecks), accuracy, transcript abundance and folding energy (weaker folding) all contribute to higher expression whereas a balanced GC3 content appears favourable.

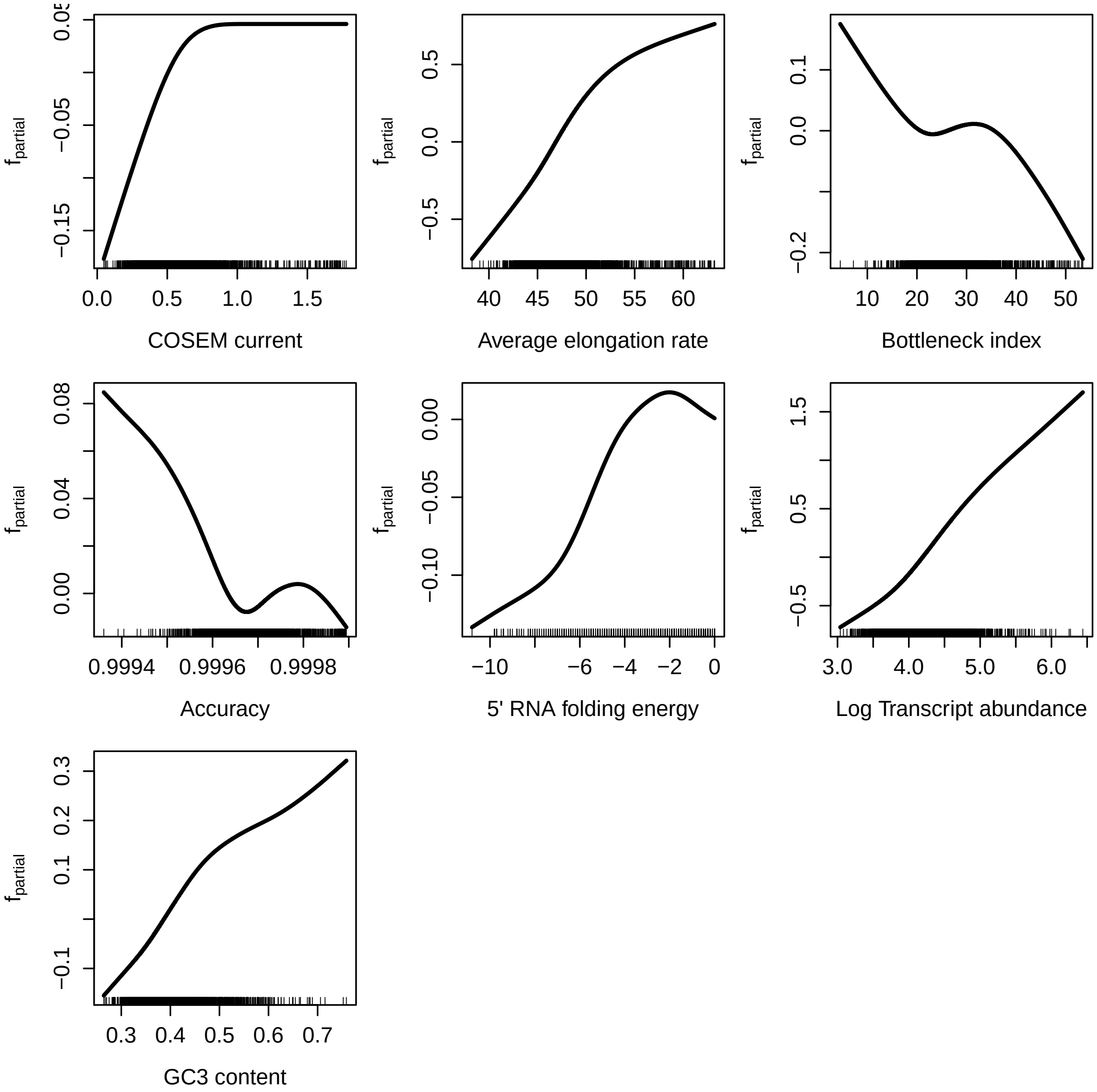

Figure S10. Function estimates representing features contributing to the protein expression score of *S. cerevisiae* as discussed in S9

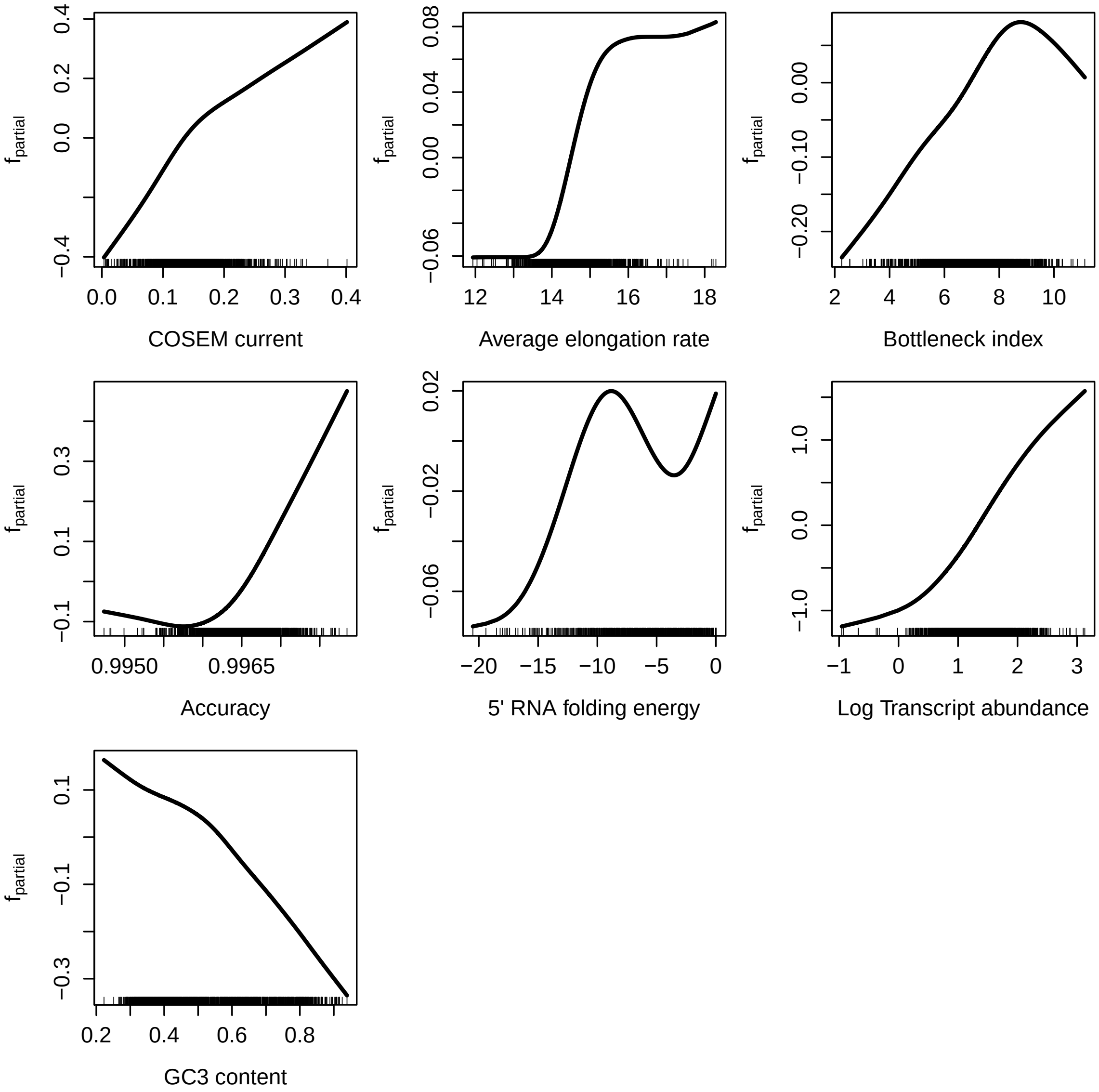

Figure S11. Function estimates representing features contributing to the protein expression score of HEK293 as discussed in S9

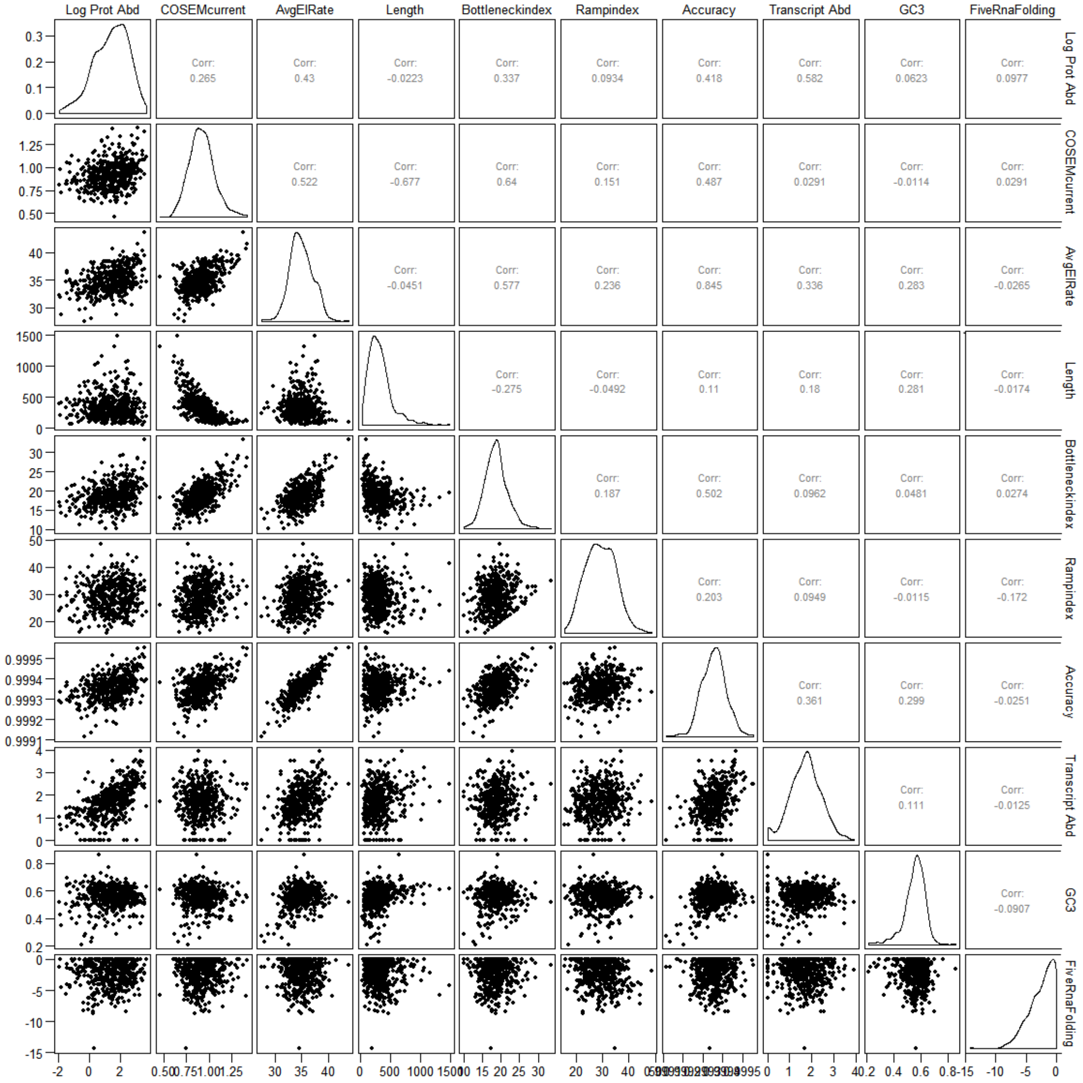

Figure S12. Correlations in sequence features considered in the protein expression score of *E. coli*. Measured logarithmic protein abundance and sequence features of *E. coli* genes were plotted pairwise with their Pearson correlation coefficients shown in the upper part of the plot matrix. Note that several features have not been selected by the boosting algorithm to contribute to the protein expression score (ramp index, sequence length, number of hairpins).

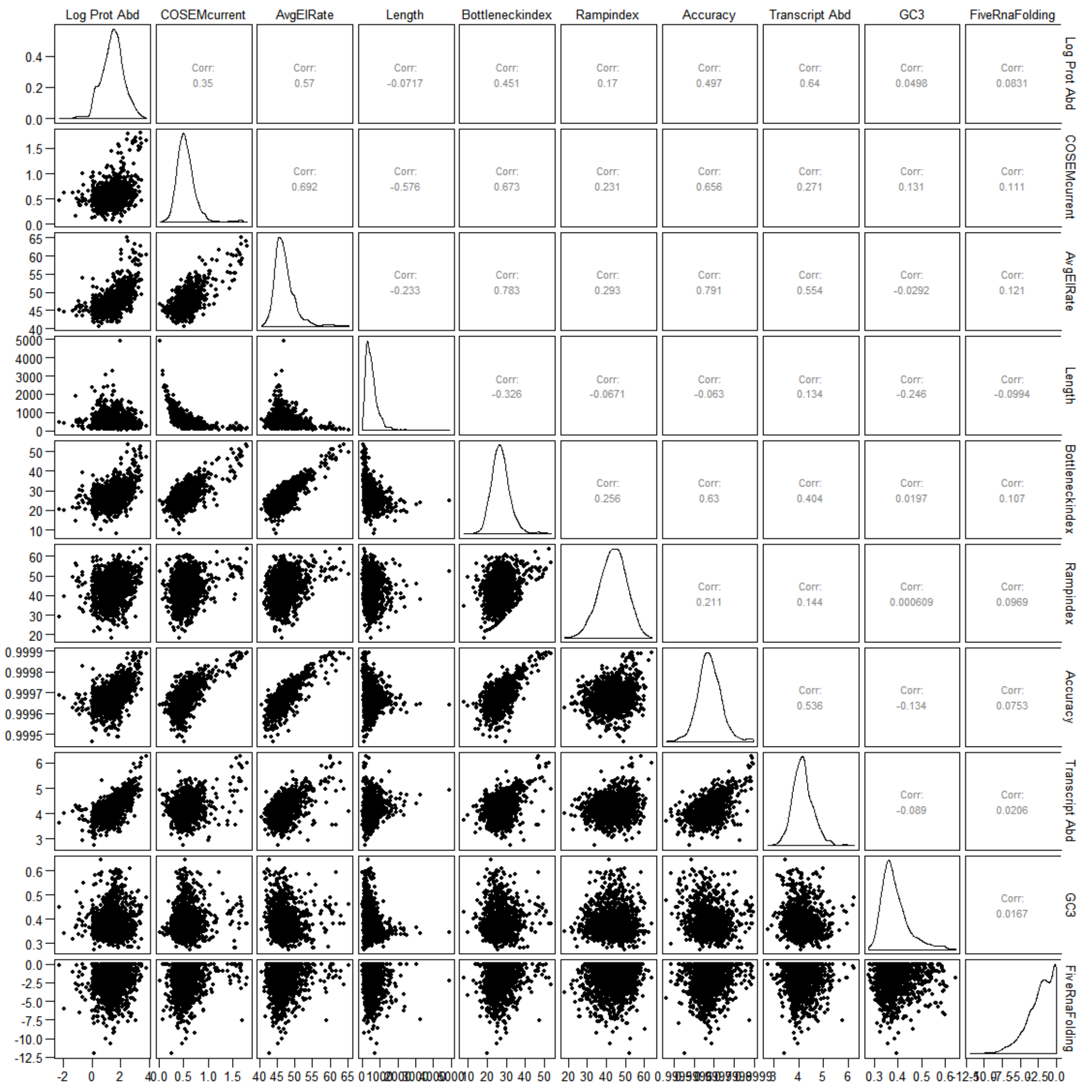

Figure S13. Correlations in sequence features considered in the protein expression score of *S. cerevisiae.* Measured logarithmic protein abundance and sequence features of *S. cerevisiae* genes were plotted pairwise with their Pearson correlation coefficients shown in the upper part of the plot matrix. Note that several features have not been selected by the boosting algorithm to contribute to the protein expression score (ramp index, sequence length, number of hairpins).

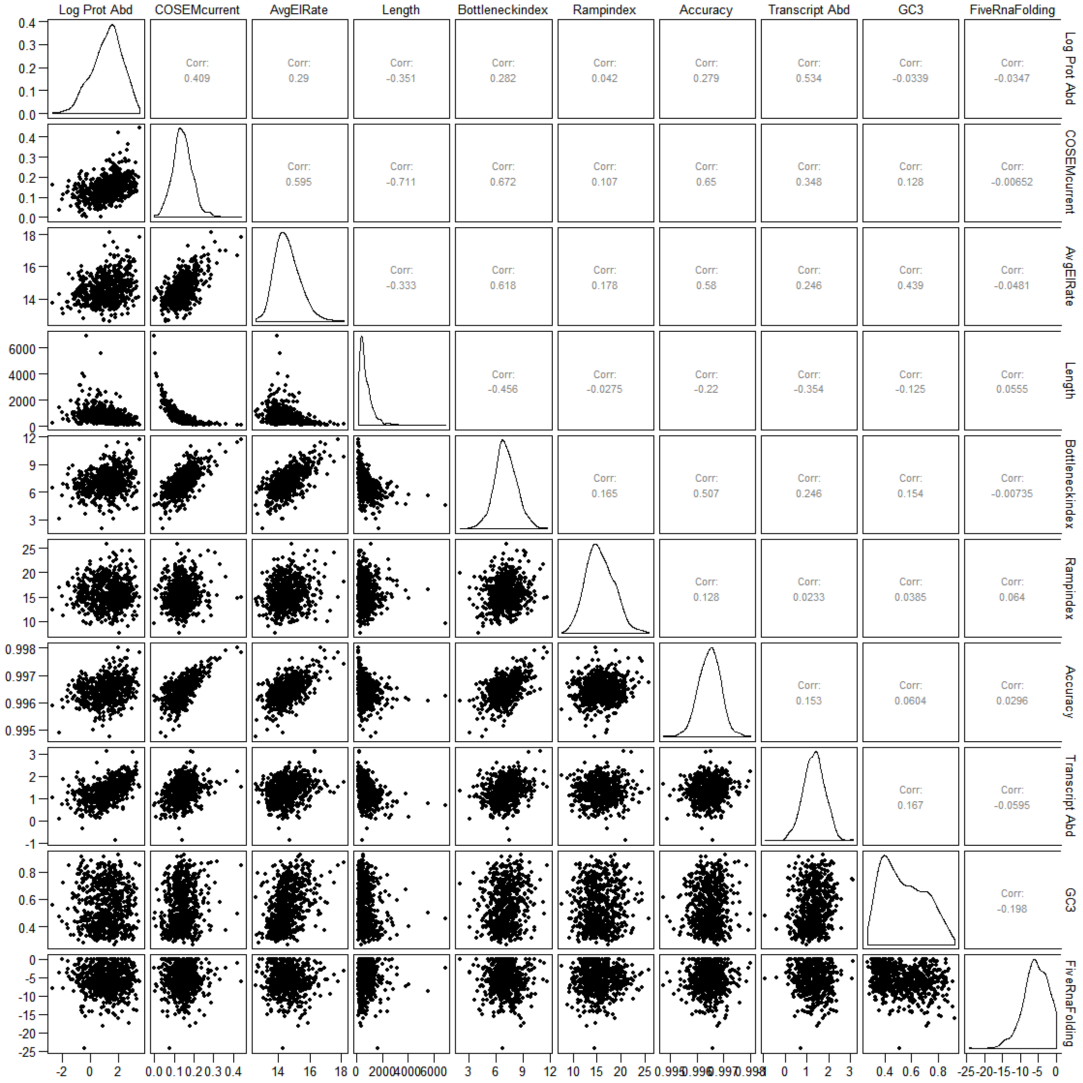

Figure S14. Correlations in sequence features considered in the protein expression score of human HEK29393 cell lines. Measured logarithmic protein abundance and sequence features of *S. cerevisiae* genes were plotted pairwise with their Pearson correlation coefficients shown in the upper part of the plot matrix. Note that several features have not been selected by the boosting algorithm to contribute to the protein expression score (ramp index, sequence length, number of hairpins).

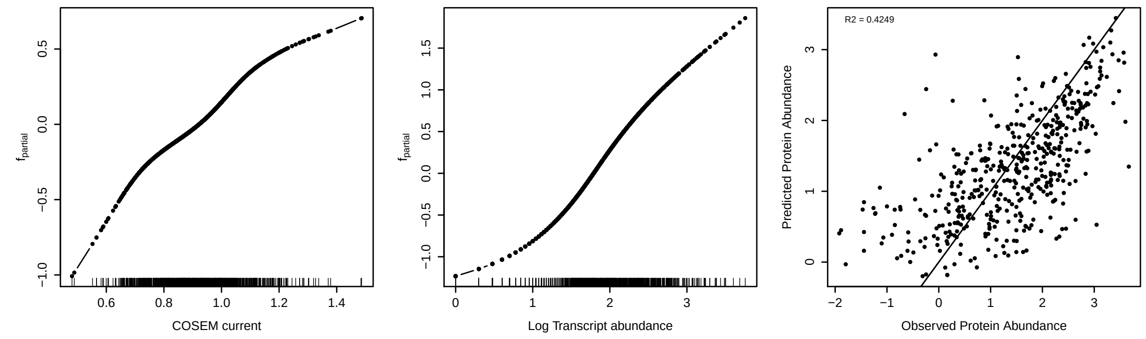

Figure S15. (Related to Figure 4) Function estimates and protein expression score against measured protein abundance for a reduced model of *E. coli.* Different from the full protein expression score, here only COSEM current and log-protein abundance were used for prediction resulting in a slightly lower but comparable coefficient of determination showing that these two features already allow for good predictions of protein abundance.

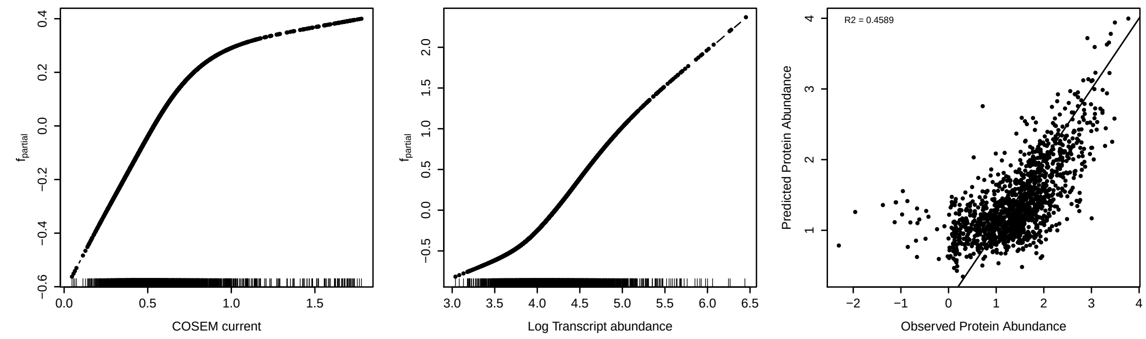

Figure S16. (Related to Figure 4) Function estimates and protein expression score against measured protein abundance for a reduced model of *S. cerevisiae* following the lines of S15

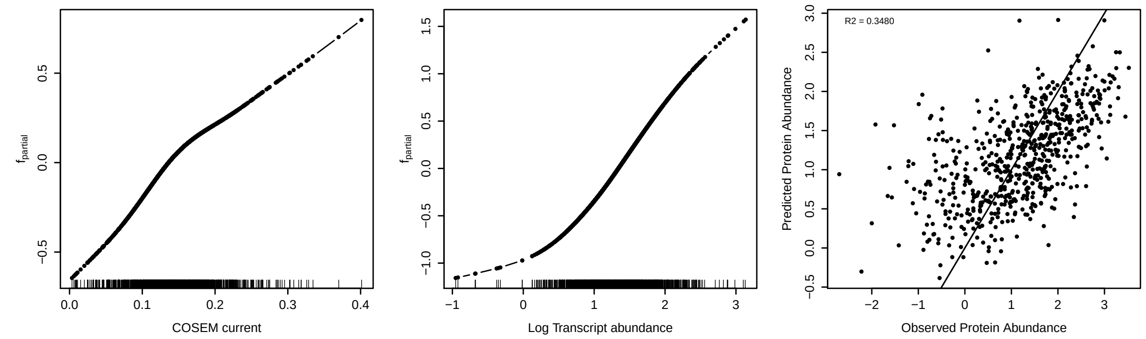

Figure S17. (Related to Figure 4) Function estimates and protein expression score against measured protein abundance for a reduced model of HEK293 following the lines of S15

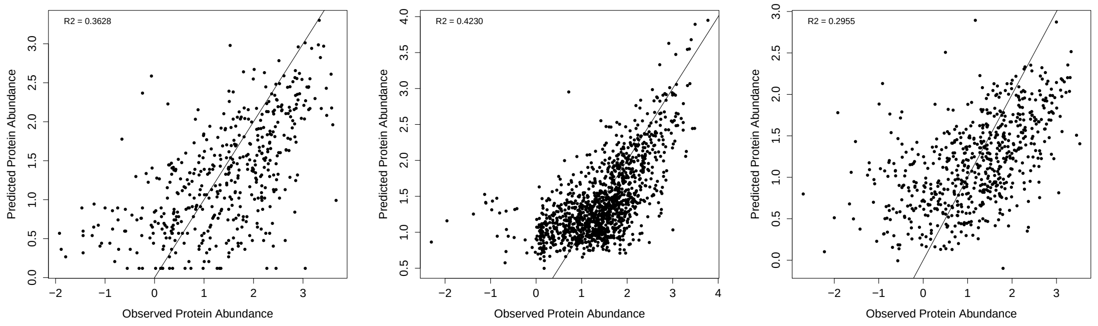

Figure S18. (Related to Figure 4) Protein expression score against measured protein abundance for reduced models of *E. coli*, *S. cerevisiae* and HEK293 taking only transcript levels into account. Different from the full protein expression score and the reduced model presented in Figures S15 to S17, here only transcript levels were used for prediction, again resulting in a lower coefficient of determination.

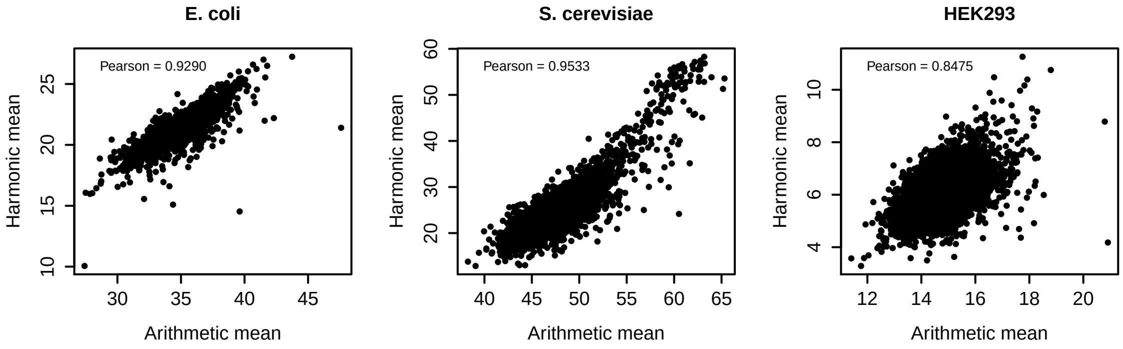

Figure S19. Harmonic and arithmetic mean of the elongation rates of all *E. coli*, *S. cerevisiae* and HEK293 sequences. Given a sequence of codons with average individual elongation times t_i the overall elongation time in the absence of ribosome queueing is, on average, $\sum_i t_i$. In terms of rates ω_i, the average overall elongation time is $\sum_i 1/\omega_i$, i.e. proportional to the inverse of the harmonic mean of elongation rates $1/\left(\sum_i 1/\omega_i\right)$. However, both, arithmetic and harmonic mean are highly correlated and can be used as predictors for protein abundance.

3 Computational and experimental studies with *ova* and *manA* variants

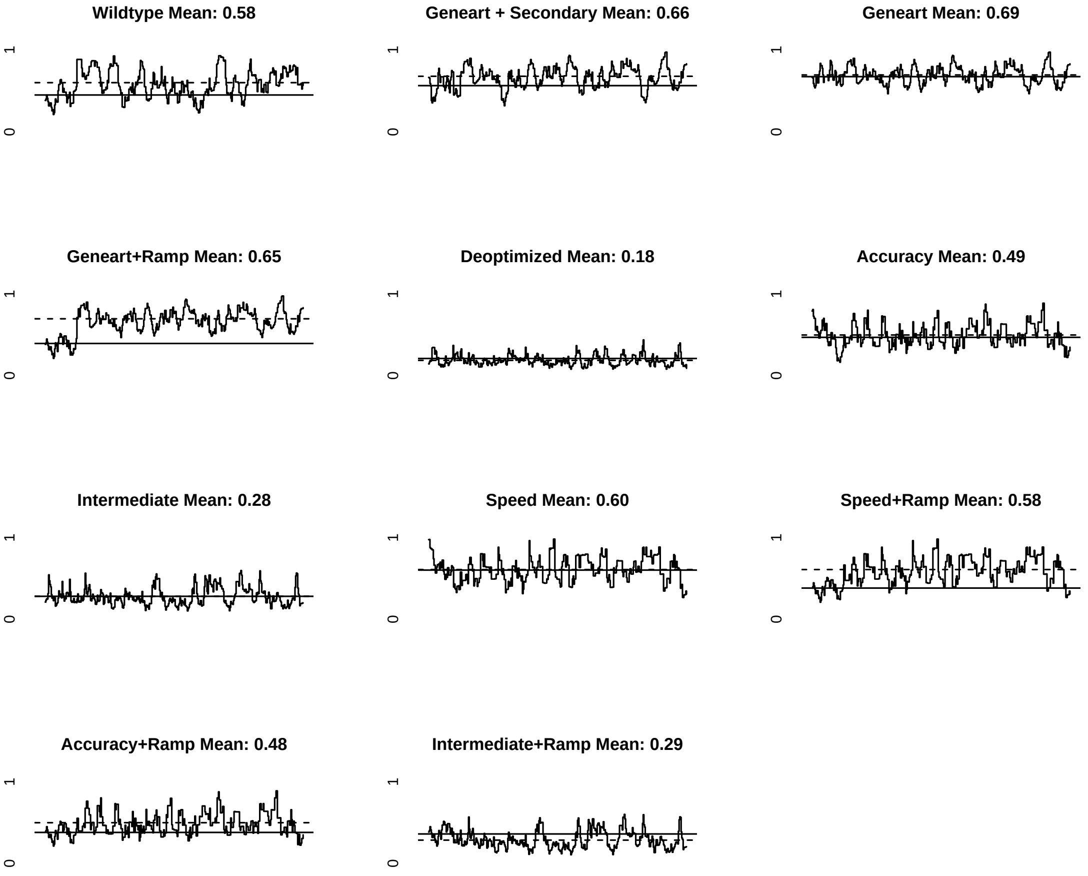

Figure S20. Rolling mean of codon adaptation index for all *manA* variants of width 10. Geometric mean of the first 50 codons' CAI is superimposed as solid line and the geometric mean of the rest of the sequences' CAI as dashed line, the sequences mean CAI is given in each panels title. The CAI is calculated with the codon usage of *Salmonella typhimurium LT2* (2). The wildtype shows an existing ramp with a ratio of 0.83 of average CAI in the first 50 codons compared to the remaining codons. The Accuracy, Intermediate and Speed variants do not distinguish between ramp and non-ramp codons. Thus the ratio is close to 1 $(0.91, 1.04, 0.98)$ in contrast to the variants with ramp and ratios of $0.77, 1.33, 0.63$ in which the Intermediate variant has an average CAI that is lower than that of the wildtype and thus lower than the wildtype-ramp leading to a ratio larger than 1. Analysis of the *manA* mRNA sequence profile with respect to CAI and average elongation rate (cf. Figure S21) led us to the conservative approach of keeping a ramp of 50 codons in *manA* variants instead of 30 codons considered otherwise.

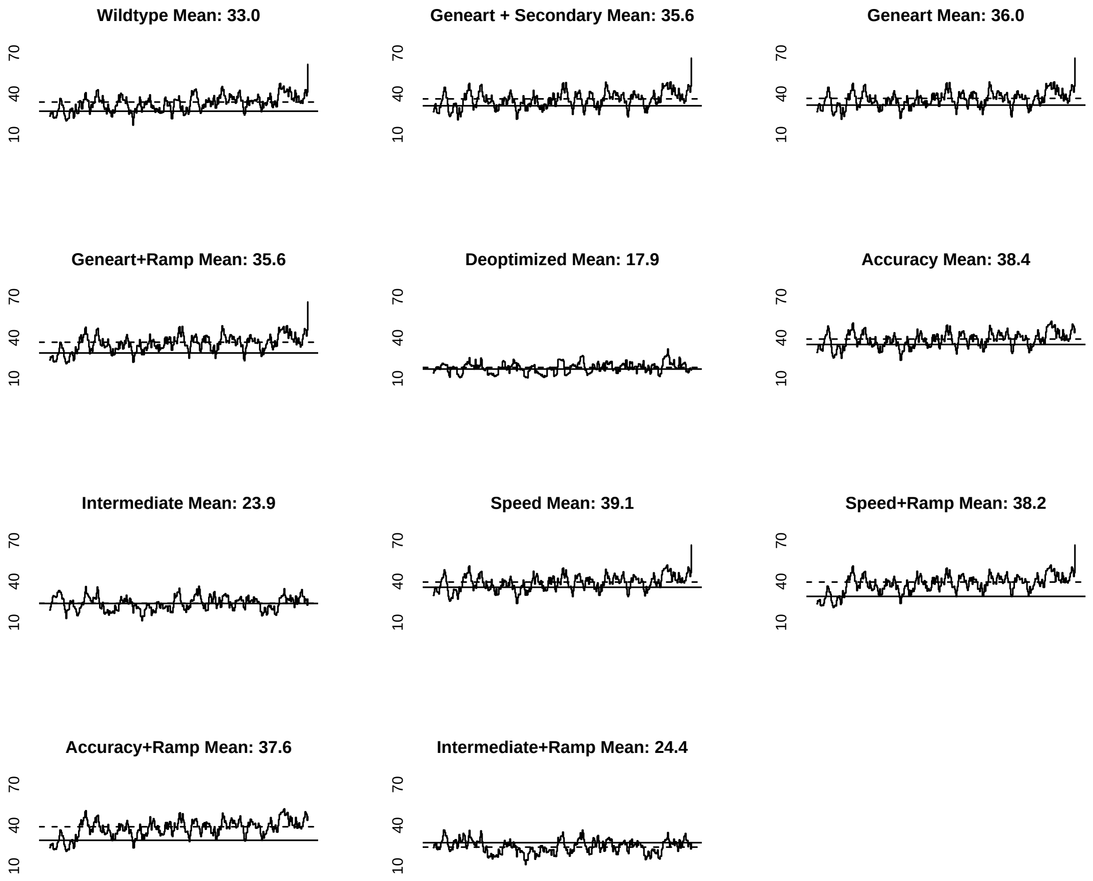

Figure S21. Rolling mean of average elongation rate for all *manA* variants of width 10. Mean elongation rate of the first 50 codons is superimposed as solid line, mean elongation rate in the rest of the sequence as dashed line, the sequences' mean elongation rate is given in each panels title (in codons per second). The figure is similar to S20 with one notable difference: Whereas the ratio of CAI of the first 50 codons to the remainder for the Geneart variant is 0.98 and hence showing no relevant ramp-effect, there is a more pronounced difference of 0.86 for the average elongation rate. Analysis of the *manA* mRNA sequence profile with respect to CAI (cf. Figure S20) and average elongation rate led us to the conservative approach of keeping a ramp of 50 codons in *manA* variants instead of 30 codons considered otherwise.

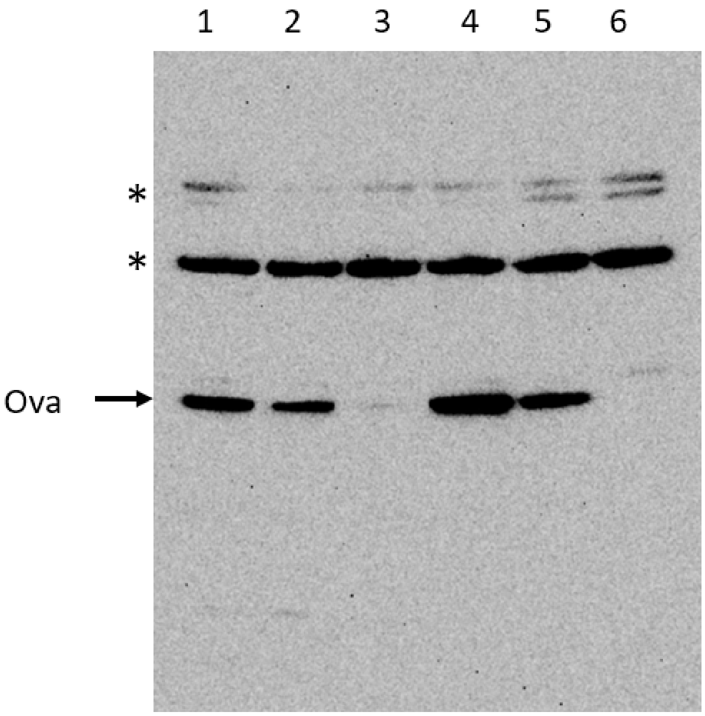

Figure S22. Ova specific Western blot. This blot corresponds to data shown in Figures 5, S24-S26. We prepared extracts of *S.* Typhimurium strains expressing variants of *ova* genes (cf. Figure 5, Table S19), i.e. wildtype (1), optimized with Geneart (2), deoptimized (3), optimized (4), intermediate (5), empty vector (6). We subjected 8 µll of each extract to analysis as described in the methods section. The figure shows uncropped data derived from one gel, the asterisks indicate unspecific bands.

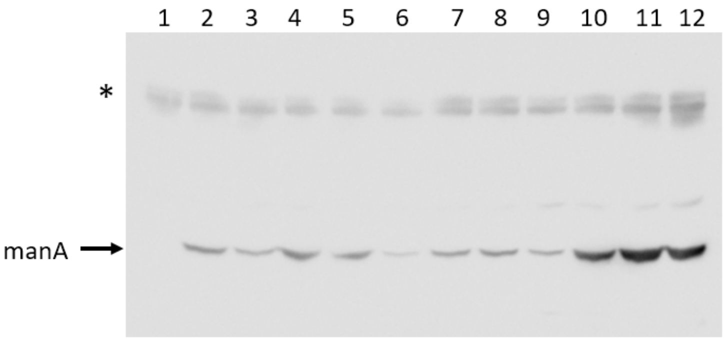

Figure S23. ManA specific Western blot. This blot corresponds to data shown in Figures 6, S24-S26. We prepared extracts of *S.* Typhimurium strains expressing *manA* genes (cf. Figure 6, Table S19), i.e. empty vector control (1), wildtype (2), optimized with Geneart with slow codons between secondary structures (3), optimized with Geneart (4), optimized with Geneart conserving ramp (5), deoptimized (6), optimized for accuracy (7), optimized for speed (8), intermediate (9), optimized for accuracy conserving ramp (10), optimized for speed with ramp (11), intermediate conserving ramp (12). We subjected 8 µl of each extract to analysis as described in the methods section. The figure shows uncropped data derived from one gel, the asterisks indicates unspecific bands.

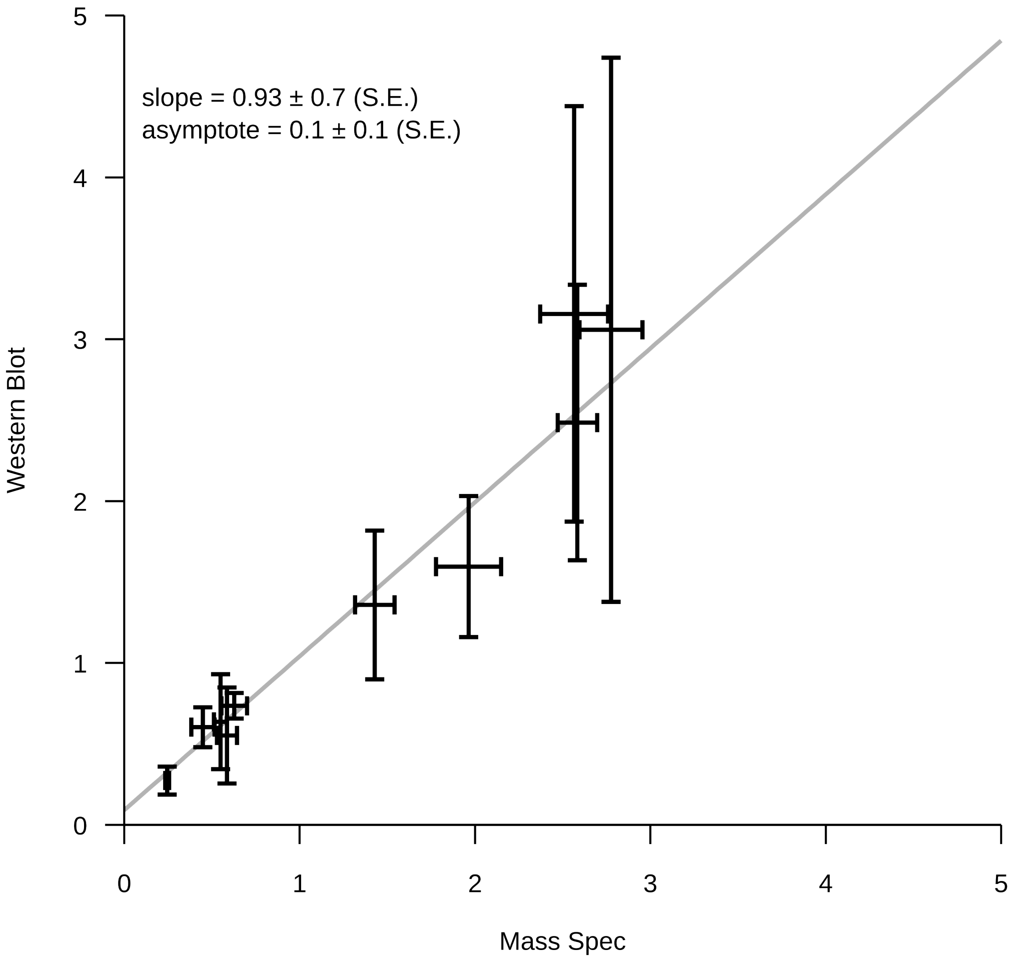

Figure S24. Comparison of mass spectrometry and Western blot protein abundance measurements. The figure shows measured protein abundance relative to wildtype with standard deviations for both methods. The linear fit takes into account the respective uncertainties according to Orear's effective variance method. The two measurement methods show very similar results, i.e. slope and asymptote of the linear fit are 0.93 ± 0.7 and 0.1 ± 0.1 and the Pearson's correlation coefficient is 0.97 with a lower and upper confidence interval of 0.8 to 0.99.

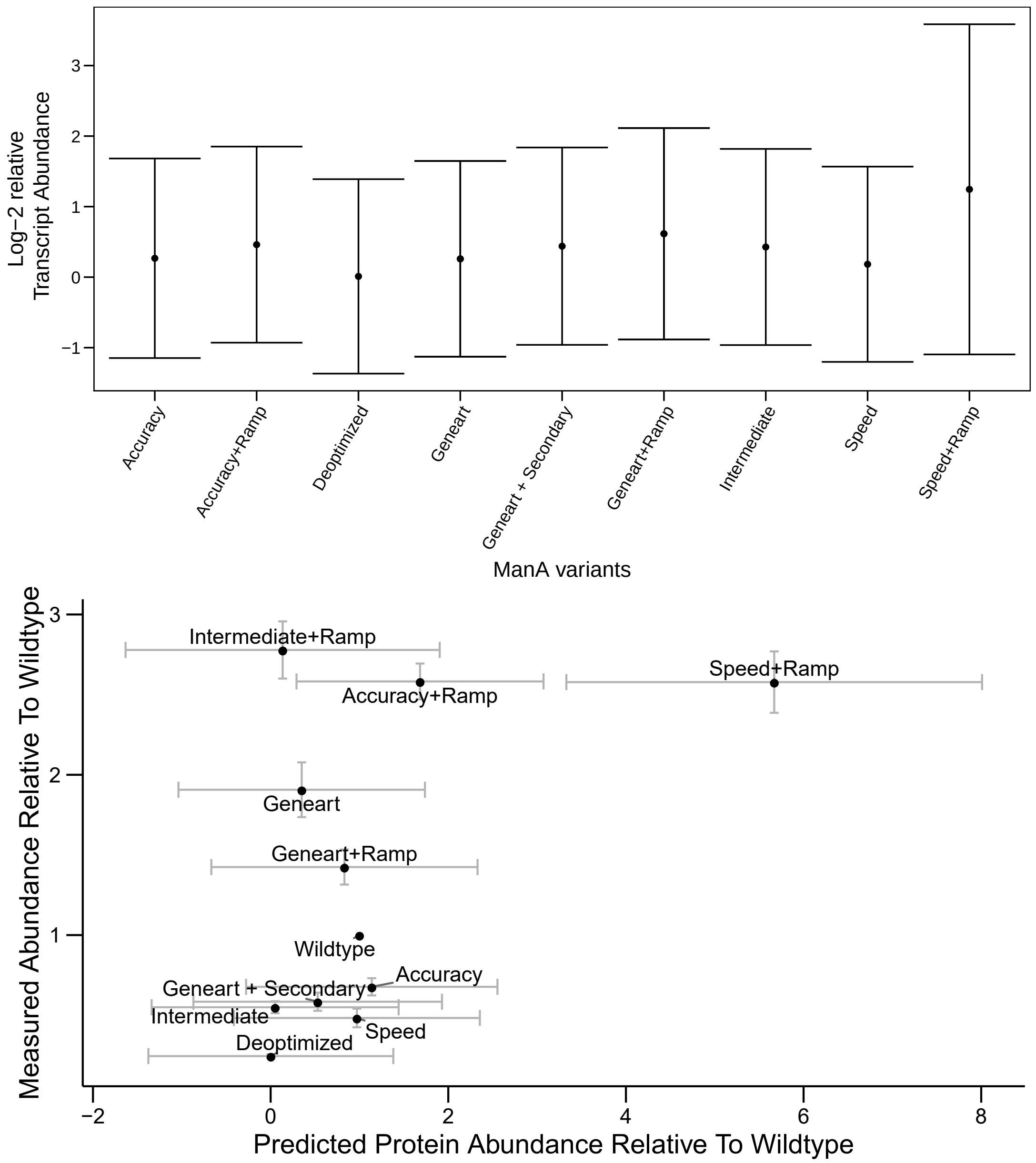

Figure S25. (Related to Figure 7) Measured and predicted protein abundance taking transcript levels into account. Top Panel: mRNA transcript levels relative to wild type were determined for *manA* variants by quantitative real-time PCR (3 biological replicates and 3 technical replicates each) and were not significantly different from each other within the errors seen in the qPCR measurements. Bottom Panel: Measured protein abundance relative to wildtype compared to protein expression score relative to wild type for *manA* variants. Protein levels are weighted averages of mass spectrometry measurements (3 biological replicates and 3 digestion replicates each) and Western blots (3–5 replicates each) which correlate well (cf. Supplementary Figure S18). Different from Fig. 6 of the main manuscript mRNA levels were considered in the protein expression score. As transcript levels are not significantly different from each other within the large errors seen in the qPCR measurements their consideration in the protein expression score results in shifts and large error bars in the predicted protein levels without adding information.

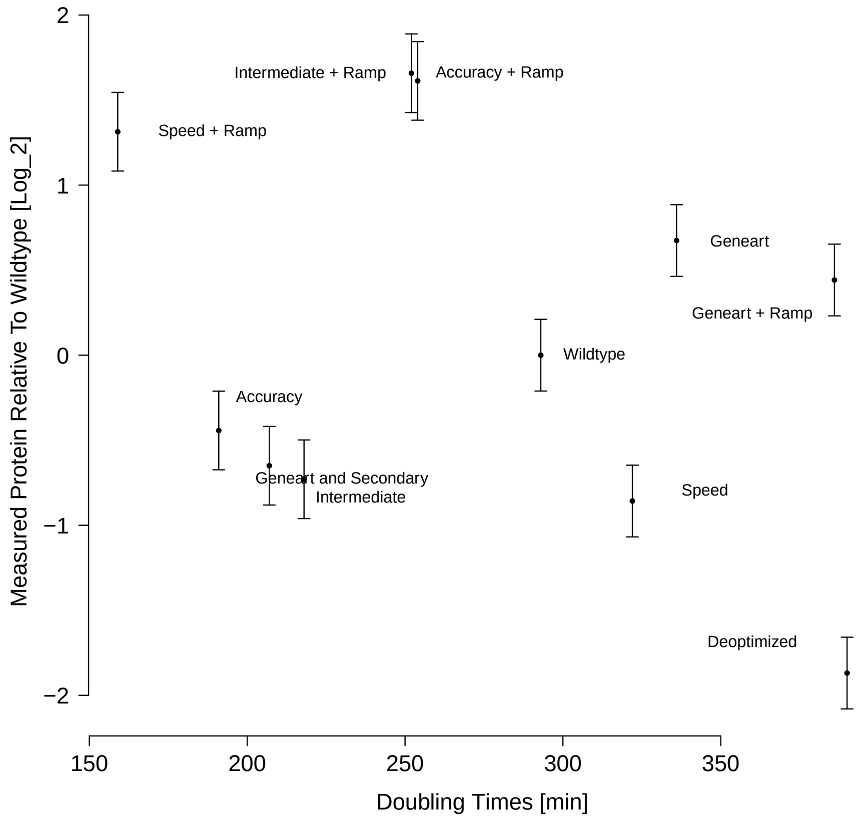

Figure S26. Protein expression levels relative to wildtype compared to doubling times of *S.* Typhimurium population on minimal mannose medium. An increase of ManA expression reduces the doubling time and hence increases the growth rate.

4 Experimental protocols

4.1 Quantification of ManA expression in *S.* Typhimurium lysates by multiple reaction monitoring (MRM)

ManA levels were determined by multiple reaction monitoring (MRM). A ManA specific peptide (YDIPELVANVK) was selected using UniProt P25081 as a template and ordered as stable isotope labelled calibration peptide (SpikeTide TQL, JPT, Berlin, Germany). This peptide carries a tag for quantification that requires trypsin cleavage before MRM analysis. A 1 nmol aliquot of the peptide was reconstituted in 100 µl of 0.1 NH_4HCO_3 containing 50 % ACN and stored at -80°C before usage. Calibration values were obtained by spiking the calibration peptide into 13.53 µl of the lysate of the *manA* deficient *S.* Typhimurium strain. DTT (9.45 µl, c=1.83 mg ml^{-1}) was added for reduction of disulfide bonds (15 min at 60 °C) and iodoacetamide (11.27 µl, c=5.1 mg ml^{-1}) for cysteine alkylation (15 min at 25°C). Enzymatic digestion (16 h at 37 °C) was done by adding trypsin (5.05 µl, c=816 ng $µl^{-1}$) and stopped by adding formic acid (5.86 µl, 5 % in water). Final concentrations of the calibration peptide can be found in Table S15.

Table S15. Final concentrations of the calibration peptide.

Concentration calibration peptide [fmol /µl]	Peptide volume [µl]	50 mmolNH_4HCO_3 volume [µl]
25	0.151	14.59
100	0.6	14.14
200	1.2	13.45
400	2.4	12.34
1000	6	8.74
2000	12	2.74

Calibration samples were digested in triplicates, each replicate was injected once at a volume of 20 µl. A volume of 13.53 µl of the lysates from strains harboring *manA* variants were combined with 2.4 µl calibration peptide, 12.34 µl NH_4HCO_3 (50 mmol), 9.54 µl DTT, 11.27 µl iodoacetamide, 5.05 µl trypsin, and 5.86 µl formic acid. Time and temperature values for reduction, alkylation and digestion were set as described above. All samples and calibration standards were frozen at −80 °C once before usage. Each sample was digested in triplicates and each triplicate was injected once at a volume of 20 µl.

For MRM analysis 20 µl of each sample were injected in an ACQUITY-UPLC equipped with an ACQUITY UPLC Peptide BEH C18 column (130 Å,1.7 µm, 2.1 mm × 100 mm) and an ACQUITY UPLC BEH C18 VanGuard Pre-column (130 Å,1.7 µm, 2.1 mm × 5 mm) (Waters, Milford, US). A binary solvent system consisting of an aqueous mobile phase (solvent A: water with 0.1 % (v/v) formic acid) and an organic mobile phase (solvent B: acetonitrile with 0.1 % formic acid) was used for the chromatographic separation. All solvents for LC-MS analysis was purchased at Biosolve (Valkenswaard, NL) unless otherwise noted. The elution gradient was 0.5 min, 3 % solvent B, 3 % to 40 % solvent B in 9.5 min, 1 min 40 % solvent B, 40 % to 90 % solvent B in 0.5 min with a constant flow rate of 300 µl min^{-1} and 1.2 min 90 % solvent B, 90 % to 3 % solvent B in 2.3 min with a flow rate of 500 µl min^{-1}.

The triple quadrupole mass spectrometer (Xevo TQ-S, Waters) was operated using MRM in positive ionization mode and scanning for 4 specific transitions of the doubly charged natural peptide YIDIPELVANVK (MH^{2+} m/z = 687.82) and the isotopically labelled standard YIDIPELVANVK* (MH^{2+} m/z = 691.39) with the following optimal acquisition parameters: capillary voltage (2.5 kV), cone voltage (21 V), desolvation temperature (200 °C), desolvation gas (800 l h^{-1}), cone gas (150 l h^{-1}). The dwell time was set to auto and the optimized collision energy for each transition is shown in Table S16.

Table S16. MRM Transitions and collision energy.

Transition	MS1	MS2	F	CE
qualifier	687.82	643.42	y6	29
quantifier	687.82	869.50	y8	20
qualifier	687.82	982.60	y9	20
qualifier	687.82	1097.60	y10	20
qualifier*	691.39	651.43	y6	29
quantifier*	691.39	877.52	y8	20
qualifier*	691.39	990.61	y9	20
qualifier*	691.39	1105.63	y10	20

F:Fragment, CE:Collision Energy, * isotopically labelled.

Data analysis was done using the TargetLynx software (V4.1 SCN810, Waters). The raw data was smoothed before integration with the mean method with width of 2 and 2 iterations. The automatic noise measurement was activated and the peak detection was done with the following values for baseline detection and peak separation: 10 (Balance), 10 (Splitting), 10 (Reduce Tailing), 0.2 (Reduce Height). The quantification of relative protein abundance was done using the peak area of the transition 687.82 $\rightarrow$ 869.50 and 691.39 $\rightarrow$ 877.52, respectively.

For the different replicates analyzed on different days we used the ratio of unlabeled to labeled peptide as a correction factor and calculated the weighted average of all replicates.

4.2 qPCR for quantification of *manA* transcripts

We performed qPCR for quantification of *manA* transcripts. First, mRNA was reverse transcribed to cDNA. Appropriate primers were designed for each *manA* variant and *cam* (cf. table S18). The product sizes were in a range of 88 to 136 bp. Bacteria were cultured in supplemented MM up to an $OD_{600} \approx 1$ and 2×10^8 bacteria were harvested at $5 \times 10^3 \times$g for 5 min. RNAprotect Bacteria Reagent and RNeasy Plus Mini Kit were used for purification of RNA (Qiagen). 380 ng RNA of each amsple was used for cDNA synthesis employing SuperScript III Reverse Transcriptase (ThermoFisher). qPCR reactions were set up using the iTaq Universal SYBR Green Supermix (Bio-Rad) and were run on a CFX96 thermal cycler (Bio-Rad). Raw-data were analyzed using the CFX Manager Software (Bio-Rad) and C_q values calculated by thresholding.

For relative quantification of *manA* transcripts primer efficiencies were measured by using a dilution series of *manA* and *cam* transcripts and additionally via sigmoidal curve fitting in R.

5 Data retrieval and studied gene sequences

Table S17. Data sources for protein expression and mRNA transcript levels. Data sets for protein abundance from PaxDb (5) are provided in the database's common abundance metric in parts per million (ppm) for the human HEK293 cell line, *E. coli* and *S. cerevisiae*. Transcript abundance was estimated from RNA-seq experiments in which FPKM (number of Fragments Per Kilobase of transcript per Million mapped reads in an RNA-Seq experiment) was taken as a proxy for transcript abundance. For model based boosting log-transformed data were used. Latest date of retrieval was 2016/07/21.

Data	Source
HEK 293 Protein	`https://pax-db.org/dataset/9606/329/`
HEK 293 Transcript	`https://www.ncbi.nlm.nih.gov/geo/query/acc.cgi?acc=GSE38356`
E. coli Protein	`http://pax-db.org/downloads/latest/datasets/511145/511145-WHOLE_ORGANISM-integrated.txt`
E. coli Transcript	`https://www.ncbi.nlm.nih.gov/geo/query/acc.cgi?acc=GSM533304`
S. cerevisiae Protein	`http://pax-db.org/downloads/4.0/datasets/4932/4932-WHOLE_ORGANISM-integrated.txt`
S. cerevisiae Transcript	`https://www.ncbi.nlm.nih.gov/geo/query/acc.cgi?acc=GSM1557444`

Table S18. Ologinucleotides used.

Name	Target	5'–3' sequence
oHL20	kan of pKD4	GCCTGCTTGCCGAATATC
oJT1	pKD4	ATGCAAAAACTCATTAACTCAGTGCA AAACTATGCCTGGGTGTGTAGGCTGG AGCTGCTTC
oJT2	pKD4	CTACAGCTTGTTATAAACACGCGCTA AACGGCCCGTGCCGCTGGCGCATATG AATATCCTCCTTAG
oJT4	downstream region of genomic manA	GAAACCAGGCGGATTAAACC
oJT7	wt manA cloning	GGATATCATATGCAAAAACTCATTAA CTCAG
oJT8	wt manA cloning	GGATATCACTAGTCTACAGCTTGTTA TAAACACG
oJT11	cam	TCCGGCCTTTATTCACATTC
oJT12	cam	CGTTTCAGTTTGCTCATGGA
oJT13	manA promoter	CCTCCCATTGATCTCCACAT
oJT14	manA2	GGTCAGTGCGGTTTTGCTAC
oJT16	manA3, 8, 9, 10	GATTGGCGATGCCATAAAGT
oJT17	wt manA	ATTGGCGATGCCATAAAGTT
oJT21	manA	GTTTTAGAGCCCCATGCGTA
oJT23	manA5	GTTTGCGATGCCGTACAGTT
oJT24	manA6	GGGTTGGCTATACCGTACAA
oJT27	manA1	CCATACAGTTCGGTCAGTGC
oJT28	manA4	TGGTTGTTGTTGTGGATTGG

Table S19. List of all sequences used. The table lists internal ids for documentation purposes (plasmid, name, gid, DNA, GK), alternate long names and coding sequences.

Plasmid	gid	name	DNA	GK	Long name	Alternate name	CDS
pETcoco-I	0	w/o	375	393			
pJT6a	wt	manAwt	685	397	manA_orig	Wildtype	ATGCAAAAACTCATTAACTCAGTGCAAAACTATG<br>CCTGGGGAAGTAAAACTGCGTTAACGGAACTTT<br>ATGGCATCGCCAATCCGCAGCAGCAGCCAATGG<br>CTGAACTCTGGATGGGCGCGCATCCCAAAAGCA<br>GCTCGCGAATCACCACCGCCAACGGCGAAACCG<br>TCTCCCTGCGTGACGCCATCGAAAAGAATAAAA<br>CCGCCATGCTGGGCGAAGCGGTAGCCAACCGTT<br>TCGGCGAACTGCCGTTTCTGTTTAAAGTACTGT<br>GCGCCGCACAACCGCTCTCTATTCAGGTGCACC<br>CGAATAAACGCAACTCCGAAATCGGTTTCGCGA<br>AAGAAAATGCGGCGGGTATCCCCATGGATGCCG<br>CAGAGCGGAACTATAAAGATCCTAACCATAAAC<br>CAGAGCTGGTTTTTGCCCTGACGCCTTTCCTGG<br>CGATGAACGCGTTCCGCGAATTTTCTGACATTG<br>TCTCTTTACTGCAACCTGTCGCCGGCGCGCATTC<br>CGCTATCGCCCACTTTTTGCAGGTGCCGAATGCT<br>GAACGTCTGAGCCAGCTTTTCGCCAGCCTGTTG<br>AATATGCAAGGCGAAGAAAAATCCCGCGCGTTA<br>GCCGTACTCAAAGCGGCGCTTAACAGCCAGCAA<br>GGCGAACCGTGGCAAACGATCCGCGTGATTTCA<br>GAGTATTATCCTGACGACAGCGGGCTTTTCTCTC<br>CTTTGTTGCTGAATGTGGTCAAACTGAATCCCG<br>GCGAGGCGATGTTCCTGTTTGCTGAAACGCCTC<br>ATGCTTATCTGCAGGGCGTTGCGCTGGAAGTCA<br>TGGCGAACTCCGATAACGTTCTGCGCGCTGGCC<br>TTACGCCAAAATATATCGACATCCCTGAGCTGGT<br>CGCGAACGTGAAGTTCGAACCTAAGCCTGCCGG<br>CGAGTTGCTGACTGCCCCGGTGAAAAGCGGCGC<br>GGAGCTGGACTTCCCAATTCCGGTTGACGATTT<br>TGCTTTTTCACTGCACGACCTGGCGCTTCAGGA<br>GACGAGCATCGGCCAACACAGCGCCGCGATTCT<br>GTTCTGCGTTGAGGGTGAGGCGGTGTTACGTAA<br>AGATGAACAGCGTCTGGTACTGAAGCCGGGTGA<br>ATCTGCCTTTATCGGCGCGGATGAGTCTCCGGT<br>TAACGCCAGCGGCACGGGCCGTTTAGCGCGTGT<br>TTATAACAAGCTGTAG

Table S19. All sequences used cont.

Plasmid	gid	name	DNA	GK	Long name	Alternate name	CDS
pJT7a	1	manA1	680	531	manA_opt_and_slow_before_secondary	Geneart + Secondary	ATGCAGAAACTGATTAATAGCGTGCAGAATTATGCATGGGGATCGAAAACCGCACTGACCGAACTGTATGGTATTGCAAATCCGCAGCAGCAGCCAATGGCAGAACTGTGGATGGGTGCACATCCGAAAAGCAGCTCCCGAATTACCACCGCAAATGGTGAAACCGTTAGCCTGCGTGATGCAATCGAAAAAAAACAAAACCGCCATGCTGGGTGAAGCAGTTGCAAATCGTTTTGGTGAACTGCCGTTTCTGTTTAAAGTTCTGTGTGCAGCACAGCCGCTGAGCATTCAGGTTCATCCGAATAAACGTAATAGCGAAATTGGCTTTGCCAAAGAAAATGCAGCAGGTATTCCCATGGATGCAGCAGAACGTAACTATAAAGATCCGAATCATAAACCAGAACTGGTTTTTGCACTGACCCCGTTTCTGGCAATGAATGCATTTCGTGAATTTAGCGATATTGTGAGCCTGCTGCAGCCGGTTGCCGGTGCCCATAGCGCAATTGCACATTTTCTGCAGGTTCCGAATGCCGAACGTCTGAGCCAGCTGTTTGCAAGCCTGCTGAATATGCAGGGTGAAGAAAAAAGCCGTGCACTGGCAGTTCTGAAAGCAGCACTGAATAGCCAGCAGGGCGAACCGTGGCAGACCATTCGTGTTATTAGCGAATATTATCCGGATGATAGCGGTCTTTTTAGCCCTCTGCTGCTGAATGTTGTTAAACTGAATCCGGGTGAAGCCATGTTTCTGTTCGCAGAAACACCGCATGCTTATCTGCAGGGTGTTGCACTGGAAGTTATGGCAAATAGCGATAATGTTCTGCGTGCAGGTCTGACCCCGAAATACATTGATATTCCAGAACTGGTTGCCAACGTGAAATTTGAACCGAAACCGGCAGGCGAACTGCTGACCGCACCGGTTAAAAGCGGTGCAGAACTGGATTTTCCGATTCCGGTGGATGATTTTGCATTTAGTTTGCATGATCTGGCACTGCAAGAAACCAGCATTGGTCAGCATAGCGCAGCAATTCTGTTTTGTGTTGAAGGTGAAGCCGTTCTGCGTAAAGATGAACAGCGTCTGGTTCTGAAACCTGGTGAAAGCGCATTTATTGGTGCAGATGAAAGTCCGGTTAATGCAAGCGGCACCGGTCGTCTGGCACGTGTTTATAACAAACTGTAA

Table S19. All sequences used cont.

Plasmid	gid	name	DNA	GK	Long name	Alternate name	CDS
pJT8a	2	manA2	678	532	man_opt_geneart	Geneart	ATGCAGAAACTGATTAATAGCGTGCAGAATTAT GCATGGGGTAGCAAAACCGCACTGACCGAACTG TATGGTATTGCAAATCCGCAGCAGCAGCCGATG GCAGAACTGTGGATGGGTGCACATCCGAAAAGC AGCAGTCGTATTACCACCGCAAATGGTGAAACC GTTAGCCTGCGTGATGCAATCGAAAAAAACAAA ACCGCCATGCTGGGTGAAGCAGTTGCAAATCGT TTTGGTGAACTGCCGTTTCTGTTTAAAGTTCTGT GTGCAGCACAGCCGCTGAGCATTCAGGTTCATC CGAATAAACGTAATAGCGAAATTGGCTTTGCCA AAGAAAATGCAGCAGGTATTCCGATGGATGCAG CAGAACGTAACTATAAAGATCCGAATCATAAACC GGAACTGGTTTTTGCACTGACCCCGTTTCTGGC AATGAATGCATTTCGTGAATTTAGCGATATTGT GAGCCTGCTGCAGCCGGTTGCCGGTGCACATAG CGCAATTGCACATTTTCTGCAGGTTCCGAATGC AGAACGTCTGAGCCAGCTGTTTGCAAGCCTGCT GAATATGCAGGGTGAAGAAAAAAGCCGTGCACT GGCAGTTCTGAAAGCAGCACTGAATAGCCAGCA GGGCGAACCGTGGCAGACCATTCGTGTTATTAG CGAATATTATCCGGATGATAGCGGTCTGTTTAG CCCTCTGCTGCTGAATGTTGTTAAACTGAATCCG GGTGAAGCCATGTTTCTGTTCGCAGAAACACCG CATGCATATCTGCAGGGTGTTGCACTGGAAGTT ATGGCAAATAGCGATAATGTTCTGCGTGCAGGT CTGACCCCGAAATACATTGATATTCCAGAACTGG TTGCCAACGTGAAATTTGAACCGAAACCGGCAG GCGAACTGCTGACCGCACCGGTTAAAAGCGGTG CAGAACTGGATTTTCCGATTCCGGTGGATGATT TTGCATTTAGTCTGCATGATCTGGCACTGCAAG AAACCAGCATTGGTCAGCATAGCGCAGCAATTC TGTTTTGTGTTGAAGGTGAAGCCGTTCTGCGTA AAGATGAACAGCGTCTGGTTCTGAAACCTGGTG AAAGCGCATTTATTGGTGCAGATGAAAGTCCGG TTAATGCAAGCGGCACCGGTCGTCTGGCACGTG TTTATAACAAACTGTAA

Table S19. All sequences used cont.

Plasmid	gid	name	DNA	GK	Long name	Alternate name		CDS
pJT9a	3	manA3	682	533	manA_opt_and_ramp	Geneart Ramp	+	ATGCAAAAACTCATTAACTCAGTGCAAAACTATG<br>CCTGGGGAAGTAAAACTGCGTTAACGGAACTTT<br>ATGGCATCGCCAATCCTCAGCAGCAGCCAATGG<br>CTGAACTCTGGATGGGCGCGCATCCCAAAAGCT<br>CATCGCGAATCACGACCGCGAATGGTGAAACCG<br>TTAGCCTGCGTGATGCAATCGAAAAAAACAAAA<br>CCGCCATGCTGGGTGAAGCAGTTGCAAATCGTT<br>TTGGTGAACTGCCGTTTCTGTTTAAAGTTCTGT<br>GTGCAGCACAGCCGCTGAGCATTCAGGTTCATC<br>CGAATAAACGTAATAGCGAAATTGGCTTTGCCA<br>AAGAAAATGCAGCAGGTATTCCGATGGATGCAG<br>CAGAACGTAACTATAAAGATCCGAATCATAAACC<br>AGAACTGGTTTTTGCACTGACCCCGTTTCTGGC<br>AATGAATGCATTTCGTGAATTTAGCGATATTGT<br>GAGCCTGCTGCAGCCGGTTGCCGGTGCCCATAG<br>CGCAATTGCACATTTTCTGCAGGTTCCGAATGCC<br>GAACGTCTGAGCCAGCTGTTTGCAAGCCTGCTG<br>AATATGCAGGGTGAAGAAAAAAGCCGTGCACTG<br>GCAGTTCTGAAAGCAGCACTGAATAGCCAGCAG<br>GGCGAACCGTGGCAGACCATTCGTGTTATTAGC<br>GAATATTATCCGGATGATAGCGGTCTTTTTAGC<br>CCTCTGCTGCTGAATGTTGTTAAACTGAATCCG<br>GGTGAAGCCATGTTTCTGTTCGCAGAAACACCG<br>CATGCTTATCTGCAGGGTGTTGCACTGGAAGTT<br>ATGGCAAATAGCGATAATGTTCTGCGTGCAGGT<br>CTGACCCCGAAATACATTGATATTCCAGAACTGG<br>TTGCCAACGTGAAATTTGAACCGAAACCGGCAG<br>GCGAACTGCTGACCGCACCGGTTAAAAGCGGTG<br>CAGAACTGGATTTTCCGATTCCGGTGGATGATT<br>TTGCATTTAGTTTGCATGATCTGGCACTGCAAG<br>AAACCAGCATTGGTCAGCATAGCGCAGCAATTC<br>TGTTTTGTGTTGAAGGTGAAGCCGTTCTGCGTA<br>AAGATGAACAGCGTCTGGTTCTGAAACCTGGTG<br>AAAGCGCATTTATTGGTGCAGATGAAAGTCCGG<br>TTAATGCAAGCGGCACCGGTCGTCTGGCACGTG<br>TTTATAACAAACTGTAA

Table S19. All sequences used cont.

Plasmid	gid	name	DNA	GK	Long name	Alternate name	CDS
pJT27a	4	manA4a	746	528	manA_orig OptSpeedF old	Deoptimized	ATGCAAAAGCTAATAAATTCCGTCCAAAATTATG CCTGGGGATCCAAGACCGCCCTAACCGAGCTAT ATGGAATAGCCAATCCACAACAACAACCAATGG CCGAGCTATGGATGGGAGCCCATCCAAAGTCCT CCTCCCGGATAACCACCGCCAATGGAGAGACCG TCTCCCTACGGGATGCCATAGAGAAGAATAAGA CCGCCATGCTAGGAGAGGCCGTCGCCAATCGGT TTGGAGAGCTACCATTTCTATTTAAGGTCCTATG TGCCGCCCAACCACTATCCATACAAGTCCATCCA AATAAGCGGAATTCCGAGATAGGATTTGCCAAG GAGAATGCCGCCGGAATACCAATGGATGCCGCC GAGCGGAATTATAAGGATCCAAATCATAAGCCA GAGCTAGTCTTTGCCCTAACCCCATTTCTAGCCA TGAATGCCTTTCGGGAGTTTTCCGATATAGTCTC CCTACTACAACCAGTCGCCGGAGCCCATTCCGCC ATAGCCCATTTTCTACAAGTCCCAAATGCCGAGC GGCTATCCCAACTATTTGCCTCCCTACTAAATAT GCAAGGAGAGGAGAAGTCCCGGGCCCTAGCCGT CCTAAAGGCCGCCCTAAATTCCCAACAAGGAGA GCCATGGCAAACCATACGGGTCATATCCGAGTA TTATCCAGATGATTCCGGACTATTTTCCCCACTA CTACTAAATGTCGTCAAGCTAAATCCAGGAGAG GCCATGTTTCTATTTGCCGAGACCCCACATGCCT ATCTACAAGGAGTCGCCCTAGAGGTCATGGCCA ATTCCGATAATGTCCTACGGGCCGGACTAACCCC AAAGTATATAGATATACCAGAGCTAGTCGCCAA TGTCAAGTTTGAGCCAAAGCCAGCCGGAGAGCT ACTAACCGCCCCAGTCAAGTCCGGAGCCGAGCT AGATTTTCCAATACCAGTCGATGATTTTGCCTTT TCCCTACATGATCTAGCCCTACAAGAGACCTCCA TAGGACAACATTCCGCCGCCATACTATTTTGTGT CGAGGGAGAGGCCGTCCTACGGAAGGATGAGCA ACGGCTAGTCCTAAAGCCAGGAGAGTCCGCCTT TATAGGAGCCGATGAGTCCCCAGTCAATGCCTC CGGAACCGGACGGCTAGCCCGGGTCTATAATAA GCTATAG

Table S19. All sequences used cont.

Plasmid	gid	name	DNA	GK	Long name	Alternate name	CDS
pJT28	5	manA5	727	460	manA_orig Opt5050	Intermediate	ATGCAGAAACTGATCAACTCAGTTCAGAACTAC GCATGGGGCTCAAAAACGGCACTGACGGAACTG TACGGCATCGCAAACCCCCAGCAGCAGCCCATG GCAGAACTGTGGATGGGCGCACACCCCAAATCA TCATCACGAATCACGACGGCAAACGGCGAAACG GTTTCACTGCGAGACGCAATCGAAAAAAACAAA ACGGCAATGCTGGGCGAAGCAGTTGCAAACCGA TTCGGCGAACTGCCCTTCCTGTTCAAAGTTCTGT GCGCAGCACAGCCCCTGTCAATCCAGGTTCACCC CAACAAACGAAACTCAGAAATCGGCTTCGCAAA AGAAAACGCAGCAGGCATCCCCATGGACGCAGC AGAACGAAACTACAAAGACCCCAACCACAAACCC GAACTGGTTTTCGCACTGACGCCCTTCCTGGCAA TGAACGCATTCCGAGAATTCTCAGACATCGTTTC ACTGCTGCAGCCCGTTGCAGGCGCACACTCAGC AATCGCACACTTCCTGCAGGTTCCCAACGCAGAA CGACTGTCACAGCTGTTCGCATCACTGCTGAACA TGCAGGGCGAAGAAAAATCACGAGCACTGGCAG TTCTGAAAGCAGCACTGAACTCACAGCAGGGCG AACCCTGGCAGACGATCCGAGTTATCTCAGAAT ACTACCCCGACGACTCAGGCCTGTTCTCACCCCT GCTGCTGAACGTTGTTAAACTGAACCCCGGCGA AGCAATGTTCCTGTTCGCAGAAACGCCCCACGC ATACCTGCAGGGCGTTGCACTGGAAGTTATGGC AAACTCAGACAACGTTCTGCGAGCAGGCCTGAC GCCCAAATACATCGACATCCCCGAACTGGTTGCA AACGTTAAATTCGAACCCAAACCCGCAGGCGAA CTGCTGACGGCACCCGTTAAATCAGGCGCAGAA CTGGACTTCCCCATCCCCGTTGACGACTTCGCAT TCTCACTGCACGACCTGGCACTGCAGGAAACGT CAATCGGCCAGCACTCAGCAGCAATCCTGTTCTG CGTTGAAGGCGAAGCAGTTCTGCGAAAAGACGA ACAGCGACTGGTTCTGAAACCCGGCGAATCAGC ATTCATCGGCGCAGACGAATCACCCGTTAACGC ATCAGGCACGGGCCGACTGGCACGAGTTTACAA CAAACTGTAG

Table S19. All sequences used cont.

Plasmid	gid	name	DNA	GK	Long name	Alternate name	CDS
pJT29	6	manA6	729	461	manA_orig Acc	Accuracy	ATGCAAAAGCTCATAAACTCTGTGCAAAATTATGCATGGGGTAGCAAGACTGCTCTAACAGAATTGTACGGTATAGCCAACCCGCAACAGCAACCTATGGCCGAACTATGGATGGGTGCTCACCCAAAGTCGAGCTCCCGGATAACTACCGCCAACGGAGAGACCGTCTCACTTAGAGATGCGATTGAGAAGAATAAAACCGCCATGCTTGGGGAGGCGGTTGCCAATCGGTTTGGTGAGTTACCATTTCTCTTTAAAGTATTATGTGCCGCCCAGCCACTCTCGATACAGGTGCATCCGAATAAAAGAAATAGTGAGATAGGATTTGCCAAAGAAAACGCAGCAGGGATACCAATGGACGCCGCAGAGCGGAATTATAAAGACCCAAATCACAAGCCCGAGTTAGTGTTCGCCTTAACCCCATTTCTAGCCATGAACGCATTCAGAGAGTTCAGTGATATAGTCAGCCTACTACAGCCAGTCGCTGGAGCCCATAGCGCTATCGCCCACTTTCTTCAGGTGCCAAACGCCGAACGGCTCAGCCAACTTTTTGCGAGTCTATTAAATATGCAGGGTGAGGAAAAGTCGAGGGCACTTGCCGTGCTAAAGGCAGCCCTAAATTCCCAGCAAGGAGAGCCATGGCAAACTATACGCGTCATATCGGAATACTATCCTGACGACTCCGGGCTGTTTAGTCCGCTACTACTTAACGTCGTTAAACTAAATCCGGGCGAAGCGATGTTTTTATTTGCTGAAACCCCTCACGCCTATCTTCAGGGTGTCGCCCTCGAAGTGATGGCTAATTCAGATAATGTCTTACGGGCCGGTCTCACACCAAAGTATATAGACATCCCAGAATTAGTCGCCAACGTGAAGTTCGAACCAAAGCCGGCCGGCGAGCTCTTGACCGCCCCAGTCAAGAGCGGTGCTGAACTAGATTTTCCAATACCAGTCGACGACTTCGCCTTTTCCCTCCATGACCTTGCCCTTCAGGAGACCTCCATAGGACAGCATAGCGCCGCAATACTATTCTGCGTCGAAGGAGAAGCCGTGCTCCGGAAGGATGAGCAACGGCTTGTGTTGAAGCCAGGAGAGAGCGCATTCATAGGAGCCGATGAGAGTCCGGTTAATGCTTCCGGTACCGGTAGACTAGCCAGGGTGTATAACAAACTGTAG

Table S19. All sequences used cont.

Plasmid	gid	name	DNA	GK	Long name	Alternate name	CDS
pJT36	7	manA7	738	524	manaGesch windigkeit	Speed	ATGCAGAAACTGATCAACTCTGTTCAGAACTAC GCATGGGGCTCTAAAACGGCACTGACGGAACTG TACGGCATCGCAAACCCTCAGCAGCAGCCTATG GCAGAACTGTGGATGGGCGCACACCCTAAATCT TCTTCTCGAATCACGACGGCAAACGGCGAAACG GTTTCTCTGCGAGACGCAATCGAAAAAAACAAA ACGGCAATGCTGGGCGAAGCAGTTGCAAACCGA TTCGGCGAACTGCCTTTCCTGTTCAAAGTTCTGT GCGCAGCACAGCCTCTGTCTATCCAGGTTCACCC TAACAAACGAAACTCTGAAATCGGCTTCGCAAA AGAAAACGCAGCAGGCATCCCTATGGACGCAGC AGAACGAAACTACAAAGACCCTAACCACAAACCT GAACTGGTTTTCGCACTGACGCCTTTCCTGGCAA TGAACGCATTCCGAGAATTCTCTGACATCGTTTC TCTGCTGCAGCCTGTTGCAGGCGCACACTCTGC AATCGCACACTTCCTGCAGGTTCCTAACGCAGAA CGACTGTCTCAGCTGTTCGCATCTCTGCTGAACA TGCAGGGCGAAGAAAAATCTCGAGCACTGGCAG TTCTGAAAGCAGCACTGAACTCTCAGCAGGGCG AACCTTGGCAGACGATCCGAGTTATCTCTGAAT ACTACCCTGACGACTCTGGCCTGTTCTCTCCTCT GCTGCTGAACGTTGTTAAACTGAACCCTGGCGA AGCAATGTTCCTGTTCGCAGAAACGCCTCACGC ATACCTGCAGGGCGTTGCACTGGAAGTTATGGC AAACTCTGACAACGTTCTGCGAGCAGGCCTGAC GCCTAAATACATCGACATCCCTGAACTGGTTGC AAACGTTAAATTCGAACCTAAACCTGCAGGCGA ACTGCTGACGGCACCTGTTAAATCTGGCGCAGA ACTGGACTTCCCTATCCCTGTTGACGACTTCGCA TTCTCTCTGCACGACCTGGCACTGCAGGAAACG TCTATCGGCCAGCACTCTGCAGCAATCCTGTTCT GCGTTGAAGGCGAAGCAGTTCTGCGAAAAGACG AACAGCGACTGGTTCTGAAACCTGGCGAATCTG CATTCATCGGCGCAGACGAATCTCCTGTTAACGC ATCTGGCACGGGCCGACTGGCACGAGTTTACAA CAAACTGTAA

Table S19. All sequences used cont.

Plasmid	gid	name	DNA	GK	Long name	Alternate name	CDS
pJT37	8	manA8	740	525	manA_orig AccRamp	Accuracy + Ramp	ATGCAAAAACTCATTAACTCAGTGCAAAACTATGCCTGGGGAAGTAAAACTGCGTTAACGGAACTTTATGGCATCGCCAATCCTCAGCAGCAGCCAATGGCTGAACTCTGGATGGGCGCGCATCCCAAAAGCTCATCGCGAATCACGACCGCAAACGGCGAAACGGTTTCACTGCGAGACGCAATCGAAAAAAACAAAACGGCAATGCTGGGCGAAGCAGTTGCAAACCGATTCGGCGAACTGCCCTTCCTGTTCAAAGTTCTGTGCGCAGCACAGCCCCTGTCAATCCAGGTTCACCCCAACAAACGAAACTCAGAAATCGGCTTCGCAAAAGAAAACGCAGCAGGCATCCCCATGGACGCAGCAGAACGAAACTACAAAGACCCCAACCACAAACCCGAACTGGTTTTCGCACTGACGCCCTTCCTGGCAATGAACGCATTCCGAGAATTCTCAGACATCGTTTCACTGCTGCAGCCCGTTGCAGGCGCACACTCAGCAATCGCACACTTCCTGCAGGTTCCCAACGCAGAACGACTGTCACAGCTGTTCGCATCACTGCTGAACATGCAGGGCGAAGAAAAATCACGAGCACTGGCAGTTCTGAAAGCAGCACTGAACTCACAGCAGGGCGAACCCTGGCAGACGATCCGAGTTATCTCAGAATACTACCCCGACGACTCAGGCCTGTTCTCACCCCTGCTGCTGAACGTTGTTAAACTGAACCCCGGCGAAGCAATGTTCCTGTTCGCAGAAACGCCCCACGCATACCTGCAGGGCGTTGCACTGGAAGTTATGGCAAACTCAGACAACGTTCTGCGAGCAGGCCTGACGCCCAAATACATCGACATCCCCGAACTGGTTGCAAACGTTAAATTCGAACCCAAACCCGCAGGCGAACTGCTGACGGCACCCGTTAAATCAGGCGCAGAACTGGACTTCCCCATCCCCGTTGACGACTTCGCATTCTCACTGCACGACCTGGCACTGCAGGAAACGTCAATCGGCCAGCACTCAGCAGCAATCCTGTTCTGCGTTGAAGGCGAAGCAGTTCTGCGAAAAGACGAACAGCGACTGGTTCTGAAACCCGGCGAATCAGCATTCATCGGCGCAGACGAATCACCCGTTAACGCATCAGGCACGGGCCGACTGGCACGAGTTTACAACAAACTGTAG

Table S19. All sequences used cont.

Plasmid	gid	name	DNA	GK	Long name	Alternate name	CDS
pJT38	9	manA9	742	526	manA_origOpt5050Ramp	Intermediate + Ramp	ATGCAAAAACTCATTAACTCAGTGCAAAACTATGCCTGGGGAAGTAAAACTGCGTTAACGGAACTTTATGGCATCGCCAATCCTCAGCAGCAGCCAATGGCTGAACTCTGGATGGGCGCGCATCCCAAAAGCTCATCGCGAATCACGACCGCCAACGGAGAGACCGTCTCACTTAGAGATGCGATTGAGAAGAATAAAACCGCCATGCTTGGGGAGGCGGTTGCCAATCGGTTTGGTGAGTTACCATTTCTCTTTAAAGTATTATGTGCCGCCCAGCCACTCTCGATACAGGTGCATCCGAATAAAAGAAATAGTGAGATAGGATTTGCCAAAGAAAACGCAGCAGGGATACCAATGGACGCCGCAGAGCGGAATTATAAAGACCCAAATCACAAGCCCGAGTTAGTGTTCGCCTTAACCCCATTTCTAGCCATGAACGCATTCAGAGAGTTCAGTGATATAGTCAGCCTACTACAGCCAGTCGCTGGAGCCCATAGCGCTATCGCCCACTTTCTTCAGGTGCCAAACGCCGAACGGCTCAGCCAACTTTTTGCGAGTCTATTAAATATGCAGGGTGAGGAAAAGTCGAGGGCACTTGCCGTGCTAAAGGCAGCCCTAAATTCCCAGCAAGGAGAGCCATGGCAAACTATACGCGTCATATCGGAATACTATCCTGACGACTCCGGGCTGTTTAGTCCGCTACTACTTAACGTCGTTAAACTAAATCCGGGCGAAGCGATGTTTTTATTTGCTGAAACCCCTCACGCCTATCTTCAGGGTGTCGCCCTCGAAGTGATGGCTAATTCAGATAATGTCTTACGGGCCGGTCTCACACCAAAGTATATAGACATCCCAGAATTAGTCGCCAACGTGAAGTTCGAACCAAAGCCGGCCGGCGAGCTCTTGACCGCCCCAGTCAAGAGCGGTGCTGAACTAGATTTTCCAATACCAGTCGACGACTTCGCCTTTTCCCTCCATGACCTTGCCCTTCAGGAGACCTCCATAGGACAGCATAGCGCCGCAATACTATTCTGCGTCGAAGGAGAAGCCGTGCTCCGGAAGGATGAGCAACGGCTTGTGTTGAAGCCAGGAGAGAGCGCATTCATAGGAGCCGATGAGAGTCCGGTTAATGCTTCCGGTACCGGTAGACTAGCCAGGGTGTATAACAAACTGTAG

Table S19. All sequences used cont.

Plasmid	gid	name	DNA	GK	Long name	Alternate name		CDS
pJT39	10	manA10	744	527	manaGesch<br>windigkeitR<br>amp	Speed<br>Ramp	+	ATGCAAAAACTCATTAACTCAGTGCAAAACTATG<br>CCTGGGGAAGTAAAACTGCGTTAACGGAACTTT<br>ATGGCATCGCCAATCCTCAGCAGCAGCCAATGG<br>CTGAACTCTGGATGGGCGCGCATCCCAAAAGCT<br>CATCGCGAATCACGACCGCAAACGGCGAAACGG<br>TTTCTCTGCGAGACGCAATCGAAAAAAACAAAA<br>CGGCAATGCTGGGCGAAGCAGTTGCAAACCGAT<br>TCGGCGAACTGCCTTTCCTGTTCAAAGTTCTGTG<br>CGCAGCACAGCCTCTGTCTATCCAGGTTCACCCT<br>AACAAACGAAACTCTGAAATCGGCTTCGCAAAA<br>GAAAACGCAGCAGGCATCCCTATGGACGCAGCA<br>GAACGAAACTACAAAGACCCTAACCACAAACCTG<br>AACTGGTTTTCGCACTGACGCCTTTCCTGGCAAT<br>GAACGCATTCCGAGAATTCTCTGACATCGTTTCT<br>CTGCTGCAGCCTGTTGCAGGCGCACACTCTGCA<br>ATCGCACACTTCCTGCAGGTTCCTAACGCAGAAC<br>GACTGTCTCAGCTGTTCGCATCTCTGCTGAACAT<br>GCAGGGCGAAGAAAAATCTCGAGCACTGGCAGT<br>TCTGAAAGCAGCACTGAACTCTCAGCAGGGCGA<br>ACCTTGGCAGACGATCCGAGTTATCTCTGAATA<br>CTACCCTGACGACTCTGGCCTGTTCTCTCCTCTG<br>CTGCTGAACGTTGTTAAACTGAACCCTGGCGAA<br>GCAATGTTCCTGTTCGCAGAAACGCCTCACGCAT<br>ACCTGCAGGGCGTTGCACTGGAAGTTATGGCAA<br>ACTCTGACAACGTTCTGCGAGCAGGCCTGACGC<br>CTAAATACATCGACATCCCTGAACTGGTTGCAAA<br>CGTTAAATTCGAACCTAAACCTGCAGGCGAACT<br>GCTGACGGCACCTGTTAAATCTGGCGCAGAACT<br>GGACTTCCCTATCCCTGTTGACGACTTCGCATTC<br>TCTCTGCACGACCTGGCACTGCAGGAAACGTCT<br>ATCGGCCAGCACTCTGCAGCAATCCTGTTCTGCG<br>TTGAAGGCGAAGCAGTTCTGCGAAAAGACGAAC<br>AGCGACTGGTTCTGAAACCTGGCGAATCTGCAT<br>TCATCGGCGCAGACGAATCTCCTGTTAACGCATC<br>TGGCACGGGCCGACTGGCACGAGTTTACAACAA<br>ACTGTAA
5’utr								AGGTTACTTCATGCGGGTTTCTTGGTTTAATACC<br>TCCCATTGATCTCCACATTGAAACAGGGCTTGAT<br>ACATATG
5’utr promoter								CTCCACATTGAAACAGGGCTTGATACATATG

Table S19. All sequences used cont.

Plasmid	gid	name	DNA	GK	Long name	Alternate name	CDS
pJT15		ova wt	710	435	ova_orig	Wildtype	ATGGGCTCCATCGGCGCAGCAAGCATGGAATTT<br>TGTTTTGATGTATTCAAGGAGCTCAAAGTCCAC<br>CATGCCAATGAGAACATCTTCTACTGCCCCATTG<br>CCATCATGTCAGCTCTAGCCATGGTATACCTGG<br>GTGCAAAAGACAGCACCAGGACACAGATAAATA<br>AGGTTGTTCGCTTTGATAAACTTCCAGGATTCG<br>GAGACAGTATTGAAGCTCAGTGTGGCACATCTG<br>TAAACGTTCACTCTTCACTTAGAGACATCCTCAA<br>CCAAATCACCAAACCAAATGATGTTTATTCGTTC<br>AGCCTTGCCAGTAGACTTTATGCTGAAGAGAGA<br>TACCCAATCCTGCCAGAATACTTGCAGTGTGTG<br>AAGGAACTGTATAGAGGAGGCTTGGAACCTATC<br>AACTTTCAAACAGCTGCAGATCAAGCCAGAGAG<br>CTCATCAATTCCTGGGTAGAAAGTCAGACAAAT<br>GGAATTATCAGAAATGTCCTTCAGCCAAGCTCC<br>GTGGATTCTCAAACTGCAATGGTTCTGGTTAAT<br>GCCATTGTCTTCAAAGGACTGTGGGAGAAAACA<br>TTTAAGGATGAAGACACACAAGCAATGCCTTTC<br>AGAGTGACTGAGCAAGAAAGCAAACCTGTGCAG<br>ATGATGTACCAGATTGGTTTATTTAGAGTGGCA<br>TCAATGGCTTCTGAGAAAATGAAGATCCTGGAG<br>CTTCCATTTGCCAGTGGGACAATGAGCATGTTG<br>GTGCTGTTGCCTGATGAAGTCTCAGGCCTTGAG<br>CAGCTTGAGAGTATAATCAACTTTGAAAAACTG<br>ACTGAATGGACCAGTTCTAATGTTATGGAAGAG<br>AGGAAGATCAAAGTGTACTTACCTCGCATGAAG<br>ATGGAGGAAAAATACAACCTCACATCTGTCTTA<br>ATGGCTATGGGCATTACTGACGTGTTTAGCTCT<br>TCAGCCAATCTGTCTGGCATCTCCTCAGCAGAG<br>AGCCTGAAGATATCTCAAGCTGTCCATGCAGCA<br>CATGCAGAAATCAATGAAGCAGGCAGAGAGGTG<br>GTAGGGTCAGCAGAGGCTGGAGTGGATGCTGCA<br>AGCGTCTCTGAAGAATTTAGGGCTGACCATCCA<br>TTCCTCTTCTGTATCAAGCACATCGCAACCAACG<br>CCGTTCTCTTCTTTGGCAGATGTGTTTCCCCT

Table S19. All sequences used cont.

Plasmid	gid	name	DNA	GK	Long name	Alternate name	CDS
pJT16		ova opt	711	436	ovasyn	Geneart	ATGGGCAGCATTGGAGCAGCTTCCATGGAATTC<br>TGCTTCGACGTGTTCAAAGAGCTGAAAGTCCAC<br>CACGCTAACGAAAACATCTTCTATTGTCCGATCG<br>CCATTATGAGCGCCCTGGCAATGGTTTATCTGG<br>GTGCCAAAGATTCTACCCGTACACAGATTAACAA<br>AGTGGTCCGCTTCGACAAACTGCCTGGTTTTGG<br>TGATAGCATCGAGGCACAGTGTGGTACAAGTGT<br>GAACGTCCATTCTAGCCTGCGTGATATTCTGAAT<br>CAGATTACGAAACCGAACGACGTGTATTCCTTTT<br>CACTGGCCAGTCGTCTGTATGCCGAAGAACGTT<br>ATCCTATTCTGCCGGAGTATCTGCAATGCGTGA<br>AAGAACTGTATCGTGGCGGTCTGGAACCAATCA<br>ATTTTCAAACGGCCGCTGATCAAGCACGTGAAC<br>TGATTAACAGTTGGGTGGAAAGTCAGACCAATG<br>GCATTATCCGTAATGTGCTGCAGCCTAGCAGTG<br>TTGATTCTCAGACGGCAATGGTCCTGGTTAACG<br>CTATTGTGTTTAAAGGCCTGTGGGAGAAAACAT<br>TCAAAGACGAGGATACCCAAGCAATGCCTTTCC<br>GTGTTACCGAGCAGGAAAGCAAACCTGTTCAGA<br>TGATGTATCAAATTGGGCTGTTCCGTGTGGCAA<br>GCATGGCATCCGAAAAAATGAAAATCCTGGAGC<br>TGCCTTTTGCTAGTGGTACAATGAGCATGCTGG<br>TTCTGCTGCCAGATGAAGTTTCAGGTCTGGAGC<br>AACTGGAAAGCATCATCAACTTCGAGAAACTGA<br>CCGAGTGGACCTCTTCTAACGTGATGGAGGAGC<br>GTAAAATCAAAGTCTATCTGCCTCGTATGAAAAT<br>GGAAGAGAAATATAACCTGACCTCCGTGCTGAT<br>GGCTATGGGGATTACTGACGTGTTTAGCAGTAG<br>CGCCAATCTGAGTGGGATTTCAAGCGCTGAGTC<br>TCTGAAAATCTCTCAGGCCGTTCATGCCGCTCAT<br>GCCGAAATCAATGAAGCCGGTCGTGAAGTCGTG<br>GGAAGTGCTGAAGCCGGGGTGGATGCCGCTTCT<br>GTTAGCGAAGAATTTCGTGCCGATCACCCGTTT<br>CTGTTCTGTATCAAACACATTGCTACCAACGCCG<br>TACTGTTTTTTGGACGCTGTGTGAGCCCG

Table S19. All sequences used cont.

Plasmid	gid	name	DNA	GK	Long name	Alternate name	CDS
pJT17		ova1	712	437	ova_origOpt Speed	Deoptimized	ATGGGAAGCATAGGCGCAGCTTCCATGGAATTT TGCTTCGACGTATTTAAGGAGCTAAAGGTCCAT CATGCCAATGAGAATATATTTTATTGTCCAATAG CCATAATGTCCGCCCTAGCCATGGTCTATCTAGG AGCCAAGGATTCCACCCGGACCCAAATAAATAA GGTCGTCCGGTTTGATAAGCTACCAGGATTTGG AGATTCCATAGAGGCCCAATGTGGAACCTCCGT CAATGTCCATTCCTCCCTACGGGATATACTAAAT CAAATAACCAAGCCAAATGATGTCTATTCCTTTT CCCTAGCCTCCCGGCTATATGCCGAGGAGCGGT ATCCAATACTACCAGAGTATCTACAATGTGTCAA GGAGCTATATCGGGGAGGACTAGAGCCAATAAA TTTTCAAACCGCCGCCGATCAAGCCCGGGAGCT AATAAATTCCTGGGTCGAGTCCCAAACCAATGG AATAATACGGAATGTCCTACAACCATCCTCCGTC GATTCCCAAACCGCCATGGTCCTAGTCAATGCCA TAGTCTTTAAGGGACTATGGGAGAAGACCTTTA AGGATGAGGATACCCAAGCCATGCCATTTCGGG TCACCGAGCAAGAGTCCAAGCCAGTCCAAATGA TGTATCAAATAGGACTATTTCGGGTCGCCTCCAT GGCCTCCGAGAAGATGAAGATACTAGAGCTACC ATTTGCCTCCGGAACCATGTCCATGCTAGTCCTA CTACCAGATGAGGTCTCCGGACTAGAGCAACTA GAGTCCATAATAAATTTTGAGAAGCTAACCGAG TGGACCTCCTCCAATGTCATGGAGGAGCGGAAG ATAAAGGTCTATCTACCACGGATGAAGATGGAG GAGAAGTATAATCTAACCTCCGTCCTAATGGCCA TGGGAATAACCGATGTCTTTTCCTCCTCCGCCAA TCTATCCGGAATATCCTCCGCCGAGTCCCTAAAG ATATCCCAAGCCGTCCATGCCGCCCATGCCGAGA TAAATGAGGCCGGACGGGAGGTCGTCGGATCCG CCGAGGCCGGAGTCGATGCCGCCTCCGTCTCCG AGGAGTTTCGGGCCGATCATCCATTTCTATTTTG TATAAAGCATATAGCCACCAATGCCGTCCTATTT TTTGGACGGTGTGTCTCCCCA

Table S19. All sequences used cont.

Plasmid	gid	name	DNA	GK	Long name	Alternate name	CDS
pJT18		ova2	713	438	ova_origOpt Acc	Optimized	ATGGGCTCAATCGGCGCAGCATCAATGGAATTC TGCTTCGACGTTTTCAAAGAACTGAAAGTTCACC ACGCAAACGAAAACATCTTCTACTGCCCCATCGC AATCATGTCAGCACTGGCAATGGTTTACCTGGG CGCAAAAGACTCAACGCGAACGCAGATCAACAA AGTTGTTCGATTCGACAAACTGCCCGGCTTCGG CGACTCAATCGAAGCACAGTGCGGCACGTCAGT TAACGTTCACTCATCACTGCGAGACATCCTGAAC CAGATCACGAAACCCAACGACGTTTACTCATTCT CACTGGCATCACGACTGTACGCAGAAGAACGAT ACCCCATCCTGCCCGAATACCTGCAGTGCGTTAA AGAACTGTACCGAGGCGGCCTGGAACCCATCAA CTTCCAGACGGCAGCAGACCAGGCACGAGAACT GATCAACTCATGGGTTGAATCACAGACGAACGG CATCATCCGAAACGTTCTGCAGCCCTCATCAGTT GACTCACAGACGGCAATGGTTCTGGTTAACGCA ATCGTTTTCAAAGGCCTGTGGGAAAAAACGTTC AAAGACGAAGACACGCAGGCAATGCCCTTCCGA GTTACGGAACAGGAATCAAAACCCGTTCAGATG ATGTACCAGATCGGCCTGTTCCGAGTTGCATCA ATGGCATCAGAAAAAATGAAAATCCTGGAACTG CCCTTCGCATCAGGCACGATGTCAATGCTGGTTC TGCTGCCCGACGAAGTTTCAGGCCTGGAACAGC TGGAATCAATCATCAACTTCGAAAAACTGACGG AATGGACGTCATCAAACGTTATGGAAGAACGAA AAATCAAAGTTTACCTGCCCCGAATGAAAATGG AAGAAAAATACAACCTGACGTCAGTTCTGATGG CAATGGGCATCACGGACGTTTTCTCATCATCAGC AAACCTGTCAGGCATCTCATCAGCAGAATCACTG AAAATCTCACAGGCAGTTCACGCAGCACACGCA GAAATCAACGAAGCAGGCCGAGAAGTTGTTGGC TCAGCAGAAGCAGGCGTTGACGCAGCATCAGTT TCAGAAGAATTCCGAGCAGACCACCCCTTCCTGT TCTGCATCAAACACATCGCAACGAACGCAGTTCT GTTCTTCGGCCGATGCGTTTCACCC

Table S19. All sequences used cont.

Plasmid	gid	name	DNA	GK	Long name	Alternate name	CDS
pJT19		ova3	714	439	ova_orig5050	Intermediate	ATGGGCTCAATCGGCGCTGCTTCCATGGAATTT<br>TGTTTTGACGTATTCAAAGAACTGAAAGTTCACC<br>ATGCCAACGAAAACATCTTCTATTGTCCAATCGC<br>TATCATGTCCGCCCTGGCAATGGTATACCTGGG<br>CGCCAAAGACTCAACAAGGACGCAAATCAATAA<br>GGTGGTTCGATTTGACAAGCTGCCCGGCTTCGG<br>TGACTCGATCGAGGCGCAGTGCGGGACCTCAGT<br>TAATGTTCACTCATCACTGCGCGATATTCTGAAT<br>CAGATTACGAAACCTAATGATGTGTACTCGTTC<br>TCATTGGCATCTCGACTATACGCAGAAGAGCGC<br>TATCCGATCTTACCCGAGTACTTGCAATGCGTG<br>AAAGAGCTTTACCGAGGGGGCCTGGAACCGATC<br>AATTTTCAGACTGCTGCCGACCAAGCTCGAGAG<br>CTTATTAACTCTTGGGTTGAATCACAAACAAACG<br>GAATCATCCGTAATGTACTGCAGCCCTCTTCAGT<br>GGACTCACAAACTGCCATGGTCTTGGTAAATGC<br>GATCGTATTTAAAGGTTTGTGGGAGAAGACTTT<br>CAAAGACGAAGACACACAAGCTATGCCGTTCCG<br>AGTTACGGAACAAGAGTCAAAGCCTGTTCAAAT<br>GATGTATCAAATCGGCTTATTCCGAGTAGCATC<br>GATGGCAAGCGAAAAAATGAAGATCCTGGAGCT<br>GCCTTTCGCATCAGGGACGATGTCAATGTTGGT<br>ATTACTCCCTGATGAAGTCTCAGGTCTGGAACA<br>GCTGGAGTCTATTATCAACTTCGAAAAACTGACC<br>GAATGGACTTCATCGAATGTTATGGAAGAACGC<br>AAAATCAAGGTGTACTTGCCCCGAATGAAGATG<br>GAGGAAAAATATAATCTGACTAGTGTTCTGATG<br>GCGATGGGGATCACAGACGTATTTTCATCGTCT<br>GCTAATTTGAGTGGAATCTCATCGGCTGAGTCG<br>CTAAAGATCTCACAAGCTGTGCACGCGGCACAT<br>GCTGAGATCAACGAGGCGGGGCGAGAAGTAGTT<br>GGTAGTGCTGAAGCGGGGGTTGACGCAGCCTCA<br>GTATCGGAGGAATTCCGTGCCGATCACCCCTTCT<br>TATTTTGCATCAAGCACATTGCAACAAACGCCGT<br>CTTATTCTTTGGGCGATGTGTTTCCCCA

6 Optimized Codon Translation fOr PrOtein Synthesis - OCTOPOS

The Java GUI application OCTOPOS (Optimized Codon Translation fOr PrOtein Synthesis) facilitates optimization of sequences for heterologous expression in *E. coli*, *S. cerevisiae*, and HEK293 cell lines as default expression systems. OCTOPOS allows to configure the parameters of the optimization scheme and to consider alternative expression systems. It uses a slightly simpler variant of the scoring function described in this manuscript, in which function estimates for all features are constrained to linear effects except for the feature GC3 content for which a quadratic approximation was used. OCTOPOS and its software manual are available upon request.

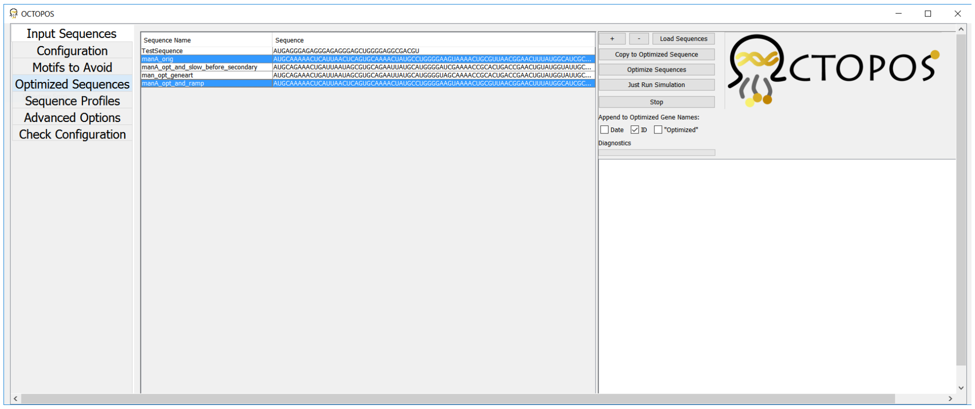

Figure S27. OCTOPOS software

References

1. Lakatos, G. & Chou, T. Totally asymmetric exclusion processes with particles of arbitrary size. *Journal of Physics A: Mathematical and General* **36**, 2027 (2003). URL `http://iopscience.iop.org/0305-4470/36/8/302`.

2. Nakamura, Y., Gojobori, T. & Ikemura, T. Codon usage tabulated from international DNA sequence databases: status for the year 2000. *Nucleic Acids Res.* **28**, 292–292 (2000). URL `http://nar.oxfordjournals.org/content/28/1/292.short`.

3. Rudorf, S., Thommen, M., Rodnina, M. V. & Lipowsky, R. Deducing the kinetics of protein synthesis in vivo from the transition rates measured in vitro. *PLoS Computational Biology* **10**, e1003909 (2014). URL `http://dx.doi.org/10.1371/journal.pcbi.1003909`.

4. Rudorf, S. & Lipowsky, R. Protein Synthesis in E. coli: Dependence of Codon-Specific Elongation on tRNA Concentration and Codon Usage. *PLoS One* **10**, 1–22 (2015).

5. Wang, M. *et al.* PaxDb, a database of protein abundance averages across all three domains of life. *Molecular & Cellular Proteomics* **11**, 492–500 (2012).

6. Siwiak, M. & Zielenkiewicz, P. A comprehensive, quantitative, and genome-wide model of translation. *PLoS Computational Biology* **6**, e1000865 (2010).

7. International Human Genome Sequencing Consortium. Initial sequencing and analysis of the human genome. *Nature* **409**, 860–921 (2001). URL `http://dx.doi.org/10.1038/35057062`.

8. Kramer, E. B. & Farabaugh, P. J. The frequency of translational misreading errors in E. coli is largely determined by tRNA competition. *RNA* **13**, 87–96 (2007). URL `http://rnajournal.cshlp.org/content/13/1/87.short`.

9. von der Haar, T. A quantitative estimation of the global translational activity in logarithmically growing yeast cells. *BMC Systems Biology* **2**, 87 (2008).

10. Kishore, S. *et al.* A quantitative analysis of CLIP methods for identifying binding sites of RNA-binding proteins. *Nat Meth* **8**, 559–564 (2011). URL `http://dx.doi.org/10.1038/nmeth.1608`.

11. Zheng, G. *et al.* Efficient and quantitative high-throughput tRNA sequencing. *Nature Methods* **12**, 835–837 (2015). URL `http://dx.doi.org/10.1038/nmeth.3478`.

12. Geiger, T., Wehner, A., Schaab, C., Cox, J. & Mann, M. Comparative proteomic analysis of eleven common cell lines reveals ubiquitous but varying expression of most proteins. *Molecular & Cellular Proteomics* **11** (2012). URL `http://www.mcponline.org/content/11/3/M111.014050.abstract`. `http://www.mcponline.org/content/11/3/M111.014050.full.pdf+html`.

13. Nagaraj, N. *et al.* System-wide perturbation analysis with nearly complete coverage of the yeast proteome by single-shot ultra HPLC runs on a bench top Orbitrap. *Molecular & Cellular Proteomics* **11**, M111–013722 (2012).

14. Tyson, C. B., Lord, P. G. & Wheals, A. E. Dependency of size of Saccharomyces cerevisiae cells on growth rate. *Journal of Bacteriology* **138**, 92–98 (1979).

15. Billiard, J. *et al.* Quinoline 3-sulfonamides inhibit lactate dehydrogenase A and reverse aerobic glycolysis in cancer cells. *Cancer & Metabolism* **1**, 1–17 (2013). URL `http://dx.doi.org/10.1186/2049-3002-1-19`.

16. Boström, K., Km *et al.* Pulse-chase studies of the synthesis and intracellular transport of apolipoprotein B-100 in HepG2 cells. *Journal of Biological Chemistry* **261**, 13800–13806 (1986). URL `http://www.jbc.org/content/261/29/13800.abstract`.

17. Jørgensen, F. & Kurland, C. G. Processivity errors of gene expression in Escherichia coli. *Journal of molecular biology* **215**, 511–521 (1990).

18. Sin, C., Chiarugi, D. & Valleriani, A. Quantitative assessment of ribosome drop-off in E. coli. *Nucleic Acids Research* **44**, 2528–2537 (2016).

19. Arava, Y., Boas, F. E., Brown, P. O. & Herschlag, D. Dissecting eukaryotic translation and its control by ribosome density mapping. *Nucleic Acids Research* **33**, 2421–2432 (2005).
